# Supplementary material for: Tuberculosis preventive therapy for people living with HIV: A systematic review and network meta-analysis
Source: PLoS Med. 2021 Sep 14;18(9):e1003738. doi: 10.1371/journal.pmed.1003738 (PMC8439495; doi:10.1371/journal.pmed.1003738)
Supplement: S1 File — PRISMA: Checklist of items to include when reporting a systematic review involving an NMA. Material A: Search strategy. Table A: Information extracted in a predefined extraction sheet. Table B: Risk of bias assessment tool adapted from the Revised Cochrane risk-of-bias tool for randomized trials (RoB 2). Table C: Studies excluded after full-text review. Fig A: Risk of bias assessment of the included studies. Fig B: Network graph of TPT regimens for the outcome of incidence of microbiologically confirmed active TB. Table D: NMA of incidence of microbiologically confirmed active TB throughout study duration, by individual TPT regimens. Table E: NMA of incidence of all-cause mortality throughout study duration, by individual TPT regimens. Table F: NMA of risk of grade 3 or worse hepatotoxicity during treatment, by individual TPT regimens. Table G: Treatment completion of each study arm among all the included studies. Table H: Cumulative incidence of drug-resistant TB by treatment arm, among all the included studies. Fig C: Net heat plot of the NMA of incidence of microbiologically confirmed active TB, by individual TPT regimens. Fig D: Net heat plot of the NMA of incidence of all-cause mortality, by individual TPT regimens. Table I: Effect of aggregated TPT regimens on incidence of microbiologically confirmed active TB, stratified by length of posttreatment follow-up. Table J: Effect of aggregated TPT regimens on incidence of all-cause mortality, stratified by posttreatment follow-up time. Table K: Effect of aggregated TPT regimens on incidence of microbiologically confirmed active TB, stratified by study setting TB incidence. Table L: Effect of aggregated TPT regimens on incidence of all-cause mortality, stratified by study setting TB incidence. Table M: Effect of TPT regimens on incidence of microbiologically confirmed active TB, excluding studies with no follow-up or follow-up less than 1 year. Table N: Effect of TPT regimens on incidence of microbiologically confir [file pmed.1003738.s002.docx]

**SUPPLEMENTARY MATERIAL**

Contents

[PRISMA. Checklist of Items to Include When Reporting A Systematic Review Involving a Network Meta-analysis 3](#_Toc78445028)

[Material A. Search strategy. 6](#_Toc78445029)

[Table A. Information extracted in a pre-defined extraction sheet. 8](#_Toc78445030)

[Table B. Risk of bias assessment tool adapted from the Revised Cochrane risk-of-bias tool for randomized trials (RoB 2). 9](#_Toc78445031)

[Table C. Studies excluded after full text review. 10](#_Toc78445032)

[Fig A. Risk of bias assessment of the included studies. 31](#_Toc78445033)

[Fig B. Network graph of TPT regimens for the outcome of incidence of microbiologically confirmed active TB. 32](#_Toc78445034)

[Table D. Network meta-analysis of incidence of microbiologically confirmed active TB throughout study duration, by individual TPT regimens. 33](#_Toc78445035)

[Table E. Network meta-analysis of incidence of all-cause mortality throughout study duration, by individual TPT regimens. 35](#_Toc78445036)

[Table F. Network meta-analysis of risk of grade three or worse hepatotoxicity during treatment, by individual TPT regimens. 37](#_Toc78445037)

[Table G. Treatment completion of each study arm among all the included studies. 39](#_Toc78445038)

[Table H. Cumulative incidence of drug resistant TB by treatment arm, among all the included studies. 41](#_Toc78445039)

[Fig C. Net heat plot of the network meta-analysis of incidence of microbiologically confirmed active TB, by individual TPT regimens. 44](#_Toc78445040)

[Fig D. Net heat plot of the network meta-analysis of incidence of all-cause mortality, by individual TPT regimens. 45](#_Toc78445041)

[Table I. Effect of aggregated TPT regimens on incidence of microbiologically confirmed active TB, stratified by length of post-treatment follow up. 47](#_Toc78445042)

[Table J. Effect of aggregated TPT regimens on incidence of all-cause mortality, stratified by post-treatment follow up time. 48](#_Toc78445043)

[Table K. Effect of aggregated TPT regimens on incidence of microbiologically confirmed active TB, stratified by study setting TB incidence. 49](#_Toc78445044)

[Table L. Effect of aggregated TPT regimens on incidence of all-cause mortality, stratified by study setting TB incidence. 50](#_Toc78445045)

[Table M. Effect of TPT regimens on incidence of microbiologically confirmed active TB, excluding studies with no follow up or follow up less than one year. 51](#_Toc78445046)

[Table N. Effect of TPT regimens on incidence of microbiologically confirmed active TB, excluding studies with a study setting TB incidence of more than 300 per 100 thousand. 53](#_Toc78445047)

[Table O. Effect of TPT regimens on incidence of all-cause mortality, excluding studies with no post-treatment follow up or follow up less than one year. 55](#_Toc78445048)

[Table P. Effect of TPT regimens on incidence of all-cause mortality, excluding studies with a study setting TB incidence of more than 300 per 100 thousand. 57](#_Toc78445049)

[Table Q. Effect of TPT regimens on incidence of all-cause mortality, excluding rifamycin containing studies with a high rate of ART use. 58](#_Toc78445050)

[Table R. Effect of TPT regimens on incidence of microbiologically confirmed and clinically diagnosed TB, stratified by TST/IGRA status. 59](#_Toc78445051)

[Table S. Detailed information of completion rates, completion criteria and methods used to assess adherence to the TPT regimes of interest in the included studies. 60](#_Toc78445052)

[Table T. Effect of aggregated TPT regimens on incidence of all-cause mortality, stratified by the proportion of study participants receiving ART (above, or below 50%). 63](#_Toc78445053)

[Table U. Effect of aggregated TPT regimens on incidence of microbiologically confirmed TB, stratified by the proportion of study participants receiving ART (above, or below 50%). 64](#_Toc78445054)

[Table V. Network meta-analysis of incidence of all-cause mortality throughout study duration, by aggregated TPT regimens and adjusted for the proportion of subjects receiving anti-retroviral therapy. 65](#_Toc78445055)

[Table W. Network meta-analysis of incidence of microbiologically confirmed active TB throughout study duration, by aggregated TPT regimens and adjusted for the proportion of subjects receiving anti-retroviral therapy. 66](#_Toc78445056)

[Table X. Network meta-analysis of all-cause mortality by individual TPT regimens adjusted for ever use of anti-retroviral therapy. 67](#_Toc78445057)

[Table Y. Network meta-analysis of microbiologically confirmed TB by individual TPT regimens, adjusted for ever use of anti-retroviral therapy. 70](#_Toc78445058)

### PRISMA. Checklist of Items to Include When Reporting A Systematic Review Involving a Network Meta-analysis

| **Section/Topic** | **Item #** | **Checklist Item** | **Reported in section** |
| --- | --- | --- | --- |
| **TITLE** |  |  |  |
| Title | 1 | Identify the report as a systematic review *incorporating a network meta-analysis (or related form of meta-analysis).* | Title page |
|  |  |  |  |
| **ABSTRACT** |  |  |  |
| Structured summary | 2 | Provide a structured summary including, as applicable:  **Background:** main objectives  **Methods:** data sources; study eligibility criteria, participants, and interventions; study appraisal; and *synthesis methods, such as network meta-analysis.*  **Results:** number of studies and participants identified; summary estimates with corresponding confidence/credible intervals; *treatment rankings may also be discussed. Authors may choose to summarize pairwise comparisons against a chosen treatment included in their analyses for brevity.*  **Discussion/Conclusions:** limitations; conclusions and implications of findings.  **Other:** primary source of funding; systematic review registration number with registry name. | Abstract |
|  |  |  |  |
| **INTRODUCTION** |  |  |  |
| Rationale | 3 | Describe the rationale for the review in the context of what is already known*, including mention of why a network meta-analysis has been conducted.* | *Introduction* |
| Objectives | 4 | Provide an explicit statement of questions being addressed, with reference to participants, interventions, comparisons, outcomes, and study design (PICOS). | Introduction paragraph #3 |
|  |  |  |  |
| **METHODS** |  |  |  |
| Protocol and registration | 5 | Indicate whether a review protocol exists and if and where it can be accessed (e.g., Web address); and, if available, provide registration information, including registration number. | Methods, Data Sources and Searches, paragraph #1 |
| Eligibility criteria | 6 | Specify study characteristics (e.g., PICOS, length of follow-up) and report characteristics (e.g., years considered, language, publication status) used as criteria for eligibility, giving rationale. *Clearly describe eligible treatments included in the treatment network, and note whether any have been clustered or merged into the same node (with justification).* | Methods, Data Sources and Searches, paragraph #2 |
| Information sources | 7 | Describe all information sources (e.g., databases with dates of coverage, contact with study authors to identify additional studies) in the search and date last searched. | Methods, Data Sources and Searches, paragraph #2 |
| Search | 8 | Present full electronic search strategy for at least one database, including any limits used, such that it could be repeated. | Supplementary material A |
| Study selection | 9 | State the process for selecting studies (i.e., screening, eligibility, included in systematic review, and, if applicable, included in the meta-analysis). | Methods, Study selection, paragraph #1 and 2 |
| Data collection process | 10 | Describe method of data extraction from reports (e.g., piloted forms, independently, in duplicate) and any processes for obtaining and confirming data from investigators. | Methods, Data extraction and quality assessment, paragraph #1 |
| Data items | 11 | List and define all variables for which data were sought (e.g., PICOS, funding sources) and any assumptions and simplifications made. | Methods, Outcomes and Definitions |
| **Geometry of the network** | **S1** | Describe methods used to explore the geometry of the treatment network under study and potential biases related to it. This should include how the evidence base has been graphically summarized for presentation, and what characteristics were compiled and used to describe the evidence base to readers. | Methods, Data Synthesis and Statistical Analysis, paragraph #3 |
| Risk of bias within individual studies | 12 | Describe methods used for assessing risk of bias of individual studies (including specification of whether this was done at the study or outcome level), and how this information is to be used in any data synthesis. | Methods, Data extraction and quality assessment, paragraph #2 |
| Summary measures | 13 | State the principal summary measures (e.g., risk ratio, difference in means). *Also describe the use of additional summary measures assessed, such as treatment rankings and surface under the cumulative ranking curve (SUCRA) values, as well as modified approaches used to present summary findings from meta-analyses.* | Methods, Data Synthesis and Statistical Analysis, paragraph #1 |
| Planned methods of analysis | 14 | Describe the methods of handling data and combining results of studies for each network meta-analysis. This should include, but not be limited to:   - *Handling of multi-arm trials;* - *Selection of variance structure;* - *Selection of prior distributions in Bayesian analyses; and* - *Assessment of model fit.* | Methods, Data Synthesis and Statistical Analysis, paragraph #3 and 6 |
| **Assessment of Inconsistency** | **S2** | Describe the statistical methods used to evaluate the agreement of direct and indirect evidence in the treatment network(s) studied. Describe efforts taken to address its presence when found. | Methods, Data Synthesis and Statistical Analysis, paragraph #3 |
| Risk of bias across studies | 15 | Specify any assessment of risk of bias that may affect the cumulative evidence (e.g., publication bias, selective reporting within studies). | Methods, Data extraction and quality assessment, paragraph #2 |
| Additional analyses | 16 | Describe methods of additional analyses if done, indicating which were pre-specified. This may include, but not be limited to, the following:   - Sensitivity or subgroup analyses; - Meta-regression analyses; - *Alternative formulations of the treatment network; and* - *Use of alternative prior distributions for Bayesian analyses (if applicable).* | Methods, Secondary Analysis, paragraph #1 and 2 |
|  |  |  |  |
| **RESULTS†** |  |  |  |
| Study selection | 17 | Give numbers of studies screened, assessed for eligibility, and included in the review, with reasons for exclusions at each stage, ideally with a flow diagram. | Results, paragraph #1 |
| **Presentation of network structure** | **S3** | Provide a network graph of the included studies to enable visualization of the geometry of the treatment network. | 19 Supplementary figure 2 |
| **Summary of network geometry** | **S4** | Provide a brief overview of characteristics of the treatment network. This may include commentary on the abundance of trials and randomized patients for the different interventions and pairwise comparisons in the network, gaps of evidence in the treatment network, and potential biases reflected by the network structure. | Results, paragraph #2,4 |
| Study characteristics | 18 | For each study, present characteristics for which data were extracted (e.g., study size, PICOS, follow-up period) and provide the citations. | Results, paragraph #2,4, Table1 |
| Risk of bias within studies | 19 | Present data on risk of bias of each study and, if available, any outcome level assessment. | Results, paragraph #3 |
| Results of individual studies | 20 | For all outcomes considered (benefits or harms), present, for each study: 1) simple summary data for each intervention group, and 2) effect estimates and confidence intervals. *Modified approaches may be needed to deal with information from larger networks.* | Results section, Network meta-analysis results |
| Synthesis of results | 21 | Present results of each meta-analysis done, including confidence/credible intervals. *In larger networks, authors may focus on comparisons versus a particular comparator (e.g. placebo or standard care), with full findings presented in an appendix. League tables and forest plots may be considered to summarize pairwise comparisons.* If additional summary measures were explored (such as treatment rankings), these should also be presented. | Results section, Network meta-analysis results |
| **Exploration for inconsistency** | **S5** | Describe results from investigations of inconsistency. This may include such information as measures of model fit to compare consistency and inconsistency models, *P* values from statistical tests, or summary of inconsistency estimates from different parts of the treatment network. | Results section, Network meta-analysis results, paragraph #7 |
| Risk of bias across studies | 22 | Present results of any assessment of risk of bias across studies for the evidence base being studied. | -- |
| Results of additional analyses | 23 | Give results of additional analyses, if done (e.g., sensitivity or subgroup analyses, meta-regression analyses*, alternative network geometries studied, alternative choice of prior distributions for Bayesian analyses,* and so forth). | Results section, Secondary analysis |
|  |  |  |  |
| **DISCUSSION** |  |  |  |
| Summary of evidence | 24 | Summarize the main findings, including the strength of evidence for each main outcome; consider their relevance to key groups (e.g., healthcare providers, users, and policy-makers). | Discussion, paragraph #1 |
| Limitations | 25 | Discuss limitations at study and outcome level (e.g., risk of bias), and at review level (e.g., incomplete retrieval of identified research, reporting bias). *Comment on the validity of the assumptions, such as transitivity and consistency. Comment on any concerns regarding network geometry (e.g., avoidance of certain comparisons).* | Discussion, paragraph #3 and 4 |
| Conclusions | 26 | Provide a general interpretation of the results in the context of other evidence, and implications for future research. | Conclusion |
|  |  |  |  |
| **FUNDING** |  |  |  |
| Funding | 27 | Describe sources of funding for the systematic review and other support (e.g., supply of data); role of funders for the systematic review. This should also include information regarding whether funding has been received from manufacturers of treatments in the network and/or whether some of the authors are content experts with professional conflicts of interest that could affect use of treatments in the network. | **Metadata doc** |

PICOS = population, intervention, comparators, outcomes, study design.

* Text in italics indicateS wording specific to reporting of network meta-analyses that has been added to guidance from the PRISMA statement.

† Authors may wish to plan for use of appendices to present all relevant information in full detail for items in this section.

### Material A. Search strategy.

| 1 | exp Tuberculosis/ |
| --- | --- |
| 2 | exp Latent Tuberculosis/ |
| 3 | exp Tuberculosis, Pulmonary/ |
| 4 | (Tuberc* or TB or LTBI).ti,ab,kw. |
| 5 | 1 OR 2 OR 3 OR 4 |
| 6 | ("Human Immunodeficiency Virus"). ti,ab,kw. |
| 7 | exp Acquired Immunodeficiency Syndrome/ |
| 8 | (HIV or PLHIV or "Patient living with HIV" or "Person living with HIV" or "people living with HIV" or AIDS).ti,ab,kw. |
| 9 | 6 OR 7 OR 8 |
| 10 | ("TB preventive therapy" or "TB preventative therapy" or "LTBI therapy" or "LTBI preventive therapy" or "LTBI preventative therapy" ).ti,ab,kw. |
| 11 | (TB or LTBI adj3 ( prophylaxis or chemoprophylaxis or chemoprevention)).ti, ab,kw. |
| 12 | exp Isoniazid/ |
| 13 | exp Rifampin/ |
| 14 | exp Rifamycins/ |
| 15 | exp Rifabutin/ |
| 16 | exp Pyrazinamide/ |
| 17 | exp Antitubercular Agents/ |
| 18 | (IPT or 3HP or 1HP or H or RIF or RPT or PZA or TPT).ti,ab,kw. |
| 19 | 10 OR 11 OR 12 OR 13 OR 14 OR 15 OR 16 OR 17 OR 18 |
| 20 | (morta*or morb* or death or incidence).ti,ab,kw. |
| 21 | exp drug-related side effects/ |
| 22 | ("adverse events" or "safety" or "adverse drug reaction" or "ADR" or "acquired drug resistance"). ti,ab,kw. |
| 23 | exp drug resistance/ |
| 24 | (drug and resist*).ti,ab,kw. |
| 25 | exp tuberculosis, multidrug-resistant/ |
| 26 | 20 OR 21 OR 22 OR 23 OR 24 OR 25 |
| 27 | 5 AND 9 AND 19 AND 26 |
| 28 | animals/not humans.sh |
| 29 | 27 not 28 |

### Table A. Information extracted in a pre-defined extraction sheet.

| Section | Information extracted |
| --- | --- |
| Study characteristics | Year of publication, year study started, year study ended, study design, type of blinding (if RCT), location of study, study population, sponsored (yes/no), inclusion and exclusion criteria for participants, age range of participants, total number of participants with any reported comorbidities (DM2, malaria, opportunistic diseases, etc.), concomitant use of other medications (any), testing for LTBI method for LTBI testing (if testing yes), ARV use, clinic setting (HIV or TB specific clinic), pyridoxine supplementation, TPT regimen, directly observed therapy (DOT), adherence support measures and use of CPT (cotrimoxazole preventive therapy). |
| Patient-arm characteristics | Patient arm (intervention, control), type of regimen, dose of regimen (mg per dose) , duration of regimen (months), number of doses per week, number of patients in each arm (or followed if cohort), completion criteria, groups of completion – levels of completion, total number completing TPT (as defined by each study), time period allowed for TPT completion, proportion of TPT completion, method used to measure compliance, percent of compliance, total number of males, percent of males, age (mean or median), mean number of years with HIV, CD4+ count ( mean or median), proportion of participants by WHO clinical stage (I, II, III and IV), ARV regime, proportion of participants on ARV, viral load measurement type (log10 or copies/ml), viral load (mean or median), CD4+ cell count (mean or median cells/mmˆ3), proportion of CD4+ cell count by category (<100, 100-200, 201-349, >=350) before TPT initiation, and proportion of participants by CDC clinical classification. |
| Outcomes | Total number of all cause deaths, incidence rate of death per 100PY, total number of TB deaths, incidence rate of TB death per 100PY, total number of active TB diagnosis (microbiologically confirmed, clinically confirmed or no specified method, by arm and by pulmonary or all forms), incidence rate of active TB per 100PY (microbiologically confirmed, clinically confirmed or no specified method, by arm and by pulmonary or all forms), TB free survival end point time (months), proportion of TB free survival, safety population sample size, classification method used for adverse drug reactions, total number of participants with adverse drug reactions, total number of adverse drug reaction events, proportion of participants with a grade 3 or above adverse drug reaction, proportion of participants that present serious adverse drug reactions, proportion of participants that do not complete TPT due to adverse events, proportion of participants with hepatotoxicity, proportion of participants with neuropathy, proportion of participants with flu-like syndrome, proportion of participants with hypersensitivity reactions, proportion of participants with rash, proportion of participants with gastrointestinal intolerance, proportion of participants with grade 3 or above hepatotoxicity, total number of drug resistant cases, incidence of drug resistance, total number of participants lost to follow up, proportion of participants lost to follow up. |

### Table B. Risk of bias assessment tool adapted from the Revised Cochrane risk-of-bias tool for randomized trials (RoB 2).

| Risk of Bias Assessment |
| --- |
| Domain 1. Bias arising from the randomization process and allocation concealment. |
| 1.1 Was the allocation sequence random? |
| 1.2 Was the allocation sequence concealed until participants were enrolled and assigned to interventions? |
| 1.3 Are the baseline characteristics between intervention groups similar, suggesting there was not a problem with the randomization process ? |
| Domain 2. Risk of bias due to deviations in measurement of the outcomes. |
| 2.1 Were outcome assessors blinded to the intervention received by study participants? |
| Domain 3. Bias due to missing outcome data. |
| 3.1 Were data for this outcome available for all, or nearly all (>90% in each group)., participants randomized ? |
| Domain 4. Bias in selection of the reported results. |
| 4.1 Was the numerical result being assessed prespecified and NOT selected from multiple eligible outcome measurements (e.g. scales, definitions, time points) within the outcome domain? |
| If 4.1 No or probably no. 4.3 Was deviation from the analytical plan justified and reasonable? |

### Table C. Studies excluded after full text review.

| \| **First author** \| **Year** \| **Journal** \| **Title** \| **Main reason for exclusion** \| \| --- \| --- \| --- \| --- \| --- \| \| **Adepoju, A. V.** \| 2016 \| American Journal of Tropical Medicine and Hygiene \| Could acceleration towards global 90:90:90 HIV target alone end tb burden among undiagnosed people living with HIV? a four year pre-and post-isoniazid preventive therapy implementation comparative data from comprehensive hospital in northwestern Nigeria \| Conference abstract \| \| **Aemro, A.** \| 2020 \| BMC Infectious Diseases \| Incidence and predictors of tuberculosis occurrence among adults on antiretroviral therapy at Debre Markos referral hospital, Northwest Ethiopia: Retrospective follow-up study \| Retrospective cohort study \| \| **Ahmed, A.** \| 2018 \| BMJ Open \| Incidence and determinants of tuberculosis infection among adult patients with HIV attending HIV care in north-east Ethiopia: a retrospective cohort study \| Retrospective cohort study \| \| **Albuquerque, M. F.** \| 2017 \| Epidemiol Infect \| Predictors of immunodeficiency-related death in a cohort of low-income people living with HIV: a competing risks survival analysis \| Prospective cohort study \| \| **Alemu, A.** \| 2020 \| PLoS ONE \| Incidence and predictors of extrapulmonary tuberculosis among people living with Human Immunodeficiency Virus in Addis Ababa, Ethiopia: A retrospective cohort study \| Retrospective cohort study \| \| **Alemu, A.** \| 2020 \| International Journal of Infectious Diseases \| Incidence and determinants of tuberculosis among HIV-positive individuals in Addis Ababa, Ethiopia: A retrospective cohort study \| Retrospective cohort study \| \| **Alemu, Y. M.** \| 2016 \| PLoS ONE \| High Incidence of Tuberculosis in the Absence of Isoniazid and Cotrimoxazole Preventive Therapy in Children Living with HIV in Northern Ethiopia: A Retrospective Follow-Up Study \| Retrospective cohort study \| \| **Amoakwa, K.** \| 2014 \| Open Forum Infectious Diseases \| Risk Factors for Developing Active Tuberculosis After the Treatment of Latent Tuberculosis in Adults Infected with Human Immunodeficiency Virus \| Secondary analysis of an included report with no additional information of interest \| \| **Antonucci, G.** \| 2001 \| European Respiratory Journal \| Guidelines of tuberculosis preventive therapy for HIV-infected persons: A prospective, multicentre study \| Prospective cohort study \| \| **Aquino, D. S.** \| 2015 \| Cadernos de saude publica \| Factors associated with treatment for latent tuberculosis in persons living with HIV/AIDS \| Prospective cohort study \| \| **Assebe, L. F.** \| 2015 \| BMC Public Health \| The effect of isoniazid preventive therapy on incidence of tuberculosis among HIV-infected clients under pre-ART care, Jimma, Ethiopia: a retrospective cohort study \| Retrospective cohort study \| \| **Atalell, K. A.** \| 2018 \| PLoS ONE \| Survival and predictors of mortality among children co-infected with tuberculosis and human immunodeficiency virus at University of Gondar Comprehensive Specialized Hospital, Northwest Ethiopia. A retrospective follow-up study \| Retrospective cohort study \| \| **Atey, T. M.** \| 2020 \| AIDS Research and Treatment \| Does Isoniazid Preventive Therapy Provide Better Treatment Outcomes in HIV-Infected Individuals in Northern Ethiopia? A Retrospective Cohort Study \| Retrospective cohort study \| \| **Ayele, H.T.** \| 2015 \| BMC Infectious Diseases \| Effect of isoniazid preventive therapy on tuberculosis or death in persons with HIV: A retrospective cohort study \| Retrospective cohort study \| \| **Beshir, M. T.** \| 2019 \| Epidemiology and health \| Incidence and predictors of tuberculosis among HIV-positive children at Adama Referral Hospital and Medical College, Oromia, Ethiopia: a retrospective follow-up study \| Retrospective cohort study \| \| **Bhavnani, D.** \| 2014 \| American Journal of Respiratory and Critical Care Medicine \| Treatment with three months of rifapentine and isoniazid for tuberculosis infection in New York city chest clinics \| Conference abstract \| \| **Bisson, G. P.** \| 2017 \| AIDS \| Risk factors for early mortality on antiretroviral therapy in advanced HIV-infected adults \| No comparison of interest and would not allow indirect comparisons of currently recommended regimes (secondary analysis of the REMEMBER trial [Hosseinipour, 2016], that aimed to identify risk factors for mortality in patients with advanced HIV) \| \| **Breen, R. A. M.** \| 2000 \| AIDS \| Increased incidence of peripheral neuropathy with co-administration of stavudine and isoniazid in HIV-infected individuals \| Case series \| \| **Chaisson, L. H.** \| 2019 \| AIDS \| CD4+ cell count stratification to guide tuberculosis preventive therapy for people living with HIV \| Other intervention than TPT randomized \| \| **Charalambous, S.** \| 2010 \| AIDS \| Association of isoniazid preventive therapy with lower early mortality in individuals on antiretroviral therapy in a workplace programme \| Prospective cohort study \| \| **Churchyard, G. J.** \| 2003 \| Southern African Journal of HIV Medicine \| Efficacy of secondary isoniazid preventive therapy among HIV-infected Southern Africans: Time to change policy? \| Prospective cohort study \| \| **Churchyard, G. J.** \| 2014 \| New England Journal of Medicine \| A trial of mass isoniazid preventive therapy for tuberculosis control \| Results stratified by HIV status not available \| \| **Codecasa, L. R.** \| 2013 \| International Journal of Tuberculosis & Lung Disease \| Isoniazid preventive treatment: predictors of adverse events and treatment completion \| Prospective cohort study \| \| **Costenaro, P.** \| 2016 \| Journal of Acquired Immune Deficiency Syndromes: JAIDS \| Implementation and Operational Research: Implementation of the WHO 2011 Recommendations for Isoniazid Preventive Therapy (IPT) in Children Living With HIV/AIDS: A Ugandan Experience \| Prospective cohort study \| \| **Cranmer, L. M.** \| 2018 \| The Pediatric Infectious Disease Journal \| High Incidence of Tuberculosis Infection in HIV-exposed Children Exiting an Isoniazid Preventive Therapy Trial \| Secondary analysis of an included report with no information of interest \| \| **Cronin, B.** \| 2015 \| Annals of Global Health \| Underutilization of isoniazid drug therapy to prevent TB disease progression in Swaziland \| Conference abstract \| \| **Daley, C. L.** \| 1998 \| American Journal of Respiratory & Critical Care Medicine \| Incidence of tuberculosis in injection drug users in San Francisco: impact of anergy \| Prospective cohort study \| \| **De PHo, A. M. F.** \| 2001 \| AIDS \| Chemoprophylaxis for tuberculosis and survival of HIV-infected patients in Brazil \| Prospective cohort study \| \| **Dooley, K. E.** \| 2020 \| Lancet HIV \| Once-weekly rifapentine and isoniazid for tuberculosis prevention in patients with HIV taking dolutegravir-based antiretroviral therapy: a phase 1/2 trial \| Other study design \| \| **Dravid, A.** \| 2019 \| BMC Infectious Diseases \| Incidence of tuberculosis among HIV infected individuals on long term antiretroviral therapy in private healthcare sector in Pune, Western India \| Retrospective cohort study \| \| **Durovni, B.** \| 2010 \| AIDS \| The implementation of isoniazid preventive therapy in HIV clinics: the experience from the TB/HIV in Rio (THRio) study \| Other intervention than TPT randomized \| \| **Durovni, B.** \| 2013 \| The Lancet Infectious Diseases \| Effect of improved tuberculosis screening and isoniazid preventive therapy on incidence of tuberculosis and death in patients with HIV in clinics in Rio de Janeiro, Brazil: a stepped wedge, cluster-randomised trial \| Other intervention than TPT randomized \| \| **Edessa, D.** \| 2015 \| PLoS ONE \| A Description of Mortality Associated with IPT plus ART Compared to ART Alone among HIV-Infected Individuals in Addis Ababa, Ethiopia: A Cohort Study \| Retrospective cohort study \| \| **Edessa, D.** \| 2014 \| International Journal of Basic Clinical Pharmacology \| Comparison of effects of isoniazid plus ART and ART only regimen on the incidence of tuberculosis and HIV progression in HIV positive patients: a retrospective cohort study at two hospitals in Addis Ababa, Ethiopia \| Retrospective cohort study \| \| **Flynn, A. G.** \| 2019 \| Clinical Infectious Diseases \| Clinical Characteristics of Active Tuberculosis Diagnosed after Starting Treatment for Latent Tuberculosis Infection \| Retrospective cohort study \| \| **Frigati, L. J.** \| 2011 \| Thorax \| The impact of isoniazid preventive therapy and antiretroviral therapy on tuberculosis in children infected with HIV in a high tuberculosis incidence setting \| Secondary analysis of an included report with no information of interest \| \| **Golub, J. E.** \| 2015 \| Clinical Infectious Diseases \| Long-term protection from isoniazid preventive therapy for tuberculosis in HIV-infected patients in a medium-burden tuberculosis setting: the TB/HIV in Rio (THRio) study \| Other intervention than TPT randomized \| \| **Golub, J. E.** \| 2008 \| Journal of Acquired Immune Deficiency Syndromes: JAIDS \| Long-term effectiveness of diagnosing and treating latent tuberculosis infection in a cohort of HIV-infected and at-risk injection drug users \| Prospective cohort study \| \| **Golub, J. E.** \| 2011 \| AIDS \| Isoniazid preventive therapy, HAART and tuberculosis risk in HIV-infected adults in South Africa: A prospective cohort \| Prospective cohort study \| \| **Golub, J. E.** \| 2007 \| AIDS \| The impact of antiretroviral therapy and isoniazid preventive therapy on tuberculosis incidence in HIV-infected patients in Rio de Janeiro, Brazil \| Retrospective cohort study \| \| **Gow, N.** \| 2017 \| International Journal of Tuberculosis & Lung Disease \| Screening for latent tuberculous infection in people living with HIV infection in Auckland, New Zealand \| Retrospective cohort study \| \| **Graham, N. M. H.** \| 1996 \| Archives of Internal Medicine \| Effect of isoniazid chemoprophylaxis on HIV-related mycobacterial disease \| Prospective cohort study \| \| **Grant, A. D.** \| 2005 \| JAMA \| Effect of routine isoniazid preventive therapy on tuberculosis incidence among HIV-infected men in South Africa: a novel randomized incremental recruitment study \| Other study design \| \| **Grant, A. D.** \| 2010 \| AIDS \| Adverse events with isoniazid preventive therapy: experience from a large trial \| Results stratified by HIV status not available \| \| **Gray, D. M.** \| 2014 \| International Journal of Tuberculosis & Lung Disease \| Isoniazid preventive therapy in HIV-infected children on antiretroviral therapy: a pilot study \| Results not available separately for H dosing strategies (pilot factorial trial comparing INH or placebo thrice weekly or daily among children >2 months old). \| \| **Gray, D. M.** \| 2010 \| Journal of Tropical Pediatrics \| Low rates of hepatotoxicity in HIV-infected children on anti-retroviral therapy with and without isoniazid prophylaxis \| Retrospective cohort study \| \| **Gupta, A.** \| 2018 \| Topics in Antiviral Medicine \| Randomized trial of safety of isoniazid preventive therapy during or after pregnancy \| Conference abstract \| \| **Gupta, A.** \| 2017 \| Topics in Antiviral Medicine \| Impact of H adherence on TB incidence and mortality by week 96 in ACTG 5274 trial \| Conference abstract \| \| **Gupta, A.** \| 2019 \| NEJM \| Isoniazid Preventive Therapy in HIV-Infected Pregnant and Postpartum Women \| No comparison of interest and would not allow indirect comparisons of currently recommended regimes (TB APPRISE trial: evaluation of safety of initiating isoniazid preventive therapy during pregnancy, as compared with initiating it during the postpartum period, in women with HIV who are living in areas with a high TB burden) \| \| **Gupta, A.** \| 2007 \| Clinical Infectious Diseases \| Postpartum tuberculosis incidence and mortality among HIV-infected women and their infants in Pune, India, 2002-2005 \| Prospective cohort study \| \| **Gupta, R. K.** \| 2015 \| Lancet HIV \| Does antiretroviral therapy reduce HIV-associated tuberculosis incidence to background rates? A national observational cohort study from England, Wales, and Northern Ireland \| Cohort study using programmatic data \| \| **Hakim, J.** \| 2016 \| Journal of the International AIDS Society \| Enhanced infection prophylaxis reduces mortality in severely immunosuppressed HIV-infected adults and older children initiating antiretroviral therapy in Kenya, Malawi, Uganda and Zimbabwe: The REALITY trial \| Conference abstract \| \| **Hakim, J.** \| 2017 \| NEJM \| Enhanced Prophylaxis plus Antiretroviral Therapy for Advanced HIV Infection in Africa \| No comparison of interest and would not allow indirect comparisons of currently recommended regimes (factorial open-label trial; interventions: enhanced opportunistic infection prophylaxis - immediate INH, trimethoprim-sulfamethoxazole [CPT], fluconazole, azithromycin, albendazole - vs CPT). Both groups received INH. \| \| **Haller, L.** \| 1999 \| Chemotherapy \| Isoniazid plus sulphadoxine-pyrimethamine can reduce morbidity of HIV-positive patients treated for tuberculosis in Africa: a controlled clinical trial \| Secondary prophylaxis \| \| **Halsey, N. A.** \| 1998 \| Lancet \| Randomised trial of isoniazid versus rifampicin and pyrazinamide for prevention of tuberculosis in HIV-1 infection \| No comparison of interest and would not allow indirect comparisons of currently recommended regimes (randomized controlled trial comparing twice a week INH for 6 months vs. RIF with twice a week PZA 2 months) \| \| **Hamlyn, E.** \| 2007 \| Clinical Infectious Diseases \| Reducing tuberculosis incidence in HIV-infected patients by tuberculin skin testing, preventive treatment, and antiretroviral therapy \| Letter to the Editor \| \| **Hanrahan, C.** \| 2015 \| Journal of the International AIDS Society \| The durability of isoniazid preventive therapy for tuberculosis: Long-term follow-up from a prospective cohort of HIV-infected adults in South Africa \| Conference abstract \| \| **Hermans, S. M.** \| 2014 \| Topics in Antiviral Medicine \| Timing of TB episodes after the end of isoniazid preventive therapy: Reinfection or reactivation? \| Conference abstract \| \| **Hermans, S. M.** \| 2016 \| BMC Medicine \| The timing of tuberculosis after isoniazid preventive therapy among gold miners in South Africa: a prospective cohort study \| Results stratified by HIV status not available \| \| **Hesseling, A. C.** \| 2012 \| International Journal of Tuberculosis and Lung Disease \| High prevalence of drug resistance amongst HIV-exposed and -infected children in a tuberculosis prevention trial \| Secondary analysis of an included report with no information of interest \| \| **Hosseinipour, M.** \| 2015 \| Journal of the International AIDS Society \| Empiric TB therapy does not decrease early mortality compared to isoniazid preventive therapy in adults with advanced HIV initiating ART: Results of ACTG A5274 (REMEMBER study) \| Conference abstract \| \| **Hosseinipour, M.** \| 2016 \| Lancet \| Empirical tuberculosis therapy versus isoniazid in adult outpatients with advanced HIV initiating antiretroviral therapy (REMEMBER): a multicountry open-label randomised controlled trial \| No comparison of interest and would not allow indirect comparisons of currently recommended regimes (open-label randomised clinical trial comparing empirical tuberculosis therapy with isoniazid preventive therapy in HIV-positive outpatients initiating antiretroviral therapy with CD4 cell counts of less than 50 cells per μL) \| \| **Howard, A. A.** \| 2017 \| Contemporary Clinical Trials Communications \| The ENRICH Study to evaluate the effectiveness of a combination intervention package to improve isoniazid preventive therapy initiation, adherence and completion among people living with HIV in Ethiopia: rationale and design of a mixed methods cluster randomized trial \| Other intervention than TPT randomized \| \| **Iro, M. A.** \| 2013 \| Archives of disease in childhood: education and practice edition \| Isoniazid prophylaxis started at 3-4 months of life does not prevent tuberculosis disease or infection in both HIV-infected and uninfected children \| Letter to the Editor \| \| **Jansa, J. M.** \| 1998 \| International Journal of Tuberculosis & Lung Disease \| Influence of the human immunodeficiency virus in the incidence of tuberculosis in a cohort of intravenous drug users: effectiveness of anti-tuberculosis chemoprophylaxis \| Prospective cohort study \| \| **Jerene, D.** \| 2017 \| International Journal of Tuberculosis & Lung Disease \| Tuberculosis along the continuum of HIV care in a cohort of adolescents living with HIV in Ethiopia \| Retrospective cohort study \| \| **Johnson Jr, W. D.** \| 1994 \| Annals of internal medicine \| Isoniazid prophylaxis for tuberculosis in HIV infection \| Commentary \| \| **Kabali, C.** \| 2011 \| International Journal of Tuberculosis & Lung Disease \| Completion of isoniazid preventive therapy and survival in HIV-infected, TST-positive adults in Tanzania \| Prospective cohort study \| \| **Kalk, E.** \| 2020 \| Clinical Infectious Diseases \| Safety and Effectiveness of Isoniazid Preventive Therapy in HIV-Positive Pregnant Women on Art: An Observational Study using Linked Population Data \| An observational study that uses linked population data \| \| **Khan, A. R.** \| 2005 \| Thorax \| A clinic service offering isoniazid preventive therapy to HIV infected adults reduces the incidence of tuberculosis \| Letter to the Editor \| \| **Khawcharoenporn, T.** \| 2012 \| International Journal of Tuberculosis & Lung Disease \| Isoniazid preventive therapy and 4-year incidence of pulmonary tuberculosis among HIV-infected Thai patients \| Prospective cohort study \| \| **Kiwuwa-Muyingo, S.** \| 2014 \| AIDS \| Prevalence, incidence and predictors of peripheral neuropathy in African adults with HIV infection within the DART trial \| Other intervention than TPT randomized \| \| **Kritski, A. L.** \| 2000 \| International Journal of Tuberculosis & Lung Disease \| Tuberculosis preventive therapy for HIV-infected persons in less developed countries \| Narrative review \| \| **Kumwenda, J. J.** \| 2016 \| Topics in Antiviral Medicine \| Empiric TB therapy versus IPT in HIV-infected persons initiating art (ACTG A5274 48 W) \| Conference abstract \| \| **Kyaw, N. T. T.** \| 2019 \| International Journal of Tuberculosis & Lung Disease \| IPT in people living with HIV in Myanmar: a five-fold decrease in incidence of TB disease and all-cause mortality \| Retrospective cohort study \| \| **LaCourse, S. M.** \| 2019 \| Journal of Acquired Immune Deficiency Syndromes: JAIDS \| Brief Report: High Programmatic Isoniazid Preventive Therapy (IPT) Use in Pregnancy Among HIV-Infected Women \| Cross-sectional study \| \| **Le Roux, S. M.** \| 2009 \| BMC Medicine \| Adherence to isoniazid prophylaxis among HIV-infected children: a randomized controlled trial comparing two dosing schedules \| Secondary analysis of an included report with no information of interest \| \| **Le Roux, S. M.** \| 2013 \| International Journal of Tuberculosis & Lung Disease \| Safety of long-term isoniazid preventive therapy in children with HIV: a comparison of two dosing schedules \| Secondary analysis of an included report with no information of interest \| \| **Lim, H. J.** \| 2006 \| HIV Clinical Trials \| Effect of tuberculosis preventive therapy on HIV disease progression and survival in HIV-infected adults \| Secondary analysis of an included report with no information of interest \| \| **Loddenkemper, R.** \| 2013 \| International Journal of Tuberculosis & Lung Disease \| Prolonged isoniazid prevention in HIV-positive children: hepatotoxicity is not a major problem \| Letter to the Editor \| \| **Lopez, G.** \| 2011 \| Revista Espanola de Sanidad Penitenciaria \| 10 years of innovation in the treatment of latent tuberculosis infection: a comparison between standard and short course therapies in directly observed therapy \| Retrospective cohort study \| \| **Maharaj, B.** \| 2017 \| International Journal of Tuberculosis & Lung Disease \| Implementing isoniazid preventive therapy in a tuberculosis treatment-experienced cohort on ART \| Prospective cohort study \| \| **Martinez-Pino, I.** \| 2013 \| International Journal of Tuberculosis & Lung Disease \| Incidence of tuberculosis in HIV-infected patients in Spain: The impact of treatment for LTBI \| Prospective cohort study \| \| **Masini, E.** \| 2013 \| Sexually Transmitted Infections \| Outcomes of isoniazid prophylaxis among HIV-infected children in routine clinical settings in Kenya \| Conference abstract \| \| **Masini, E.** \| 2013 \| Public Health in Action \| Outcomes of isoniazid prophylaxis among HIV-infected children attending routine HIV care in Kenya \| Retrospective cohort study \| \| **MAtteli** \| 1999 \| International Journal of Tuberculosis & Lung Disease \| Tolerability of twice-weekly rifabutin-isoniazid combinations versus daily isoniazid for latent tuberculosis in HIV-infected subjects: a pilot study \| One regime of interest but no indirect comparisons were possible \| \| **Mohammed, A.** \| 2007 \| International Journal of Tuberculosis & Lung Disease \| Randomised controlled trial of isoniazid preventive therapy in South African adults with advanced HIV disease \| No comparison of interest and would not allow indirect comparisons of currently recommended regimes (trial comparing twice a week INH for 12 months with placebo among TST-negative PLHIV) \| \| **Moreno, S.** \| 1997 \| Archives of Internal Medicine \| Isoniazid preventive therapy in human immunodeficiency virus-infected persons. Long-term effect on development of tuberculosis and survival \| Retrospective cohort study \| \| **Mosimaneotsile, B.** \| 2010 \| Journal of Acquired Immune Deficiency Syndromes: JAIDS \| Isoniazid tuberculosis preventive therapy in HIV-infected adults accessing antiretroviral therapy: a Botswana Experience, 2004-2006 \| Secondary analysis of an included report with no information of interest \| \| **Mudzviti, T.** \| 2019 \| Drugs Real World Outcomes \| Tolerability of Isoniazid Preventive Therapy in an HIV-Infected Cohort of Paediatric and Adolescent Patients on Antiretroviral Therapy from a Resource-Limited Setting: A Retrospective Cohort Study \| Retrospective cohort study \| \| **Mueller, Y.** \| 2017 \| Medicine \| Adherence, tolerability, and outcome after 36 months of isoniazid-preventive therapy in 2 rural clinics of Swaziland: A prospective observational feasibility study \| Prospective cohort study \| \| **Mugomeri, E.** \| 2018 \| Journal of the International AIDS Society \| Durability and effectiveness of isoniazid preventive therapy in Lesotho, southern Africa \| Conference abstract \| \| **Mukwiza-Kaponda, M.** \| 2015 \| Southern African Journal of Epidemiology and Infection \| Assessment of the effect of isoniazid prevention therapy (IPT) register in monitoring tuberculosis among HIV positive individuals in Sedibeng district, South Africa: A retrospective study \| Conference abstract \| \| **Munseri, P. J.** \| 2008 \| International Journal of Tuberculosis & Lung Disease \| Completion of isoniazid preventive therapy among HIV-infected patients in Tanzania \| Prospective cohort study \| \| **Mwinga, A.** \| 1998 \| AIDS \| Twice weekly tuberculosis preventive therapy in HIV infection in Zambia \| No comparison of interest and would not allow indirect comparisons of currently recommended regimes (randomized controlled trial comparing isoniazid twice a week vs. 3 months of rifampicin twice a week plus pyrazinamide vs. placebo) \| \| **Nafsiah, S.** \| 2014 \| Respirology \| Evaluation of isoniazid preventive therapy 300 mg per day for 6 months after one-year monitoring in HIV-infected patients in persahabatan hospital Jakarta, Indonesia \| Conference abstract \| \| **Negussie, A.** \| 2018 \| BMC Research Notes \| Tuberculosis co-infection and its associated factors among People living with HIV/AIDS attending antiretroviral therapy clinic in southern Ethiopia: a facility based retrospective study \| Retrospective cohort study \| \| **Ngongondo, M.** \| 2017 \| Topics in Antiviral Medicine \| Hepatotoxicity during IPT and ART in severely immunosuppressed people \| Conference abstract \| \| **Ngongondo, M.** \| 2018 \| Journal of Acquired Immune Deficiency Syndromes \| Hepatotoxicity during isoniazid preventive therapy and antiretroviral therapy in people living with HIV with severe immunosuppression: A secondary analysis of a multi-country open label randomized controlled clinical trial \| No comparison of interest and would not allow indirect comparisons of currently recommended regimes (secondary analysis of the REMEMBER trial [Hosseinipour, 2016] to determine risk factors for hepatotoxicity in severely immunosuppressed individuals taking IPT and ART) \| \| **No authors listed** \| 1996 \| J Int Assoc Physicians AIDS Care \| Isoniazid TB prophylaxis effective in drug users with HIV \| No full-text available \| \| **No authors listed** \| 1999 \| Journal of Respiratory Diseases \| Treating and preventing tuberculosis in HIV-infected patients: Part 2 \| Commentary (clinical update) \| \| **Nyathi, S.** \| 2019 \| PLoS ONE \| Isoniazid preventive therapy: Uptake, incidence of tuberculosis and survival among people living with HIV in Bulawayo, Zimbabwe \| Retrospective cohort study \| \| **Ousley, J.** \| 2018 \| Public Health in Action \| IPT during HIV treatment in Myanmar: high rates of coverage, completion and drug adherence \| Retrospective cohort study \| \| **Panwar, P.** \| 2020 \| The Journal of the Association of Physicians of India \| Incidence of Tuberculosis after Routine IPT (Isoniazid Preventive Therapy) in HIV Infected Patients at a Tertiary Center \| Conference abstract \| \| **Pape, J. W.** \| 1993 \| Lancet \| Effect of isoniazid prophylaxis on incidence of active tuberculosis and progression of HIV infection \| Clinical trial comparing 12H plus B6 against B6 alone. However, 21 of the 60 subjects allocated to B6 alone received INH during the trial and only intention to treat results were presented. \| \| **Pereirasamy, L.** \| 2016 \| European Respiratory Journal \| A retrospective analysis of outcome of Isoniazid preventive therapy in adults living with HIV in Penang, Malaysia \| Conference abstract \| \| **Phyo, K. H.** \| 2019 \| International Journal of Tuberculosis & Lung Disease \| High prevalence and incidence of tuberculosis in people living with the HIV in Mandalay, Myanmar, 2011-2017 \| Prospective cohort study \| \| **portilla, j.** \| 2003 \| Enfermedades Infecciosas y Microbiología Clínica \| Directly observed treatment of latent tuberculosis infection: comparative study of two isoniazid regimens \| One regime of interest but no indirect comparisons were possible. \| \| **Post, F.** \| 2018 \| Topics in antiviral medicine \| Mortality/morbidity after initiating ART with CD4 <100 cells/ul in the reality trial \| Conference abstract \| \| **Post, F. A.** \| 2018 \| Clinical Infectious Diseases \| Causes and Timing of Mortality and Morbidity among Late Presenters Starting Antiretroviral Therapy in the REALITY Trial \| No comparison of interest and would not allow indirect comparisons of currently recommended regimes (Secondary analysis of the REALITY trial [Hakim, 2017] to investigate the contribution and timing of different causes of mortality/morbidity) \| \| **Quigley, M. A.** \| 2001 \| AIDS \| Long-term effect of preventive therapy for tuberculosis in a cohort of HIV-infected Zambian adults \| No comparison of interest \| \| **Rangaka, M. X.** \| 2012 \| Journal of the International AIDS Society \| Randomized controlled trial of isoniazid preventive therapy in HIV-infected persons on antiretroviral therapy \| Conference abstract \| \| **Sabasaba, A.** \| 2019 \| BMC Infectious Diseases \| Effect of isoniazid preventive therapy on tuberculosis incidence and associated risk factors among HIV infected adults in Tanzania: a retrospective cohort study \| Retrospective cohort study \| \| **Sackoff, J. E.** \| 2001 \| International Journal of Tuberculosis & Lung Disease \| TB prevention in HIV clinics in New York City \| Cohort study using programmatic information \| \| **Salazar-Austin, N.** \| 2019 \| Clinical Infectious Diseases \| Isoniazid Preventive Therapy and Pregnancy Outcomes In HIV-Infected Women in the Tshepiso Cohort \| Prospective cohort study \| \| **Sanchez, F.** \| 2002 \| Enfermedades Emergentes \| Treatment of latent tuberculosis infection in injecting drug users co-infected by HIV \| No comparison of interest and would not allow indirect comparisons of currently recommended regimes (trial in PLHIV with TST >=5 mm comparing daily 9H vs. rifampin or rifabutin and PZA for 2 months). Information separated by rifamycin type was not presented. \| \| **Sanchez, V. M.** \| 2001 \| International Journal of Tuberculosis and Lung Disease \| Incidence of tuberculosis and the importance of treatment of latent tuberculosis infection in a Spanish prison population \| Prospective cohort study \| \| **Schlegel, M.** \| 2007 \| Clinical Infectious Diseases \| Reducing tuberculosis incidence by tuberculin skin testing, preventive treatment and antiretroviral therapy in an area of low tuberculosis transmission \| Prospective cohort study \| \| **Semu, M.** \| 2017 \| BMC Infectious Diseases \| Effectiveness of isoniazid preventative therapy in reducing incidence of active tuberculosis among people living with HIV/AIDS in public health facilities of Addis Ababa, Ethiopia: a historical cohort study \| Retrospective cohort study \| \| **Shuter, J.** \| 1996 \| Clinical Infectious Diseases \| Secondary prophylaxis for tuberculosis in patients infected with human immunodeficiency virus [6] \| Letter to the Editor \| \| **Sibanda, T.** \| 2013 \| International Journal of Tuberculosis and Lung Disease \| Anti-tuberculosis treatment outcomes in HIV-infected adults exposed to isoniazid preventive therapy in Botswana. \| Secondary analysis of an included report with no information of interest \| \| **Souza, C. T** \| 2009 \| Memorias do Instituto Oswaldo Cruz \| Effectiveness and safety of isoniazid chemoprophylaxis for HIV-1 infected patients from Rio de Janeiro \| Prospective cohort study \| \| **Sterling, T.** \| 2014 \| Topics in Antiviral Medicine \| Three Months of Weekly Rifapentine + H for M. tuberculosis Infection in HIV-Infected Persons \| Conference abstract \| \| **Sterling, T.** \| 2012 \| Journal of the International AIDS Society \| Tolerability among HIV-positive persons of three months of once-weekly rifapentine + H (3HP) versus 9 months of daily H (9H) for treatment of latent tuberculosis infection: The PREVENT TB Study (TBTC Study 26/ACTG 5259) \| Conference abstract \| \| **Sudre, P.** \| 1997 \| Revue d Epidemiologie et de Sante Publique \| Tuberculin test, antitubercular chemoprophylaxis and incidence of tuberculosis in a Swiss HIV cohort study \| Prospective cohort study \| \| **Swaminathan, S.** \| 2012 \| PLoS ONE \| Efficacy of a six-month versus a 36-month regimen for prevention of tuberculosis in HIV-infected persons in India: a randomized clinical trial \| No comparison of interest and would not allow indirect comparisons of currently recommended regimes (randomized controlled trial of INH plus ethambutol for 6 months vs. 36 months of INH) \| \| **Swindells, S.** \| 2018 \| Topics in Antiviral Medicine \| One month of rifapentine/isoniazid to prevent TB in people with HIV: Brief-TB/A5279 \| Conference abstract \| \| **Taylor, A.** \| 2013 \| Infectious Diseases in Obstetrics & Gynecology \| Pregnancy outcomes in HIV-infected women receiving long-term isoniazid prophylaxis for tuberculosis and antiretroviral therapy \| Secondary analysis of an included report with no information of interest \| \| **Tedla, Z.** \| 2015 \| Chest \| Isoniazid-associated hepatitis in adults infected with HIV receiving 36 months of isoniazid prophylaxis in Botswana \| Secondary analysis of an included report with no information of interest \| \| **Tedla, Z.** \| 2010 \| American Journal of Respiratory & Critical Care Medicine \| Isoniazid-associated hepatitis and antiretroviral drugs during tuberculosis prophylaxis in HIV-infected adults in Botswana \| Secondary analysis of an included report with no information of interest \| \| **Thindwa, D.** \| 2018 \| International Journal of Tuberculosis & Lung Disease \| Completion of isoniazid preventive therapy among human immunodeficiency virus positive adults in urban Malawi \| Prospective cohort study \| \| **Tootla, H. D.** \| 2018 \| Lancet HIV \| Preventing tuberculosis-related death in children with HIV \| Letter to the Editor \| \| **Van Griensven, J.** \| 2015 \| Tropical Medicine & International Health \| Implementation of isoniazid preventive therapy in an HIV clinic in Cambodia: high rates of discontinuation when combined with antiretroviral therapy \| Retrospective cohort study \| \| **Van Halsema, C. L.** \| 2010 \| AIDS \| Tuberculosis outcomes and drug susceptibility in individuals exposed to isoniazid preventive therapy in a high HIV prevalence setting \| Results stratified by HIV status not available \| \| **Walley, J.** \| 1995 \| British Medical Journal \| Chemoprophylaxis in tuberculosis and HIV infection \| Letter to the Editor \| \| **Yirdaw, K. D.** \| 2014 \| PLoS ONE \| Beneficial effect of isoniazid preventive therapy and antiretroviral therapy on the incidence of tuberculosis in people living with HIV in Ethiopia \| Retrospective cohort study \| \| **Zeldow, B.** \| 2017 \| International Journal of Tuberculosis & Lung Disease \| Use of antiretrovirals in HIV-infected children in a tuberculosis prevention trial: IMPAACT P1041 \| Secondary analysis of an included report with no information of interest \| |
| --- | --- | --- | --- | --- | --- | --- | --- | --- | --- | --- | --- | --- | --- | --- | --- | --- | --- | --- | --- | --- | --- | --- | --- | --- | --- | --- | --- | --- | --- | --- | --- | --- | --- | --- | --- | --- | --- | --- | --- | --- | --- | --- | --- | --- | --- | --- | --- | --- | --- | --- | --- | --- | --- | --- | --- | --- | --- | --- | --- | --- | --- | --- | --- | --- | --- | --- | --- | --- | --- | --- | --- | --- | --- | --- | --- | --- | --- | --- | --- | --- | --- | --- | --- | --- | --- | --- | --- | --- | --- | --- | --- | --- | --- | --- | --- | --- | --- | --- | --- | --- | --- | --- | --- | --- | --- | --- | --- | --- | --- | --- | --- | --- | --- | --- | --- | --- | --- | --- | --- | --- | --- | --- | --- | --- | --- | --- | --- | --- | --- | --- | --- | --- | --- | --- | --- | --- | --- | --- | --- | --- | --- | --- | --- | --- | --- | --- | --- | --- | --- | --- | --- | --- | --- | --- | --- | --- | --- | --- | --- | --- | --- | --- | --- | --- | --- | --- | --- | --- | --- | --- | --- | --- | --- | --- | --- | --- | --- | --- | --- | --- | --- | --- | --- | --- | --- | --- | --- | --- | --- | --- | --- | --- | --- | --- | --- | --- | --- | --- | --- | --- | --- | --- | --- | --- | --- | --- | --- | --- | --- | --- | --- | --- | --- | --- | --- | --- | --- | --- | --- | --- | --- | --- | --- | --- | --- | --- | --- | --- | --- | --- | --- | --- | --- | --- | --- | --- | --- | --- | --- | --- | --- | --- | --- | --- | --- | --- | --- | --- | --- | --- | --- | --- | --- | --- | --- | --- | --- | --- | --- | --- | --- | --- | --- | --- | --- | --- | --- | --- | --- | --- | --- | --- | --- | --- | --- | --- | --- | --- | --- | --- | --- | --- | --- | --- | --- | --- | --- | --- | --- | --- | --- | --- | --- | --- | --- | --- | --- | --- | --- | --- | --- | --- | --- | --- | --- | --- | --- | --- | --- | --- | --- | --- | --- | --- | --- | --- | --- | --- | --- | --- | --- | --- | --- | --- | --- | --- | --- | --- | --- | --- | --- | --- | --- | --- | --- | --- | --- | --- | --- | --- | --- | --- | --- | --- | --- | --- | --- | --- | --- | --- | --- | --- | --- | --- | --- | --- | --- | --- | --- | --- | --- | --- | --- | --- | --- | --- | --- | --- | --- | --- | --- | --- | --- | --- | --- | --- | --- | --- | --- | --- | --- | --- | --- | --- | --- | --- | --- | --- | --- | --- | --- | --- | --- | --- | --- | --- | --- | --- | --- | --- | --- | --- | --- | --- | --- | --- | --- | --- | --- | --- | --- | --- | --- | --- | --- | --- | --- | --- | --- | --- | --- | --- | --- | --- | --- | --- | --- | --- | --- | --- | --- | --- | --- | --- | --- | --- | --- | --- | --- | --- | --- | --- | --- | --- | --- | --- | --- | --- | --- | --- | --- | --- | --- | --- | --- | --- | --- | --- | --- | --- | --- | --- | --- | --- | --- | --- | --- | --- | --- | --- | --- | --- | --- | --- | --- | --- | --- | --- | --- | --- | --- | --- | --- | --- | --- | --- | --- | --- | --- | --- | --- | --- | --- | --- | --- | --- | --- | --- | --- | --- | --- | --- | --- | --- | --- | --- | --- | --- | --- | --- | --- | --- | --- | --- | --- | --- | --- | --- | --- | --- | --- | --- | --- | --- | --- | --- | --- | --- | --- | --- | --- | --- | --- | --- | --- | --- | --- | --- | --- | --- | --- | --- | --- | --- | --- | --- | --- | --- | --- | --- | --- | --- | --- | --- | --- | --- | --- | --- | --- | --- | --- | --- | --- | --- | --- | --- | --- | --- | --- | --- | --- | --- | --- | --- | --- | --- | --- | --- | --- | --- | --- | --- | --- | --- | --- | --- | --- | --- | --- | --- | --- | --- | --- | --- | --- | --- | --- | --- | --- | --- | --- | --- | --- | --- | --- | --- | --- | --- | --- | --- | --- | --- | --- | --- | --- | --- | --- | --- | --- | --- | --- | --- | --- | --- | --- | --- | --- | --- | --- | --- | --- | --- | --- | --- | --- | --- | --- | --- | --- | --- | --- | --- | --- | --- | --- | --- | --- | --- | --- | --- | --- | --- | --- | --- | --- | --- | --- | --- | --- | --- | --- | --- | --- | --- | --- | --- | --- | --- | --- | --- | --- | --- | --- | --- | --- | --- | --- | --- | --- | --- | --- | --- | --- | --- | --- | --- | --- | --- | --- | --- |

### Fig A. Risk of bias assessment of the included studies.

**A)**


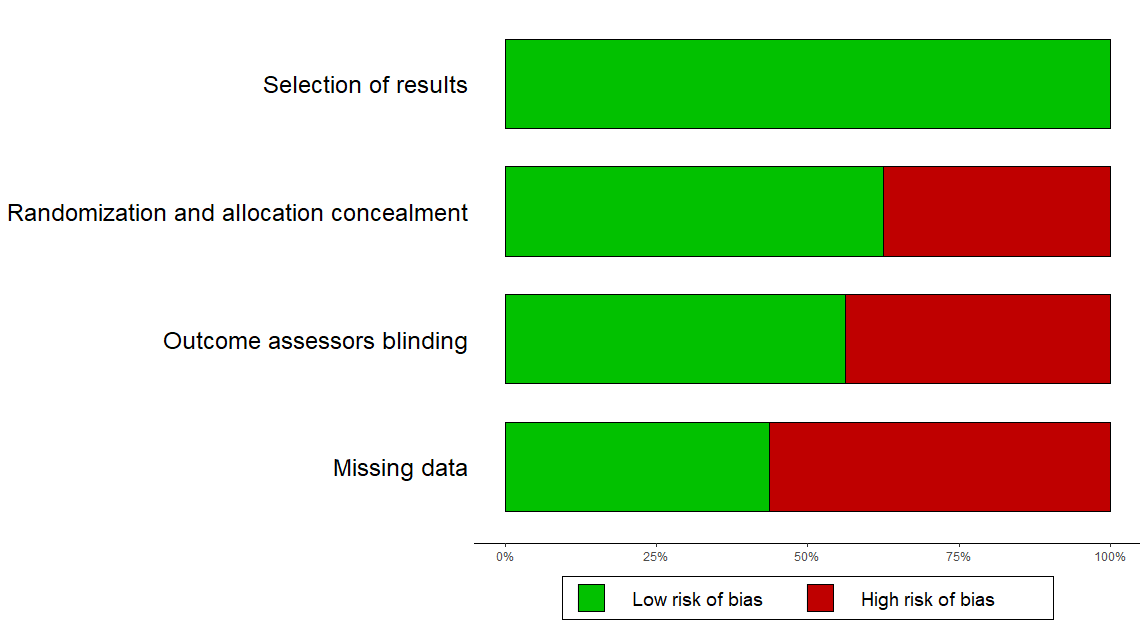


**B)**


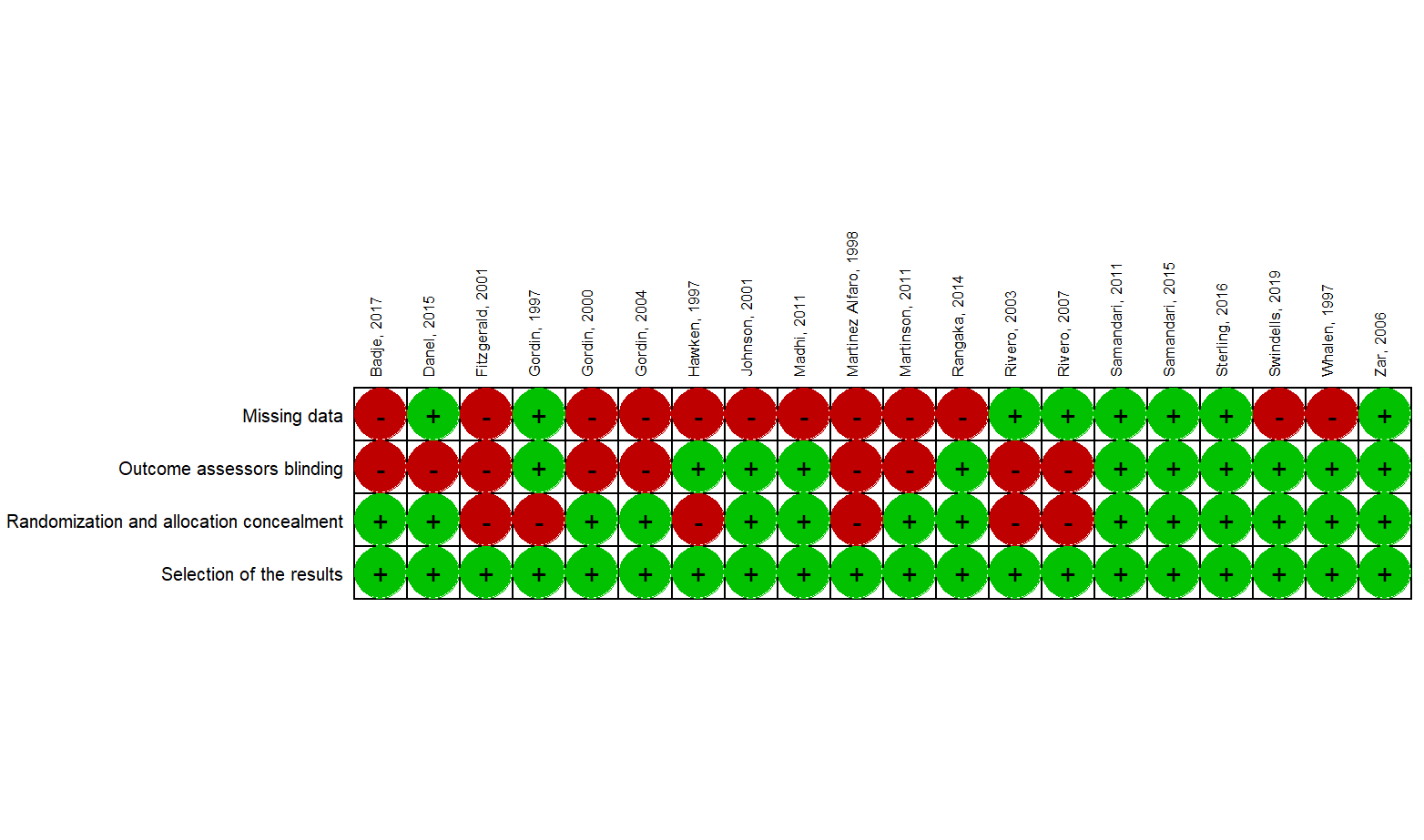


### Fig B. Network graph of TPT regimens for the outcome of incidence of microbiologically confirmed active TB.


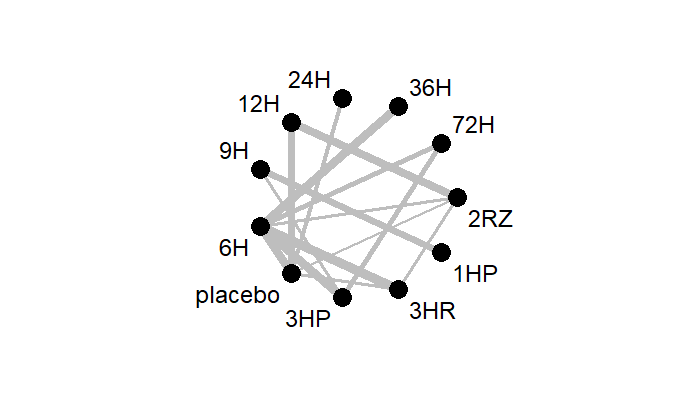


Notes: H, isoniazid; R, rifampicin; P, rifapentine; Z, pyrazinamide; TPT, TB preventive therapy. Nodes depict all the TPT regimens included in the network, with lines showing direct comparisons made within studies. The thickness of the lines is proportional to the inverse standard error of the direct treatment effect of all studies with that comparison.

### Table D. Network meta-analysis of incidence of microbiologically confirmed active TB throughout study duration, by individual TPT regimens.

| **Comparison** | **Number of study arms with direct comparison** | **Direct estimates IRR (95%CI)** | **Indirect estimates IRR (95%CI)** | **Network meta-analysis IRR (95%CI)** |
| --- | --- | --- | --- | --- |
| 6H vs placebo | 3 | 0.8 (0.5 to 1.4) | 1.5 (0.3 to 8) | 0.9 (0.5 to 1.4) |
| 9H vs placebo | 0 | -- | 3.1 (0.5 to 18.8) | 3.1 (0.5 to 18.8) |
| 12H vs placebo | 1 | 0.5 (0.3 to 1.1) | 0.4 (0.1 to 1.8) | 0.5 (0.3 to 1.0) |
| 24H vs placebo | 2 | 0.7 (0.2 to 2.2) | -- | 0.7 (0.2 to 2.2) |
| 36H vs placebo | 0 | -- | 0.6 (0.3 to 1.2) | 0.6 (0.3 to 1.2) |
| 72H vs placebo | 0 | -- | 0.5 (0.2 to 1.5) | 0.5 (0.2 to 1.5) |
| 3HR vs placebo | 1 | 1.0 (0.2 to 4.4) | 0.6 (0.3 to 1.3) | 0.7 (0.4 to 1.3) |
| 3HP vs placebo | 0 | -- | 1.0 (0.4 to 2.2) | 1.0 (0.4 to 2.2) |
| 1HP vs placebo | 0 | -- | 4 (0.6 to 27.5) | 4.0 (0.6 to 27.5) |
| 6H vs 9H | 0 | -- | 0.3 (0 to 1.5) | 0.3 (0 to 1.5) |
| 6H vs 12H | 0 | -- | 1.7 (0.8 to 3.8) | 1.7 (0.8 to 3.8) |
| 6H vs 24H | 0 | -- | 1.2 (0.4 to 3.9) | 1.2 (0.4 to 3.9) |
| 6H vs 36H | 1 | 1.5 (0.9 to 2.6) | -- | 1.5 (0.9 to 2.6) |
| 6H vs 72H | 1 | 1.8 (0.7 to 4.8) | -- | 1.8 (0.7 to 4.8) |
| 6H vs 3HR | 3 | 1.3 (0.8 to 2.2) | 0.5 (0 to 5.9) | 1.3 (0.8 to 2.1) |
| 6H vs 3HP | 1 | 0.9 (0.5 to 1.7) | -- | 0.9 (0.5 to 1.7) |
| 6H vs 1HP | 0 | -- | 0.2 (0 to 1.4) | 0.2 (0 to 1.4) |
| 9H vs 12H | 0 | -- | 6.3 (0.9 to 41.4) | 6.3 (0.9 to 41.4) |
| 9H vs 24H | 0 | -- | 4.3 (0.5 to 34.7) | 4.3 (0.5 to 34.7) |
| 9H vs 36H | 0 | -- | 5.5 (0.9 to 33.4) | 5.5 (0.9 to 33.4) |
| 9H vs 72H | 0 | -- | 6.4 (1.0 to 41.7) | 6.4 (1.0 to 41.7) |
| 9H vs 3HR | 0 | -- | 4.6 (0.8 to 27.6) | 4.6 (0.8 to 27.6) |
| 9H vs 3HP | 1 | 3.2 (0.7 to 16) | -- | 3.2 (0.7 to 16) |
| 9H vs 1HP | 1 | 0.8 (0.4 to 1.6) | -- | 0.8 (0.4 to 1.6) |
| 12H vs 24H | 0 | -- | 0.7 (0.2 to 2.4) | 0.7 (0.2 to 2.4) |
| 12H vs 36H | 0 | -- | 0.9 (0.3 to 2.2) | 0.9 (0.3 to 2.2) |
| 12H vs 72H | 0 | -- | 1 (0.3 to 3.6) | 1.0 (0.3 to 3.6) |
| 12H vs 3HR | 0 | -- | 0.7 (0.3 to 1.8) | 0.7 (0.3 to 1.8) |
| 12H vs 3HP | 0 | -- | 0.5 (0.2 to 1.4) | 0.5 (0.2 to 1.4) |
| 12H vs 1HP | 0 | -- | 0.1 (0 to 0.9) | 0.1 (0 to 0.9) |
| 24H vs 36H | 0 | -- | 1.3 (0.4 to 4.8) | 1.3 (0.4 to 4.8) |
| 24H vs 72H | 0 | -- | 1.5 (0.3 to 7.2) | 1.5 (0.3 to 7.2) |
| 24H vs 3HR | 0 | -- | 1.1 (0.3 to 3.9) | 1.1 (0.3 to 3.9) |
| 24H vs 3HP | 0 | -- | 0.8 (0.2 to 3) | 0.8 (0.2 to 3) |
| 24H vs 1HP | 0 | -- | 0.2 (0 to 1.7) | 0.2 (0 to 1.7) |
| 36H vs 72H | 0 | -- | 1.2 (0.4 to 3.5) | 1.2 (0.4 to 3.5) |
| 36H vs 3HR | 0 | -- | 0.8 (0.4 to 1.7) | 0.8 (0.4 to 1.7) |
| 36H vs 3HP | 0 | -- | 0.6 (0.3 to 1.3) | 0.6 (0.3 to 1.3) |
| 36H vs 1HP | 0 | -- | 0.1 (0 to 1.0) | 0.1 (0 to 1.0) |
| 72H vs 3HR | 0 | -- | 0.7 (0.2 to 2.2) | 0.7 (0.2 to 2.2) |
| 72H vs 3HP | 1 | 0.5 (0.2 to 1.3) | -- | 0.5 (0.2 to 1.3) |
| 72H vs 1HP | 0 | -- | 0.1 (0 to 0.9) | 0.1 (0 to 0.9) |
| 3HR vs 3HP | 0 | -- | 0.7 (0.3 to 1.6) | 0.7(0.3 to 1.6) |
| 3HR vs 1HP | 0 | -- | 0.2 (0 to 1.2) | 0.2 (0 to 1.2) |
| 3HP vs 1HP | 0 | -- | 0.2 (0 to 1.4) | 0.2 (0 to 1.4) |
| **Total number of studies (comparisons)*** | 13 (22) |  |  |  |

Notes: H, isoniazid; R, rifampicin; P, rifapentine; IRR, incidence rate ratio; 95%CI, 95% confidence interval; I^2^ for the network meta-analysis 0% (0.0%; 23.4%); * *Includes studies with 2RZ.

### Table E. Network meta-analysis of incidence of all-cause mortality throughout study duration, by individual TPT regimens.

| **Comparison** | **Number of study arms with direct comparison** | **Direct estimates IRR (95%CI)** | **Indirect estimates IRR (95%CI)** | **Network meta-analysis IRR (95%CI)** |
| --- | --- | --- | --- | --- |
| 6H vs placebo | 5 | 1.0 (0.8 to 1.1) | 2.7 (0.5 to 14.8) | 1.0 (0.8 to 1.1) |
| 9H vs placebo | 0 | -- | 0.6 (0.1 to 2.2) | 0.6 (0.1 to 2.2) |
| 12H vs placebo | 2 | 0.9 (0.5 to 1.5) | 0.6 (0.3 to 1.2) | 0.8 (0.5 to 1.2) |
| 24H vs placebo | 2 | 1.2 (0.7 to 2.1) | -- | 1.2 (0.7 to 2.1) |
| 36H vs placebo | 0 | -- | 1.0 (0.6 to 1.6) | 1.0 (0.6 to 1.6) |
| 72H vs placebo | 0 | -- | 0.6 (0.3 to 1.5) | 0.6 (0.3 to 1.5) |
| 3HR vs placebo | 2 | 0.6 (0.4 to 0.8) | 0.6 (0.3 to 1.4) | 0.6 (0.4 to 0.8) |
| 3HP vs placebo | 0 | -- | 0.6 (0.3 to 1.2) | 0.6 (0.3 to 1.2) |
| 1HP vs placebo | 0 | -- | 0.3 (0.1 to 1.9) | 0.3 (0.1 to 1.9) |
| 6H vs 9H | 0 | -- | 1.7 (0.4 to 6.6) | 1.7 (0.4 to 6.6) |
| 6H vs 12H | 0 | -- | 1.2 (0.8 to 1.9) | 1.2 (0.8 to 1.9) |
| 6H vs 24H | 0 | -- | 0.8 (0.5 to 1.4) | 0.8 (0.5 to 1.4) |
| 6H vs 36H | 1 | 0.9 (0.6 to 1.4) | -- | 0.9 (0.6 to 1.4) |
| 6H vs 72H | 1 | 1.5 (0.7 to 3.4) | -- | 1.5 (0.7 to 3.4) |
| 6H vs 3HR | 3 | 1.8 (1.3 to 2.5) | 1.2 (0.5 to 2.7) | 1.7 (1.2 to 2.3) |
| 6H vs 3HP | 1 | 1.5 (0.8 to 2.9) | -- | 1.5 (0.8 to 2.9) |
| 6H vs 1HP | 0 | -- | 92. (0.5 to 15.7) | 2.9 (0.5 to 15.7) |
| 9H vs 12H | 0 | -- | 0.7 (0.2 to 3) | 0.7 (0.2 to 3) |
| 9H vs 24H | 0 | -- | 0.5 (0.1 to 2) | 0.5 (0.1 to 2) |
| 9H vs 36H | 0 | -- | 0.6 (0.1 to 2.3) | 0.6 (0.1 to 2.3) |
| 9H vs 72H | 0 | -- | 0.9 (0.2 to 3.9) | 0.9 (0.2 to 3.9) |
| 9H vs 3HR | 0 | -- | 1.0 (0.2 to 4) | 1.0 (0.2 to 4) |
| 9H vs 3HP | 1 | 0.9 (0.3 to 3) | -- | 0.9 (0.3 to 3) |
| 9H vs 1HP | 1 | 1.7 (0.6 to 4.7) | -- | 1.7 (0.6 to 4.7) |
| 12H vs 24H | 0 | -- | 0.6 (0.3 to 1.3) | 0.6 (0.3 to 1.3) |
| 12H vs 36H | 0 | -- | 0.8 (0.4 to 1.4) | 0.8 (0.4 to 1.4) |
| 12H vs 72H | 0 | -- | 1.2 (0.5 to 3.1) | 1.2 (0.5 to 3.1) |
| 12H vs 3HR | 1 | 0.7 (0.2 to 2.4) | 1.5 (0.9 to 2.6) | 1.4 (0.8 to 2.2) |
| 12H vs 3HP | 0 | -- | 1.2 (0.6 to 2.7) | 1.2 (0.6 to 2.7) |
| 12H vs 1HP | 0 | -- | 2.3 (0.4 to 13.5) | 2.3 (0.4 to 13.5) |
| 24H vs 36H | 0 | -- | 1.2 (0.6 to 2.4) | 1.2 (0.6 to 2.4) |
| 24H vs 72H | 0 | -- | 1.9 (0.7 to 5.1) | 1.9 (0.7 to 5.1) |
| 24H vs 3HR | 0 | -- | 2.1 (1.1 to 3.9) | 2.1 (1.1 to 3.9) |
| 24H vs 3HP | 0 | -- | 1.9 (0.8 to 4.5) | 1.9 (0.8 to 4.5) |
| 24H vs 1HP | 0 | -- | 3.6 (0.6 to 21.6) | 3.6 (0.6 to 21.6) |
| 36H vs 72H | 0 | -- | 1.6 (0.6 to 4) | 1.6 (0.6 to 4) |
| 36H vs 3HR | 0 | -- | 1.8 (1 to 3) | 1.8 (1 to 3) |
| 36H vs 3HP | 0 | -- | 1.6 (0.7 to 3.5) | 1.6 (0.7 to 3.5) |
| 36H vs 1HP | 0 | -- | 3 (0.5 to 17.5) | 3 (0.5 to 17.5) |
| 72H vs 3HR | 0 | -- | 1.1 (0.5 to 2.6) | 1.1 (0.5 to 2.6) |
| 72H vs 3HP | 1 | 1.0 (0.4 to 2.4) | -- | 1.0 (0.4 to 2.4) |
| 72H vs 1HP | 0 | -- | 1.9 (0.3 to 11.4) | 1.9 (0.3 to 11.4) |
| 3HR vs 3HP | 0 | -- | 0.9 (0.4 to 1.9) | 0.9 (0.4 to 1.9) |
| 3HR vs 1HP | 0 | -- | 1.7 (0.3 to 9.7) | 1.7 (0.3 to 9.7) |
| 3HP vs 1HP | 0 | -- | 1.9 (0.4 to 9.1) | 1.9 (0.4 to 9.1) |
| **Total number of studies (comparisons)*** | 16 (27) |  |  |  |

Notes: IRR, incidence rate ratio; 95%CI, 95% confidence interval; I^2^ for the network meta-analysis 11.7% (0.0%; 51.4%); Person-years of follow up were used as denominator; *Includes studies with 2RZ.

### Table F. Network meta-analysis of risk of grade three or worse hepatotoxicity during treatment, by individual TPT regimens.

| **Comparison** | **Number of study arms with direct comparison** | **Direct estimates RD (95%CI)** | **Indirect estimates RD (95%CI)** | **Network meta-analysis RD (95%CI)** |
| --- | --- | --- | --- | --- |
| 6H vs Placebo | 1 | 1.9 (1.9 to 1.9) | -- | 1.9 (1.9 to 1.9) |
| 9H vs Placebo | 0 | -- | 2.9 (2.9 to 3) | 2.9 (2.9 to 3) |
| 12H vs Placebo | 1 | 1.4 (1.4 to 1.4) | -- | 1.4 (1.4 to 1.4) |
| 36H vs Placebo | 0 | -- | -- | 2.8 (2.7 to 2.8) |
| 72H vs Placebo | 0 | -- | 24.4 (24.4 to 24.5) | 24.4 (24.4 to 24.5) |
| 3HR vs Placebo | 0 | -- | -10.7 (-10.8 to -10.6) | -10.7 (-10.8 to -10.6) |
| 3HP vs Placebo | 0 | -- | -2.1 (-2.1 to -2) | -2.1 (-2.1 to -2) |
| 6H vs 12H | 0 | -- | 0.5 (0.5 to 0.6) | 0.5 (0.5 to 0.6) |
| 6H vs 36H | 1 | -0.9 (-0.9 to -0.9) | -- | -0.9 (-0.9 to -0.9) |
| 6H vs 72H | 1 | -22.5 (-22.6 to -22.5) | -- | -22.5 (-22.6 to -22.5) |
| 6H vs 3HR | 0 |  | 12.6 (12.5 to 12.7) | 12.6 (12.5 to 12.7) |
| 6H vs 3HP | 1 | 4 (4 to 4) | -- | 4 (4 to 4) |
| 9H vs 12H | 0 | -- | 1.5 (1.5 to 1.6) | 1.5 (1.5 to 1.6) |
| 9H vs 36H | 0 | -- | 0.1 (0.1 to 0.2) | 0.1 (0.1 to 0.2) |
| 9H vs 72H | 0 | -- | -21.5 (-21.6 to -21.4) | -21.5 (-21.6 to -21.4) |
| 9H vs 3HR | 0 | -- | 13.6 (13.5 to 13.8) | 1 (1 to 1.1) |
| 9H vs 3HP | 1 | 5 (5 to 5) | -- | 5 (5 to 5) |
| 12H vs 36H | 0 | -- | -1.4 (-1.4 to -1.4) | -1.4 (-1.4 to -1.4) |
| 12H vs 72H | 0 | -- | -23.1 (-23.2 to -23) | -23.1 (-23.2 to -23) |
| 12H vs 3HR | 1 | 12.1 (12 to 12.2) | -- | 12.1 (12 to 12.2) |
| 12H vs 3HP | 0 | -- | 3.5 (3.4 to 3.5) | 3.5 (3.4 to 3.5) |
| 36H vs 72H | 0 | -- | -21.7 (-21.7 to -21.6) | -21.7 (-21.7 to -21.6) |
| 36H vs 3HR | 0 | -- | 13.5 (13.4 to 13.6) | 13.5 (13.4 to 13.6) |
| 36H vs 3HP | 0 | -- | 4.9 (4.8 to 4.9) | 4.9 (4.8 to 4.9) |
| 72H vs 3HR | 0 | -- | 35.2 (35 to 35.3) | 22.5 (22.5 to 22.6) |
| 72H vs 3HP | 1 | 26.5 (26.5 to 26.6) | -- | 26.5 (26.5 to 26.6) |
| 3HR vs 3HP |  | -- | -8.6 (-8.8 to -8.5) | -8.6 (-8.8 to -8.5) |
| **Total number of studies (comparisons)*** | 7 (9) |  |  |  |

Notes: RD, risk difference; 95%CI, 95% confidence interval; I^2^ for the network meta-analysis NA, not enough information available about between-design heterogeneity; * Includes studies with 2RZ. The studies by Temprano group, et al [1]. and Swindells, et al [2]. were excluded from this analysis given that adverse events included events during post treatment follow-up.

### Table G. Treatment completion of each study arm among all the included studies.

| First author (year of publication) | TPT regimen | Number of patients in arm | Completion criteria | Proportion of treatment completion % (n/N) |
| --- | --- | --- | --- | --- |
| Temprano (2015) | 6H | 518 | Attended all six isoniazid prescription visits | 94 (868/927) |
| Badje (2017) | 6H | 1030 | Attended all six isoniazid prescription visits | 94 (869/927) |
| Gordin (1997) | 6H | 260 | Completed treatment within 12 months | 63 (164/260) |
|  | Placebo | 257 | Completed treatment within 12 months | 63 (162/257) |
| Gordin (2000) ^a^ | 12H | 792 | Not specified | 69% (544/792) |
|  |  |  |  |  |
| Hawken (1997) | 6H | 342 | Patients that missed less than 1 week of treatment | 69 (233/342) |
|  | Placebo | 342 | Patients that missed less than 1 week of treatment | 69 (231/342) |
| Whalen (1997) ^a^ | Placebo | 464 | NI | 90 (418/464) |
|  | 6H | 536 | NI | 92 (491/536) |
|  | 3HR | 556 | NI | 93 (516/556) |
|  | Placebo anergy | 323 | NI | 86 (278/323) |
|  | 6H anergy | 395 | NI | 85 (334/395) |
| Rivero (2003) ^a^ | 6H | 83 | >80% doses | 78 (65/83) |
|  | 3HR | 82 | >80% doses | 84 (69/82) |
| Rivero (2007) ^a^ | 6H | 108 | >80% doses | 64 (69/108) |
|  | 3HR | 103 | >80% doses | 61 (63/103) |
| Madhi (2011) | 24H | 273 | NI | 27 (75/273)* |
|  | Placebo | 274 | NI | 30 (81/274)* |
| Martinez-alfaro (2000) | 3HR | 69 | >80% doses | 63 (44/69) |
|  | 12H | 64 | >80% doses | 57 (37/64) |
| Martinson (2011) ^a^ | 3HP | 328 | >90% of doses | 96 (314/328) |
|  |  |  |  |  |
|  | 72H | 164 | >90% of doses | 43 (71/164)** |
|  | 6H | 327 | >90% of doses | 83.8 (274/327) |
| Rangaka (2014) | 12H | 662 | Completed treatment within 15 months | 83 (550/662) |
|  | Placebo | 667 | Completed treatment within 15 months | 82 (550/667) |
| Swindells (2019) | 1HP | 1496 | Patient-reported adherence to the trial regimen for the duration of the trial. Patients in the 1HP arm were allowed 8 weeks to complete treatment. | 97 (1451/1496) |
|  | 9H | 1504 | Patient-reported adherence to the trial regimen for the duration of the trial. | 90 (1353/1504) |
| Sterling (2016) | 3HP | 206 | At least 11 of 12HP doses within 16 weeks | 89 (183/206) |
|  | 9H | 193 | At least 240 of 270H doses within 52 weeks | 64 (123/193) |
|  |  |  |  |  |

Notes:

* 130/274 in the H and 141/274 in placebo arms did not complete interventions because of study termination;

** Patients in the continuous-isoniazid group took isoniazid for 89.1% of the total follow-up time, 60.4% of patients received daily isoniazid for more than 3 years, and 43.3% for more than 4 years

^a^ Treatment arms considered as not of interest were excluded from this table.

### Table H. Cumulative incidence of drug resistant TB by treatment arm, among all the included studies.

| First author (year of publication) | TPT  regimen | TB culture positive | Number of isolates with drug susceptibility testing | Cumulative incidence of drug resistant TBª % (n/N) | Cumulative incidence of H resistance % (n/N)†† | Cumulative incidence of RIF resistance % (n/N)†† | Full description of drug resistance |
| --- | --- | --- | --- | --- | --- | --- | --- |
| Temprano group (2015) | No treatment | 41* | 40* | 0.9 (9/1026) | 0.5 (5/1026) | 0.3 (3/1026) | 3 MDR 2 H-R 4 Other** |
|  | 6H | -- | -- | 0.5 (5/1030) | 0.4 (4/1030) | 0.1 (1/1030) | 1 MDR 3 H-R 1 Other** |
| Gordin (1997) | 6H | 3 | 3 | 0 | 0 | 0 | -- |
|  | Placebo | 6 | 5 | 0 | 0 | 0 | -- |
| Zar (2010) | Placebo | 5 | 5 | 0 | 0 | 0 | -- |
|  | 24H | 0 | -- | -- | 0 | 0 | -- |
| Hawken (1997) | 6H | 19 | 17 | 0.6 (2/342) | 0.6 (2/342) | 0 | 1 H-R 1 H/STM-R |
|  | Placebo | 22 | 21 | 0 | 0 | 0 | -- |
| Johnson (2001) ^b^ | 6H | 36 | 20 | 0.5 (5/931) | 0.5 (5/931) | 0 | 5 H-R |
|  | 3HR | 17 | NI | 0 | 0 | 0 | -- |
| Rivero (2003) ^b^ | 6H | 3 | 3 | 3.6 (3/83) | 3.6 (3/83) | 1.2 (1/83) | 1 MDR (*M. bovis*) 2 H-R |
|  | 3HR | 3 | 3 | 2.4 (2/82) | 2.4 (2/82) | 2.4 (2/82) | 2 MDR *(M. bovis)* |
|  | No treatment | 4 | 4 | 5.2 (4/77) | 5.2 (4/77) | 0 | 4 H/STM-R |
| Rivero (2007) ^b^ | 6H | 2 | 2 | 0.9 (1/108) | 0.9 (1/108) | 0 | 1 H-R |
|  | 3HR | 4 | 4 | 1 (1/103) | 1 (1/103) | 1 (1/103) | 1 MDR *(M. bovis)* |
| Madhi (2011) | 24H | 5 | 7* | 0.4 (1/274) | 0.4 (1/274) | 0.4 (1/274) | 1 MDR |
|  | Placebo | 6 | -- | 0 | 0 | 0 | -- |
| Martinson (2011) ^b^ | 3HP | 21 | 21 | 0.6 (2/328) | 0.3 (1/328) | 0.6 (2/328) | 1 MDR 1 RIF-R |
|  | 72H | 5 | 5 | 0.6 (1/164) | 0.6 (1/164) | 0.6 (1/164) | 1 MDR |
|  | 6H | 18 | 16 | 0 | 0 | 0 | -- |
| Rangaka (2014) | 12H | 12 | 25* | 0.4 (3/680) | 0.4 (3/680) | 0.1 (1/680) | 1 MDR 2 H-R |
|  | Placebo | 22 | -- | 0.4 (3/689) | 0.4 (3/689) | 0.4 (3/689) | 3 MDR |
| Swindells (2019) | 1HP | 18† | NI | 0.2 (3/1488) | 0.2 (3/1488) | 0.1 (1/1488) | 1 RIF-R 2 H-R |
|  | 9H | 14† | NI | 0.1 (2/1498) | 0.1 (2/1498) | 0.1 (2/1498) | 1 RIF-R 1 H R |
| Sterling (2016) | 3HP | 2 | 2 | 0.5 (1/206) | 0 | 0.5 (1/206) | 1 RIF-R *(M. bovis)* |
|  | 9H | 6 | 6 | 0.5 (1/193) | 0.5 (1/193) | 0.5 (1/193) | 1 H/RIF/STM-R |
| Samandari (2011) | 6H | 33* | 29* | 0.3 (6/1995)* | 0.3 (5/1995)* | 0.1 (2/1995) | *1 RIF/STM-R *4 H-R *1 H/RIF/STM-R |
|  | 36H | -- | -- | -- | -- | -- | -- |
| Samandari (2015) | 6H | 53* | 44* | 0.5 (5/989) | 0.5 (5/989) | 0.1 (1/989) | 4 H-R 1 MDR |
|  | 36H | -- | -- | 0.2 (2/1006) | 0.2 (2/1006) | 0.1 (1/1006) | 1 H-R 1 MDR |
|  |  |  |  |  |  |  |  |
| GORDIN (2000) ^B^ | 12H | 26 | 24 | 0.5 (4/792) | 0.38 (3/792) | 0.13 (1/792) | 1MDR  2 H R  1 STM-R |

Notes: H, isoniazid; MDR, multi-drug resistance (H and RIF resistance); PZA, pyrazinamide; R, resistant; RIF, rifampin; STM, streptomycin.

*Results not available per study arm

**Not specified

† Includes positive cultures or a positive nucleic acid amplification test

ªNot all studies carried out drug susceptibility testing for all first line anti-TB drugs

†† Includes multidrug resistant cases

^b^ Treatment arms considered as not of interest were excluded from this table.

### Fig C. Net heat plot of the network meta-analysis of incidence of microbiologically confirmed active TB, by individual TPT regimens.


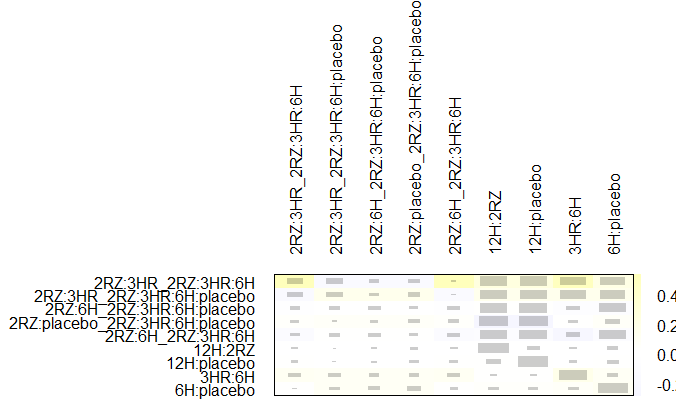


Notes: The net heat plot shows treatment comparisons within designs, treatment comparisons for which there is only one source of evidence are omitted. The grey squares have area proportional to the contribution from the treatment comparison in the column to the treatment comparison in the row. Between design heterogeneity can be assessed by the colors on the diagonal from the top left to bottom right corners. Colors range from red to white, with red indicating a higher level of between design heterogeneity. Colors on the off diagonal indicate the change in design inconsistency when a design is removed. Red colors indicate inconsistency in the evidence between the design in the column to the design in the row. On the other hand, blue and white colors indicate consistency. In this case there is very low between design heterogeneity and inconsistency.

### Fig D. Net heat plot of the network meta-analysis of incidence of all-cause mortality, by individual TPT regimens.


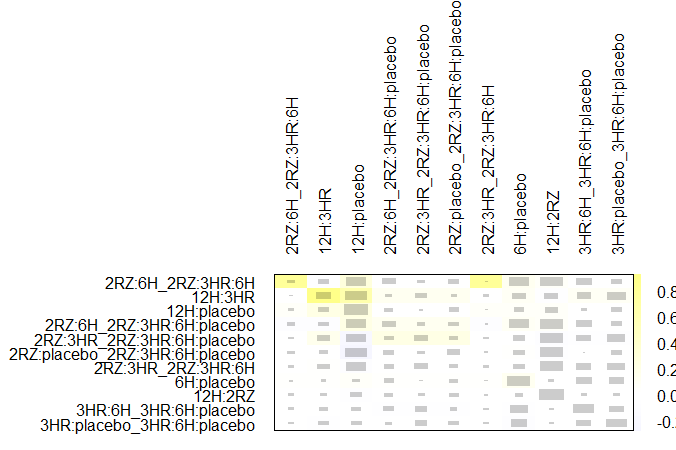


Notes: Low level of heterogeneity and inconsistency. The evidence for the comparison 12H:3HR from the 12H:placebo design shows a low level of inconsistency with evidence from other designs, as shown by the bright yellow colour.

### Table I. Effect of aggregated TPT regimens on incidence of microbiologically confirmed active TB, stratified by length of post-treatment follow up.

| **Comparison** | **Number of study arms with direct comparison** | **Direct estimates IRR (95%CI)** | **Indirect estimates IRR (95%CI)** | **Network meta-analysis IRR (95%CI)** |
| --- | --- | --- | --- | --- |
| **Post-treatment follow up more than one year in all study arms** | | | | |
| **Total number of comparisons (studies)*** | 18 (11) |  |  |  |
| 6 to 12 H vs Placebo | 4 | 0.7 (0.5 to 1.1) | 0.8 (0.1 to 7.3) | 0.7 (0.5 to 1.1) |
| 24 to 72 H vs placebo | 0 | -- | 0.5 (0.2 to 0.9) | 0.5 (0.2 to 0.9) |
| 6 to 12 H vs 24 to 72 H | 1 | 1.5 (0.9 to 2.6) | -- | 1.5 (0.9 to 2.6) |
| 6 to 12 H vs Rifamycin containing | 5 | 1.1 (0.8 to 1.5) | 0.3 (0 to 2.7) | 1.0 (0.8 to 1.5) |
| 24 to 72 H vs Rifamycin containing | 0 | -- | 0.7 (0.4 to 1.3) | 0.7 (0.4 to 1.3) |
| Rifamycin containing vs Placebo | 1 | 1.0 (0.2 to 4.4) | 0.6 (0.4 to 1.1) | 0.7 (0.4 to 1.1) |
| **At least one study arm had no post-treatment follow up or follow up less than one year** | | | | |
| **Total number of comparisons (studies)** | 5 (3) |  |  |  |
| 6 to 12 H vs Placebo | 0 | -- | 1.2 (0.2 to 8.3) | 1.2 (0.2 to 8.3) |
| 24 to 72 H vs placebo | 2 | 0.7 (0.2 to 2.6) | -- | 0.7 (0.2 to 2.6) |
| 6 to 12 H vs 24 to 72 H | 1 | 1.8 (0.4 to 7.1) | -- | 1.8 (0.4 to 7.1) |
| 6 to 12 H vs Rifamycin containing | 1 | 0.9 (0.3 to 2.8) | -- | 0.9 (0.3 to 2.8) |
| 24 to 72 H vs Rifamycin containing | 1 | 0.5 (0.1 to 2) | -- | 0.5 (0.1 to 2) |
| Rifamycin containing vs Placebo | 0 | -- | 1.3 (0.2 to 9.3) | 1.3 (0.2 to 9.3) |

Notes: IRR, incidence rate ratio; 95%CI, 95% confidence interval; I^2^ for network meta-analysis, post-treatment follow up more than one year 0% (0.0% to 42.4%), no post-treatment follow up 16.2% (95%CI for I^2^ not calculable); * Includes studies with 2RZ. Rifamycin containing regimens include 3HR, 3HP and 1HP. Studies with no post-treatment follow up or less than one year: Madhi et al [3], 72 H arm of Martinson et al [4], and Zar et al [5].

### Table J. Effect of aggregated TPT regimens on incidence of all-cause mortality, stratified by post-treatment follow up time.

| **Comparison** | **Number of study arms with direct comparison** | **Direct estimates IRR (95%CI)** | **Indirect estimates IRR (95%CI)** | **Network meta-analysis IRR (95%CI)** |
| --- | --- | --- | --- | --- |
| **Post-treatment follow up more than one year in all study arms** | | | | |
| **Total number of comparisons (studies)*** | 22 (13) |  |  |  |
| 6 to 12 H vs Placebo | 7 | 1.0 (0.8 to 1.1) | 0.7 (0.3 to 1.8) | 0.9 (0.8 to 1.1) |
| 24 to 72 H vs placebo | 0 | -- | 1 (0.7 to 1.5) | 1.0 (0.7 to 1.5) |
| 6 to 12 H vs 24 to 72 H | 1 | 0.9 (0.6 to 1.4) | -- | 0.9 (0.6 to 1.4) |
| 6 to 12 H vs Rifamycin containing | 6 | 1.7 (1.3 to 2.1) | 1.4 (0.5 to 3.4) | 1.6 (1.3 to 2.1) |
| 24 to 72 H vs Rifamycin containing | 0 | -- | 1.7 (1.1 to 2.7) | 1.7 (1.1 to 2.7) |
| Rifamycin containing vs Placebo | 2 | 0.6 (0.4 to 0.8) | 0.6 (0.4 to 1.0) | 0.6 (0.4 to 0.7) |
| **At least one study arm had no post-treatment follow up or follow up less than one year** | | | | |
| **Total number of comparisons (studies)** | 6 (4) |  |  |  |
| 6 to 12 H vs Placebo | 0 | -- | 1.4 (0.2 to 8) | 1.4 (0.2 to 8) |
| 24 to 72 H vs placebo | 2 | 1.0 (0.4 to 2.9) | -- | 1.0 (0.4 to 2.9) |
| 6 to 12 H vs 24 to 72 H | 1 | 1.5 (0.3 to 6.7) | 0.2 (0 to 37.6) | 1.3 (0.3 to 5.5) |
| 6 to 12 H vs Rifamycin containing | 2 | 1.1 (0.4 to 3.4) | -- | 1.1 (0.4 to 3.4) |
| 24 to 72 H vs Rifamycin containing | 1 | 1.1 (0.2 to 4.6) | 0.2 (0 to 21.2) | 0.9 (0.2 to 3.6) |
| Rifamycin containing vs Placebo | 0 | -- | 1.2 (0.2 to 7.2) | 1.2 (0.2 to 7.2) |

Notes: IRR, incidence rate ratio; 95%CI, 95% confidence interval; Person-years of follow up were used as denominator; I^2^ for network meta-analysis, post-treatment follow up more than one year 0% (0.0% to 22.9%), no post-treatment follow up 68% (0.0%; 90.7%); * Includes studies with 2RZ. Rifamycin containing regimens include 3HR, 3HP and 1HP. Studies with no post-treatment follow up or less than one year: Madhi et al [3], 72 H arm of Martinson et al [4], Martinez Alfaro et al [6], and Zar et al [5].

### Table K. Effect of aggregated TPT regimens on incidence of microbiologically confirmed active TB, stratified by study setting TB incidence.

| **Comparison** | **Number of study arms with direct comparison** | **Direct estimates IRR (95%CI)** | **Indirect estimates IRR (95%CI)** | **Network meta-analysis IRR (95%CI)** |
| --- | --- | --- | --- | --- |
| **TB incidence <300 per 100 thousand** | | | | |
| **Total number of comparisons (studies)*** | 14 (7) |  |  |  |
| 6 to 12 H vs Placebo | 2 | 0.7 (0.2 to 1.8) | 0.9 (0.1 to 8.2) | 0.7 (0.3 to 1.8) |
| 6 to 12 H vs Rifamycin containing | 5 | 1.2 (0.8 to 1.8) | 0.2 (0 to 2.4) | 1.1 (0.8 to 1.7) |
| Rifamycin containing vs Placebo | 1 | 1.0 (0.2 to 4.4) | 0.4 (0.1 to 1.6) | 0.6 (0.2 to 1.6) |
| **TB incidence ≥300 per 100 thousand** | | | | |
| **Total number of comparisons (studies)** | 8 (6) |  |  |  |
| 6 to 12 H vs Placebo | 2 | 0.7 (0.4 to 1.1) | 1.2 (0.4 to 3.8) | 0.8 (0.5 to 1.2) |
| 24 to 72 H vs placebo | 2 | 0.7 (0.2 to 2.2) | 0.5 (0.2 to 0.9) | 0.5 (0.3 to 0.9) |
| 6 to 12 H vs 24 to 72 H | 2 | 1.6 (1.0 to 2.5) | 1.0 (0.3 to 3.2) | 1.5 (1.0 to 2.3) |
| 6 to 12 H vs Rifamycin containing | 1 | 0.9 (0.5 to 1.7) | 0.6 (0.1 to 4.1) | 0.9 (0.5 to 1.6) |
| 24 to 72 H vs Rifamycin containing | 1 | 0.5 (0.2 to 1.3) | 0.7 (0.3 to 1.7) | 0.6 (0.3 to 1.1) |
| Rifamycin containing vs Placebo | 0 | -- | 0.9 (0.4 to 1.8) | 0.9 (0.4 to 1.8) |

Notes: IRR, incidence rate ratio; 95%CI, 95% confidence interval; I^2^ of network meta-analysis, TB incidence <300 per 100 thousand 0% (0.0% to 58%), TB incidence >= 300 per 100 thousand 0% (0.0%; 71.7%); *Includes studies with 2RZ. Rifamycin containing regimens include 3HR, 3HP and 1HP. Studies with TB incidence >= 300 per 100 thousand: Hawken et al [7], Samandari et al [8], Zar et al [5], Rangaka et al [9], Martinson et al [4], and Madhi et al [3].

### Table L. Effect of aggregated TPT regimens on incidence of all-cause mortality, stratified by study setting TB incidence.

| **Comparison** | **Number of study arms with direct comparison** | **Direct estimates IRR (95%CI)** | **Indirect estimates IRR (95%CI)** | **Network meta-analysis IRR (95%CI)** |
| --- | --- | --- | --- | --- |
| **Tb incidence <300 per 100 thousand** | | | | |
| **Total number of comparisons (studies)*** | 19 (10) |  |  |  |
| 6 to 12 H vs Placebo | 5 | 0.9 (0.8 to 1.1) | 0.5 (0.2 to 1.5) | 0.9 (0.8 to 1.1) |
| 6 to 12 H vs Rifamycin containing | 6 | 1.6 (1.2 to 2.1) | 1.2 (0.4 to 3.3) | 1.6 (1.2 to 2.1) |
| Rifamycin containing vs Placebo | 2 | 0.6 (0.4 to 0.8) | 0.7 (0.4 to 1.2) | 0.6 (0.4 to 0.8) |
| **TB incidence ≥300 per 100 thousand** | | | | |
| **Total number of comparisons (studies)** | 8 (6) |  |  |  |
| 6 to 12 H vs Placebo | 2 | 1.0 (0.6 to 1.6) | 1.3 (0.5 to 2.9) | 1.0 (0.7 to 1.6) |
| 24 to 72 H vs placebo | 2 | 1.1 (0.6 to 2.2) | 0.9 (0.4 to 1.9) | 1.0 (0.6 to 1.7) |
| 6 to 12 H vs 24 to 72 H | 2 | 1.1 (0.6 to 1.9) | 0.8 (0.4 to 1.9) | 1.0 (0.6 to 1.6) |
| 6 to 12 H vs Rifamycin containing | 1 | 1.5 (0.7 to 3.5) | 0.5 (0 to 4.8) | 1.3 (0.6 to 2.9) |
| 24 to 72 H vs Rifamycin containing | 1 | 1.1 (0.4 to 2.7) | 2.3 (0.6 to 9.5) | 1.3 (0.6 to 3) |
| Rifamycin containing vs Placebo | 0 | -- | 0.8 (0.3 to 1.8) | 0.8 (0.3 to 1.8) |

Notes: IRR, incidence rate ratio; 95%CI, 95% confidence interval; Person-years of follow up were used as denominator; I^2^ for network meta-analysis, TB incidence <300 per 100 thousand 0% (0.0% to 43.8%), >=300 per 100 thousand 46.5% (0.0%; 80.4%); *Includes studies with 2RZ. Rifamycin containing regimens include 3HR, 3HP and 1HP. Studies with TB incidence >= 300 per 100 thousand: Hawken et al [7], Samandari et al [8], Zar et al [5], Rangaka et al [9], Martinson et al [4], and Madhi et al [3].

### Table M. Effect of TPT regimens on incidence of microbiologically confirmed active TB, excluding studies with no follow up or follow up less than one year.

| **Comparison** | **Number of study arms with direct comparison** | **Direct estimates IRR (95%CI)** | **Indirect estimates IRR (95%CI)** | **Network meta-analysis IRR (95%CI)** |
| --- | --- | --- | --- | --- |
| **Total number of comparisons (studies)*** | 18 (11) |  |  |  |
| 6H vs placebo | 3 | 0.8 (0.5 to 1.4) | 1.5 (0.3 to 8) | 0.9 (0.5 to 1.4) |
| 9H vs placebo | 0 | -- | 3.1 (0.5 to 18.8) | 3.1 (0.5 to 18.8) |
| 12H vs placebo | 1 | 0.5 (0.3 to 1.1) | 0.4 (0.1 to 1.8) | 0.5 (0.3 to 1.0) |
| 36H vs placebo | 0 | -- | 0.6 (0.3 to 1.2) | 0.6 (0.3 to 1.2) |
| 3HR vs placebo | 1 | 1.0 (0.2 to 4.4) | 0.6 (0.3 to 1.3) | 0.7 (0.4 to 1.3) |
| 3HP vs placebo | 0 | -- | 1.0 (0.4 to 2.2) | 1.0 (0.4 to 2.2) |
| 1HP vs placebo | 0 | -- | 4 (0.6 to 27.5) | 4 (0.6 to 27.5) |
| 6H vs 9H | 0 | -- | 0.3 (0 to 1.5) | 0.3 (0 to 1.5) |
| 6H vs 12H | 0 | -- | 1.7 (0.8 to 3.8) | 1.7 (0.8 to 3.8) |
| 6H vs 36H | 1 | 1.5 (0.9 to 2.6) | -- | 1.5 (0.9 to 2.6) |
| 6H vs 3HR | 3 | 1.3 (0.8 to 2.2) | 0.5 (0 to 5.9) | 1.3 (0.8 to 2.1) |
| 6H vs 3HP | 1 | 0.9 (0.5 to 1.7) | -- | 0.9 (0.5 to 1.7) |
| 6H vs 1HP | 0 | -- | 0.2 (0 to 1.4) | 0.2 (0 to 1.4) |
| 9H vs 12H | 0 | -- | 6.3 (0.9 to 41.4) | 6.3 (0.9 to 41.4) |
| 9H vs 36H | 0 | -- | 5.5 (0.9 to 33.4) | 5.9 (0.9 to 33.4) |
| 9H vs 3HR | 0 | -- | 4.6 (0.8 to 27.6) | 4.6 (0.8 to 27.6) |
| 9H vs 3HP | 1 | 3.2 (0.7 to 16) | -- | 3.2 (0.7 to 16) |
| 9H vs 1HP | 1 | 0.8 (0.4 to 1.6) | -- | 0.8 (0.4 to 1.6) |
| 12H vs 36H | 0 | -- | 0.9 (0.3 to 2.2) | 0.9 (0.3 to 2.2) |
| 12H vs 3HR | 0 | -- | 0.7 (0.3 to 1.8) | 0.7 (0.3 to 1.8) |
| 12H vs 3HP | 0 | -- | 0.5 (0.2 to 1.4) | 0.5 (0.2 to 1.4) |
| 12H vs 1HP | 0 | -- | 0.1 (0 to 0.9) | 0.1 (0 to 0.9) |
| 36H vs 3HR | 0 | -- | 0.8 (0.4 to 1.7) | 0.8 (0.4 to 1.7) |
| 36H vs 3HP | 0 | -- | 0.6 (0.3 to 1.3) | 0.6 (0.3 to 1.3) |
| 36H vs 1HP | 0 | -- | 0.1 (0 to 1.0) | 0.1 (0 to 1) |
| 3HR vs 3HP |  | -- | 0.7 (0.3 to 1.6) | 0.7 (0.3 to 1.6) |
| 3HR vs 1HP | 0 | -- | 0.2 (0 to 1.2) | 0.2 (0 to 1.2) |
| 3HP vs 1HP | 0 | -- | 0.2 (0 to 1.4) | 0.2 (0 to 1.4) |

Notes: IRR, incidence rate ratio; 95%CI, 95% confidence interval; I^2^ for network meta-analysis 0% (0.0% to 1.1%); *Includes studies with 2RZ. Excluded studies: Madhi et al [3], 72 H arm of Martinson et al [4], and Zar et al [5].

### Table N. Effect of TPT regimens on incidence of microbiologically confirmed active TB, excluding studies with a study setting TB incidence of more than 300 per 100 thousand.

| **Comparison** | **Number of study arms with direct comparison** | **Direct estimates IRR (95%CI)** | **Indirect estimates IRR (95%CI)** | **Network meta-analysis IRR (95%CI)** |
| --- | --- | --- | --- | --- |
| **Total number of comparisons (studies)*** | 15 (8) |  |  |  |
| 6H vs placebo | 2 | 0.7 (0.2 to 1.8) | 1.6 (0.1 to 18.4) | 0.8 (0.3 to 2.0) |
| 9H vs placebo | 0 | -- | 2.8 (0.4 to 19.7) | 2.8 (0.4 to 19.7) |
| 12H vs placebo | 0 | -- | 0.3 (0.1 to 1.9) | 0.3 (0.1 to 1.9) |
| 3HR vs placebo | 1 | 1 (0.2 to 4.4) | 0.4 (0.1 to 1.5) | 0.6 (0.2 to 2.5) |
| 3HP vs placebo | 0 | -- | 0.9 (0.3 to 2.7) | 0.9 (0.3 to 2.7) |
| 1HP vs placebo | 0 | -- | 3.5 (0.4 to 28.3) | 3.5 (0.4 to 28.3) |
| 6H vs 9H | 0 | -- | 0.3 (0 to 1.5) | 0.3 (0 to 1.5) |
| 6H vs 12H | 0 | -- | 2.3 (0.5 to 11.6) | 2.3 (0.5 to 11.6) |
| 6H vs 3HR | 3 | 1.3 (0.8 to 2.2) | 0.2 (0 to 7.4) | 1.3 (0.8 to 2.1) |
| 6H vs 3HP | 1 | 0.9 (0.5 to 1.7) | -- | 0.9 (0.5 to 1.7) |
| 6H vs 1HP | 0 | -- | 0.2 (0 to 1.4) | 0.2 (0 to 1.4) |
| 9H vs 12H | 0 | -- | 8.5 (0.8 to 88.8) | 8.5 (0.8 to 88.8) |
| 9H vs 3HR | 0 | -- | 4.6 (0.8 to 27.7) | 4.6 (0.8 to 27.7) |
| 9H vs 3HP | 1 | 3.2 (0.7 to 16) | -- | 3.2 (0.7 to 16) |
| 9H vs 1HP | 1 | 0.8 (0.4 to 1.6) | -- | 0.8 (0.4 to 1.6) |
| 12H vs 3HR | 0 | -- | 0.5 (0.1 to 2.7) | 0.5 (0.1 to 2.7) |
| 12H vs 3HP | 0 | -- | 0.4 (0.1 to 2.1) | 0.4 (0.1 to 2.1) |
| 12H vs 1HP | 0 | -- | 0.1 (0 to 1.1) | 0.1 (0 to 1.1) |
| 3HR vs 3HP | 0 | -- | 0.7 (0.3 to 1.6) | 0.7 (0.3 to 1.6) |
| 3HR vs 1HP | 0 | -- | 0.2 (0 to 1.2) | 0.2 (0 to 1.2) |
| 3HP vs 1HP | 0 | -- | 0.2 (0 to 1.4) | 0.2 (0 to 1.4) |

Notes: IRR, incidence rate ratio; 95%CI, 95% confidence interval; I^2^ for network meta-analysis 0% (0.0% to 44.7%); *Includes studies with 2RZ. Excluded studies: Hawken et al [7], Samandari et al [8], Zar et al [5], Rangaka et al [9], Martinson et al [4], and Madhi et al [3].

### Table O. Effect of TPT regimens on incidence of all-cause mortality, excluding studies with no post-treatment follow up or follow up less than one year.

| **Comparison** | **Number of study arms with direct comparison** | **Direct estimates IRR (95%CI)** | **Indirect estimates IRR (95%CI)** | **Network meta-analysis IRR (95%CI)** |
| --- | --- | --- | --- | --- |
| **Total number of comparisons (studies)*** | 22 (13) |  |  |  |
| 6H vs placebo | 5 | 1.0 (0.8 to 1.1) | 1.9 (0.3 to 13) | 1.0 (0.8 to 1.1) |
| 9H vs placebo | 0 | -- | 0.6 (0.1 to 2.2) | 0.6 (0.1 to 2.2) |
| 12H vs placebo | 2 | 0.9 (0.6 to 1.4) | 0.7 (0.3 to 1.8) | 0.9 (0.6 to 1.3) |
| 36H vs placebo | 0 | -- | 1.0 (0.7 to 1.5) | 1.0 (0.7 to 1.5) |
| 3HR vs placebo | 2 | 0.6 (0.4 to 0.8) | 0.5 (0.2 to 1.2) | 0.6 (0.4 to 0.7) |
| 3HP vs placebo | 0 | -- | 0.6 (0.3 to 1.2) | 0.6 (0.3 to 1.2) |
| 1HP vs placebo | 0 | -- | 0.3 (0.1 to 1.8) | 0.3 (0.1 to 1.8) |
| 6H vs 9H | 0 | -- | 1.7 (0.4 to 6.5) | 1.7 (0.4 to 6.5) |
| 6H vs 12H | 0 | -- | 1.1 (0.7 to 1.8) | 1.1 (0.7 to 1.8) |
| 6H vs 36H | 1 | 0.9 (0.6 to 1.4) | -- | 0.9 (0.6 to 1.4) |
| 6H vs 3HR | 3 | 1.8 (1.3 to 2.4) | 1.5 (0.6 to 3.8) | 1.7 (1.3 to 2.3) |
| 6H vs 3HP | 1 | 1.5 (0.8 to 2.8) | -- | 1.5 (0.8 to 2.8) |
| 6H vs 1HP | 0 | -- | 2.9 (0.5 to 15.3) | 2.9 (0.5 to 15.3) |
| 9H vs 12H | 0 | -- | 0.7 (0.2 to 2.7) | 0.7 (0.2 to 2.7) |
| 9H vs 36H | 0 | -- | 0.6 (0.1 to 2.2) | 0.6 (0.1 to 2.2) |
| 9H vs 3HR | 0 | -- | 1.0 (0.3 to 4) | 1.0 (0.3 to 4) |
| 9H vs 3HP | 1 | 0.9 (0.3 to 2.9) | -- | 0.9 (0.3 to 2.9) |
| 9H vs 1HP | 1 | 1.7 (0.6 to 4.6) | -- | 1.7 (0.6 to 4.6) |
| 12H vs 36H | 0 | -- | 0.8 (0.5 to 1.5) | 0.8 (0.5 to 1.5) |
| 12H vs 3HR | 0 | -- | 1.5 (0.9 to 2.5) | 1.5 (0.9 to 2.5) |
| 12H vs 3HP | 0 | -- | 1.3 (0.6 to 2.9) | 1.3 (0.6 to 2.9) |
| 12H vs 1HP | 0 | -- | 2.5 (0.4 to 14.3) | 2.5 (0.4 to 14.3) |
| 36H vs 3HR | 0 | -- | 1.8 (1.1 to 2.9) | 1.8 (1.1 to 2.9) |
| 36H vs 3HP | 0 | -- | 1.6 (0.8 to 3.3) | 1.6 (0.8 to 3.3) |
| 36H vs 1HP | 0 | -- | 3 (0.5 to 16.9) | 3 (0.5 to 16.9) |
| 3HR vs 3HP | 0 | -- | 0.9 (0.4 to 1.7) | 0.9 (0.4 to 1.7) |
| 3HR vs 1HP | 0 | -- | 1.6 (0.3 to 9) | 1.6 (0.3 to 9) |
| 3HP vs 1HP | 0 | -- | 1.9 (0.4 to 8.9) | 1.9 (0.4 to 8.9) |

Notes: IRR, incidence rate ratio; 95%CI, 95% confidence interval; Person-years of follow up were used as denominator; I^2^ for network meta-analysis 0% (0.0% to 44.5%); *Includes studies with 2RZ. Excluded studies: Madhi et al [3], 72 H arm of Martinson et al [4], Martinez Alfaro et al [6], and Zar et al [5].

### Table P. Effect of TPT regimens on incidence of all-cause mortality, excluding studies with a study setting TB incidence of more than 300 per 100 thousand.

| **Comparisons** | **Number of study arms with direct comparison** | **Direct estimates IRR (95%CI)** | **Indirect estimates IRR (95%CI)** | **Network meta-analysis IRR (95%CI)** |
| --- | --- | --- | --- | --- |
| **Total number of comparisons (studies)*** | 20 (11) |  |  |  |
| 6H vs placebo | 4 | 0.9 (0.8 to 1.1) | 3.6 (0.6 to 22.8) | 0.9 (0.8 to 1.1) |
| 9H vs placebo | 0 | -- | 0.6 (0.1 to 2.1) | 0.6 (0.1 to 2.1) |
| 12H vs placebo | 1 | 1.1 (0.5 to 2.1) | 0.6 (0.3 to 1.2) | 0.8 (0.5 to 1.3) |
| 3HR vs placebo | 2 | 0.6 (0.4 to 0.8) | 0.6 (0.3 to 1.3) | 0.6 (0.4 to 0.8) |
| 3HP vs placebo | 0 | -- | 0.6 (0.3 to 1.2) | 0.6 (0.3 to 1.2) |
| 1HP vs placebo | 0 | -- | 0.3 (0.1 to 1.8) | 0.3 (0.1 to 1.8) |
| 6H vs 9H | 0 | -- | 1.7 (0.4 to 6.5) | 1.7 (0.4 to 6.5) |
| 6H vs 12H | 0 | -- | 1.2 (0.7 to 2) | 1.2 (0.7 to 2) |
| 6H vs 3HR | 3 | 1.8 (1.3 to 2.4) | 1 (0.4 to 2.3) | 1.7 (1.3 to 2.2) |
| 6H vs 3HP | 1 | 1.5 (0.8 to 2.8) | -- | 1.5 (0.8 to 2.8) |
| 6H vs 1HP | 0 | -- | 2.9 (0.5 to 15.3) | 2.9 (0.5 to 15.3) |
| 9H vs 12H | 0 | -- | 0.7 (0.2 to 2.9) | 0.7 (0.2 to 2.9) |
| 9H vs 3HR | 0 | -- | 1 (0.2 to 3.8) | 1 (0.2 to 3.8) |
| 9H vs 3HP | 1 | 0.9 (0.3 to 2.9) | -- | 0.9 (0.3 to 2.9) |
| 9H vs 1HP | 1 | 1.7 (0.6 to 4.6) | -- | 1.7 (0.6 to 4.6) |
| 12H vs 3HR | 1 | 0.7 (0.2 to 2.4) | 1.7 (0.9 to 3.1) | 1.4 (0.8 to 2.4) |
| 12H vs 3HP | 0 | -- | 1.3 (0.6 to 2.9) | 1.3 (0.6 to 2.9) |
| 12H vs 1HP | 0 | -- | 2.4 (0.4 to 14) | 2.4 (0.4 to 14) |
| 3HR vs 3HP | 0 | -- | 0.9 (0.5 to 1.8) | 0.9 (0.5 to 1.8) |
| 3HR vs 1HP | 0 | -- | 1.7 (0.3 to 9.4) | 1.7 (0.3 to 9.4) |
| 3HP vs 1HP | 0 | -- | 1.9 (0.4 to 8.9) | 1.9 (0.4 to 8.9) |

Notes: IRR, incidence rate ratio; 95%CI, 95% confidence interval; Person-years of follow up were used as denominator; I^2^ for network meta-analysis, 0% (0.0% to 58.7%); * Includes studies with 2RZ. Excluded studies: Hawken et al [7], Samandari et al [8], Zar et al [5], Rangaka et al [9], Martinson et al [4], and Madhi et al [3].

### Table Q. Effect of TPT regimens on incidence of all-cause mortality, excluding rifamycin containing studies with a high rate of ART use.

| **Comparison** | **Number of trials with direct comparison** | **Direct estimates IRR (95%CI)** | **Indirect estimates IRR (95%CI)** | **Network meta-analysis IRR (95%CI)** |
| --- | --- | --- | --- | --- |
| **Total number of comparisons (studies)*** | 26 (15) |  |  |  |
| 6 to 12 H vs Placebo | 7 | 1 (0.8 to 1.1) | 1 (0.6 to 1.7) | 1 (0.8 to 1.1) |
| 24 to 72 H vs Placebo | 2 | 1.2 (0.7 to 2) | 0.9 (0.6 to 1.3) | 1 (0.7 to 1.4) |
| 6 to 12 H vs 24 to 72 H | 2 | 1 (0.7 to 1.5) | 0.8 (0.5 to 1.3) | 1 (0.7 to 1.3) |
| 6 to 12 H vs Rifamycin containing | 6 | 1.6 (1.2 to 2.1) | 1.2 (0.5 to 2.7) | 1.6 (1.2 to 2) |
| 24 to 72 H vs Rifamycin containing | 1 | 1.0 (0.4 to 2.3) | 1.8 (1.2 to 2.8) | 1.6 (1.1 to 2.4) |
| Rifamycin containing vs Placebo | 2 | 0.6 (0.4 to 0.8) | 0.7 (0.5 to 1.2) | 0.6 (0.5 to 0.8) |

Notes: IRR, incidence rate ratio; 95%CI, 95% confidence interval; I^2^ for the network meta-analysis 3.5% (0.0% to 52.8%); Placebo includes no treatment; Person-years of follow up were used as denominator; *Includes studies with 2RZ. Rifamycin containing regimens include 3HR, 3HP and 1HP. Excluded studies: Swindells et al [2]. The first regimen mentioned is compared to the second, i.e first regimen over second regimen.

### Table R. Effect of TPT regimens on incidence of microbiologically confirmed and clinically diagnosed TB, stratified by TST/IGRA status.

| **Comparison** | **Number of trials with direct comparison** | **Direct estimates IRR (95%CI)** | **Indirect estimates IRR (95%CI)** | **Network meta-analysis IRR (95%CI)** |
| --- | --- | --- | --- | --- |
| **TST/IGRA positive** | | | | |
| **Total number of direct comparisons (studies)** | 5 (6) |  |  |  |
| 6 to 12H vs Placebo | 3 | 0.7 (0.4 to 1) | 0.8 (0.1 to 8) | 0.7 (0.4 to 1) |
| 24 to 36 H vs Placebo | 1 | 0.2 (0 to 3.8) | 0.2 (0.1 to 0.6) | 0.2 (0.1 to 0.6) |
| Rifamycin containing vs Placebo | 1 | 0.4 (0.2 to 0.8) | 1.1 (0.2 to 7.6) | 0.4 (0.2 to 0.9) |
| **TST/IGRA negative or anergic** | | | | |
| **Total number of direct comparisons (studies)** | 4 (6) |  |  |  |
| 6 to 12H vs Placebo | 3 | 0.7 (0.4 to 1.3) | 0.4 (0.1 to 2.3) | 0.6 (0.4 to 1.1) |
| 24 to 36 H vs Placebo | 1 | 0.5 (0.1 to 1.7) | 0.7 (0.2 to 2.8) | 0.6 (0.2 to 1.5) |
| Rifamycin containing vs Placebo | 0 | -- | 0.3 (0 to 6) | 0.3 (0 to 6) |

Notes: Notes: IRR, incidence rate ratio; 95%CI, 95% confidence interval; I^2^ for network meta-analysis, TST/IGRA positive 0% (0.0%; 77.6%), TST/IGRA negative 50.4% (0.0%; 83.6%); Only the study by Rangaka, et al [9], used TST or IGRA; Rifamycin containing regimens only included 3HR; Included studies: Hawken et al [7], Martinez Alfaro et al [6], Rangaka et al [9], Samandari et al [8], Whalen et al [10], Zar et al [5].

### Table S. Detailed information of completion rates, completion criteria and methods used to assess adherence to the TPT regimes of interest in the included studies.

| First author, year | Completion rates | Completion criteria | Method used to measure compliance |
| --- | --- | --- | --- |
| Gordin, 1997 | 6H: 63.1% (164/260) Placebo: 63.0% (162/257) | Completion of treatment within 12 months | Clinical interviews |
| Martinez Alfaro, 2000 | 3HR: 63% (44/69) 12H: 57% (37/64) | >=80% of the total therapy | Clinical interviews plus pill counts and urine assessment in a selected sample of patients. |
| Rivero, 2007 | 6H: 63.9% (69/108) 3HR: 61.2% (63/103) | >=80% of the planned dosis | Clinical interviews |
| Rivero, 2003 | 6H: 78.3% (65/83) 3HR: 84.1% (69/82) | >=80% of the planned dosis | Clinical interviews |
| Sterling, 2016 | 3HP: 89 (183/206) 9H: 64% (123/193) | 3HP: 11 to 12 doses within 16 weeks 9H: 240 doses within 52 weeks | Determined by direct observed therapy records (3HP), pill count and interview (9H). |
| Temprano group, 2015 | 6H + deferred ART: 93% (431/461)**** 6H + early ART: 94% (437/466)**** | Attended all six isoniazid prescription visits | Prescription visits |
| Badje, 2017 (Temprano follow-up) | See Temprano group, 2015 | See Temprano group, 2015 | See Temprano group, 2015 |
| Swindells, 2019 | 1HP: 97.0% (1451/1496) 9H: 90.0% (1353/1504) | Receiving >=90% doses within the following periods: 1HP: complete within 8 weeks  9H: complete within 54 weeks | Pill counts and self-report adherence interviews were used to assess adherence during the treatment phase of each arm. |
| Fitzgerald, 2001 | No information | No information | No information |
| Whalen, 1997 | Placebo TST+: 90.1% (418/464) Placebo TST-: 86.1% (278/323) 6H TST+: 91.6% (491/536) 6H TST -: 84.6% (334/395) 3HR TST+: 92.8% (516/556) | Not available | Assessed using attendance to visits, measuring isoniazid metabolites in urine in a random sample, and self-reports. |
| Johnson, 2001 | See Whalen, 1997 | Not available | See Whalen, 1997 |
| Hawken, 1997 | 6H: 31.5% (107/342) Placebo: 30.8% (103/342) | Not defined. On the left is the number of subjects missing 5 or more weeks of therapy. | Tablet counts, estimation of the number of weeks missed in 6-months, and urine testing for isocotinic acid in a random sample |
| Samandari, 2011* | 6H: 78% (771/989) 36H: 77% (775/1006) | Attending >=80% of visits within six 6-month periods. | Refill visits plus a isoniazid urine testing in a random sample. |
| Samandari, 2015 | See Samandari, 2011. | See Samandari, 2011. | See Samandari, 2011. |
| Zar, 2006 | No information | No information | No information |
| Rangaka, 2014 | Placebo: 82.5% (550/667) 12H: 83.1% (550/662) | Completion of 12 months of the study drug (could be completed within 15 months) | Adherence was monitored through pharmacy refill records. |
| Martinson, et al 2011 | 3HP: 95.7% (314/328) 6H: 83.8% (274/327) 72H: 43.3% (71/164)** | Taking >=90% of their medication in the following time:  3HP: 12 doses within 24 weeks 6H: 12 months to complete. 72H: continuous dosing. | Calculating the percentage of doses taken using pill counts and observation of medication. |
| Madhi, 2011 | 24H: 27.4% (75/274)*** Placebo: 29.6% (81/274)*** | Not available | Standardized interviews. |
| Gordin, 2000 | 12H: 69% (544/792) | Not specified | Self administration regime (no information available regarding adherence assessment) |
|  | | | |
|  | | | |

Abbreviatures: H, isoniazid; P, Rifapentine; R, rifampin; Z, pyrazinamide.

*Reported in [11]

** > 4 years of therapy

*** 130 in the 24H arm and 141 in the placebo arm did not complete the intervention because of study closure

**** Calculated over the number of subjects that ever started ART

### Table T. Effect of aggregated TPT regimens on incidence of all-cause mortality, stratified by the proportion of study participants receiving ART (above, or below 50%).

| Comparison | Number of study arms with direct comparisons | Direct estimates  IRR (95%CI) | Indirect estimates  IRR (95%CI) | Network meta-analysis IRR (95%CI) |  |
| --- | --- | --- | --- | --- | --- |
| More than 50 % of participants received ART (ever) | | | | | |
| Total number of comparisons (studies) | 5 (5) |  |  |  |  |
| 6 to 12 H vs Placebo | 3 | 0.8 (0.6 to 1.1) | -- | 0.8 (0.6 to 1.1) |  |
| 24 to 72 H vs placebo | 1 | 1.8 (0.9 to 3.6) | -- | 1.8 (0.9 to 3.6) |  |
| 6 to 12 H vs 24 to 72 H | 0 | -- | 0.5 (0.2 to 1) | 0.5 (0.2 to 1) |  |
| 6 to 12 H vs Rifamycin containing | 1 | 1.7 (0.6 to 4.9) | -- | 1.7 (0.6 to 4.9) |  |
| 24 to 72 H vs Rifamycin containing | 0 | -- | 3.7 (1 to 13.6) | 3.7 (1 to 13.6) |  |
| Rifamycin containing vs Placebo | 0 | -- | 0.5 (0.2 to 1.5) | 0.5 (0.2 to 1.5) |  |
| Less than 50 % of participants received ART (ever)* | | | | | |
| Total number of comparisons (studies) | 22 (11) |  |  |  |  |
| 6 to 12 H vs Placebo | 4 | 1 (0.9 to 1.2) | 0.5 (0.3 to 1) | 1 (0.8 to 1.2) |  |
| 24 to 72 H vs placebo | 1 | 0.5 (0.2 to 1.3) | 1 (0.7 to 1.4) | 0.9 (0.6 to 1.2) |  |
| 6 to 12 H vs 24 to 72 H | 2 | 1 (0.7 to 1.5) | 1.9 (0.8 to 4.7) | 1.1 (0.8 to 1.5) |  |
| 6 to 12 H vs Rifamycin containing | 6 | 1.6 (1.2 to 2.1) | 1.3 (0.4 to 3.8) | 1.6 (1.2 to 2) |  |
| 24 to 72 H vs Rifamycin containing | 1 | 1 (0.4 to 2.3) | 1.6 (1 to 2.4) | 1.4 (1 to 2.1) |  |
| Rifamycin containing vs Placebo | 2 | 0.6 (0.4 to 0.8) | 0.8 (0.5 to 1.5) | 0.6 (0.5 to 0.8) |  |

* For studies that did not report ART use we assumed that participants did not receive ART if recruitment finished before the year 2000. Four studies from 3 trials fulfilled this definition: Rivero et al. 2003 and 2007 (Spain, finished recruitment on 1998) [12,13], Johnson and Whalen et al. (Uganda, finished recruitment in 1995)[10,14] , and Hawken et al. (Kenya, finished recruitment in 1994) [7].

### Table U. Effect of aggregated TPT regimens on incidence of microbiologically confirmed TB, stratified by the proportion of study participants receiving ART (above, or below 50%).

| Comparison | Number of study arms with direct comparison | Direct estimates IRR (95%CI) | Indirect estimates IRR (95%CI) | Network meta-analysis IRR (95%CI) |  |
| --- | --- | --- | --- | --- | --- |
| More than 50 % of study participants received ART (ever) | | | | | |
| Total number of comparisons (studies)* | 4 (4) |  |  |  |  |
| 6 to 12 H vs Placebo | 2 | 0.5 (0.3 to 1) | -- | 0.5 (0.3 to 1) |  |
| 24 to 72 H vs placebo | 1 | 1 (0.3 to 3.1) | -- | 1 (0.3 to 3.1) |  |
| 6 to 12 H vs 24 to 72 H | 0 | -- | 0.5 (0.1 to 2.1) | 0.5 (0.1 to 2.1) |  |
| 6 to 12 H vs Rifamycin containing | 1 | 0.8 (0.4 to 1.6) | -- | 0.8 (0.4 to 1.6) |  |
| 24 to 72 H vs Rifamycin containing | 0 | -- | 1.4 (0.3 to 6.6) | 1.4 (0.3 to 6.6) |  |
| Rifamycin containing vs Placebo | 0 | -- | 0.7 (0.3 to 1.7) | 0.7 (0.3 to 1.7) |  |
| Less than 50 % of study participants received ART (ever)* | | | | | |
| Total number of comparisons (studies) | 18 (9) |  |  |  |  |
| 6 to 12 H vs Placebo | 2 | 0.9 (0.5 to 1.6) | 0.6 (0.1 to 3.4) | 0.9 (0.5 to 1.5) |  |
| 24 to 72 H vs placebo | 1 | 0.2 (0 to 3) | 0.6 (0.3 to 1.1) | 1 (0.3 to 3.1) |  |
| 6 to 12 H vs 24 to 72 H | 2 | 1.6 (1 to 2.5) | 7.7 (0.8 to 73.8) | 1.7 (1.1 to 2.6) |  |
| 6 to 12 H vs Rifamycin containing | 5 | 1.2 (0.8 to 1.8) | 0.6 (0.1 to 2.4) | 0.8 (0.4 to 1.6) |  |
| 24 to 72 H vs Rifamycin containing | 1 | 0.5 (0.2 to 1.3) | 0.8 (0.4 to 1.5) | 1.4 (0.3 to 6.6) |  |
| Rifamycin containing vs Placebo | 2 | 1 (0.2 to 4.4) | 0.7 (0.4 to 1.4) | 0.7 (0.3 to 1.7) |  |

* For studies that did not report ART use we assumed that participants did not receive ART if recruitment finished before the year 2000. Four studies from 3 trials fulfilled this definition: Rivero et al. 2003 and 2007 (Spain, finished recruitment on 1998) [12,13], Johnson and Whalen et al. (Uganda, finished recruitment in 1995) [10,14], and Hawken et al. (Kenya, finished recruitment in 1994) [7].

### Table V. Network meta-analysis of incidence of all-cause mortality throughout study duration, by aggregated TPT regimens and adjusted for the proportion of subjects receiving anti-retroviral therapy.

| Comparison | Number of trials with direct comparisons | Direct estimates  IRR (95%CI) | Indirect estimates  IRR (95%CI) | Network meta-analysis  IRR (95%CI) | Adjusted for ART use*  IRR |
| --- | --- | --- | --- | --- | --- |
| Total number of comparisons (studies)* | 27 (16) |  |  |  |  |
| 6 to 12 H vs Placebo | 7 | 1.0 (0.8 to 1.1) | 1.0 (0.6 to 1.6) | 1.0 (0.8 to 1.1) | 1.0 |
| 24 to 72 H vs Placebo | 2 | 1.2 (0.7 to 2.0) | 0.9 (0.6 to 1.3) | 1.0 (0.7 to 1.4) | 1.0 |
| 6 to 12 H vs 24 to 72 H | 2 | 1.0 (0.7 to 1.5) | 0.8 (0.5 to 1.3) | 1.0 (0.7 to 1.3) | 1.0 |
| 6 to 12 H vs Rifamycin containing | 7 | 1.6 (1.3 to 2.1) | 1.2 (0.5 to 2.7) | 1.6 (1.2 to 2.0) | 1.6 |
| 24 to 72 H vs Rifamycin containing | 1 | 1.0 (0.4 to 2.3) | 1.8 (1.2 to 2.7) | 1.6 (1.2 to 2.4) | 1.6 |
| Rifamycin containing vs Placebo | 2 | 0.6 (0.4 to 0.8) | 0.7 (0.5 to 1.1) | 0.6 (0.5 to 0.8) | 0.6 |

*Adjusted for the proportion of subjects receiving ART in each study, using meta-regression for Network meta-analysis with methods suggested by Lumley, et al [15].

### Table W. Network meta-analysis of incidence of microbiologically confirmed active TB throughout study duration, by aggregated TPT regimens and adjusted for the proportion of subjects receiving anti-retroviral therapy.

| Comparison | Number of trials with direct comparison | Direct estimates  IRR (95%CI) | Indirect estimates  IRR (95%CI) | Network meta-analysis  IRR (95%CI) | Adjusted for ART use*  IRR |
| --- | --- | --- | --- | --- | --- |
| Total number of comparisons (studies)* | 22 (13) |  |  |  |  |
| 6 to 12 H vs Placebo | 4 | 0.7 (0.5 to 1.1) | 1.1 (0.4 to 3.1) | 0.7 (0.5 to 1.1) | 0.8 |
| 24 to 72 H vs Placebo | 2 | 0.7 (0.2 to 2.2) | 0.4 (0.2 to 0.8) | 0.5 (0.3 to 0.8) | 0.5 |
| 6 to 12 H vs 24 to 72 H | 2 | 1.6 (1 to 2.5) | 1.2 (0.4 to 3.5) | 1.5 (1.0 to 2.3) | 1.6 |
| 6 to 12 H vs Rifamycin containing | 6 | 1.1 (0.8 to 1.5) | 0.5 (0.1 to 2.0) | 1.0 (0.8 to 1.4) | 1.1 |
| 24 to 72 H vs Rifamycin containing | 1 | 0.5 (0.2 to 1.3) | 0.8 (0.4 to 1.4) | 0.7 (0.4 to 1.2) | 0.7 |
| Rifamycin containing vs Placebo | 1 | 1 (0.2 to 4.4) | 0.7 (0.4 to 1.2) | 0.7 (0.4 to 1.2) | 0.8 |

* Adjusted for proportion of subjects receiving ART in each study, using meta-regression for Network meta-analysis using the methods suggested by Lumley, et al [15].

### Table X. Network meta-analysis of all-cause mortality by individual TPT regimens adjusted for ever use of anti-retroviral therapy.

| Comparison | Number of study arms with direct comparison | Direct estimates IRR (95%CI) | Indirect estimates IRR (95%CI) | Network meta-analysis IRR (95%CI) | Adjusted for ART use*  IRR |
| --- | --- | --- | --- | --- | --- |
| 6H vs placebo | 5 | 1.0 (0.8 to 1.1) | 2.7 (0.5 to 14.8) | 1.0 (0.8 to 1.1) | 1.00 |
| 9H vs placebo | 0 | -- | 0.6 (0.1 to 2.2) | 0.6 (0.1 to 2.2) | 0.6 |
| 12H vs placebo | 2 | 0.9 (0.5 to 1.5) | 0.6 (0.3 to 1.2) | 0.8 (0.5 to 1.2) | 0.8 |
| 24H vs placebo | 2 | 1.2 (0.7 to 2.1) | -- | 1.2 (0.7 to 2.1) | 1.3 |
| 36H vs placebo | 0 | -- | 1.0 (0.6 to 1.6) | 1.0 (0.6 to 1.6) | 1.0 |
| 72H vs placebo | 0 | -- | 0.6 (0.3 to 1.5) | 0.6 (0.3 to 1.5) | 0.7 |
| 3HR vs placebo | 2 | 0.6 (0.4 to 0.8) | 0.6 (0.3 to 1.4) | 0.6 (0.4 to 0.8) | 0.6 |
| 3HP vs placebo | 0 | -- | 0.6 (0.3 to 1.2) | 0.6 (0.3 to 1.2) | 0.7 |
| 1HP vs placebo | 0 | -- | 0.3 (0.1 to 1.9) | 0.3 (0.1 to 1.9) | 0.3 |
| 6H vs 9H | 0 | -- | 1.7 (0.4 to 6.6) | 1.7 (0.4 to 6.6) | 1.7 |
| 6H vs 12H | 0 | -- | 1.2 (0.8 to 1.9) | 1.2 (0.8 to 1.9) | 1.2 |
| 6H vs 24H | 0 | -- | 0.8 (0.5 to 1.4) | 0.8 (0.5 to 1.4) | 0.8 |
| 6H vs 36H | 1 | 0.9 (0.6 to 1.4) | -- | 0.9 (0.6 to 1.4) | 1.0 |
| 6H vs 72H | 1 | 1.5 (0.7 to 3.4) | -- | 1.5 (0.7 to 3.4) | 1.5 |
| 6H vs 3HR | 3 | 1.8 (1.3 to 2.5) | 1.2 (0.5 to 2.7) | 1.7 (1.2 to 2.3) | 1.7 |
| 6H vs 3HP | 1 | 1.5 (0.8 to 2.9) | -- | 1.5 (0.8 to 2.9) | 1.5 |
| 6H vs 1HP | 0 | -- | 92. (0.5 to 15.7) | 2.9 (0.5 to 15.7) | 3.1 |
| 9H vs 12H | 0 | -- | 0.7 (0.2 to 3) | 0.7 (0.2 to 3) | 0.7 |
| 9H vs 24H | 0 | -- | 0.5 (0.1 to 2) | 0.5 (0.1 to 2) | 0.5 |
| 9H vs 36H | 0 | -- | 0.6 (0.1 to 2.3) | 0.6 (0.1 to 2.3) | 0.6 |
| 9H vs 72H | 0 | -- | 0.9 (0.2 to 3.9) | 0.9 (0.2 to 3.9) | 0.9 |
| 9H vs 3HR | 0 | -- | 1.0 (0.2 to 4) | 1.0 (0.2 to 4) | 1.0 |
| 9H vs 3HP | 1 | 0.9 (0.3 to 3) | -- | 0.9 (0.3 to 3) | 0.9 |
| 9H vs 1HP | 1 | 1.7 (0.6 to 4.7) | -- | 1.7 (0.6 to 4.7) | 1.9 |
| 12H vs 24H | 0 | -- | 0.6 (0.3 to 1.3) | 0.6 (0.3 to 1.3) | 0.6 |
| 12H vs 36H | 0 | -- | 0.8 (0.4 to 1.4) | 0.8 (0.4 to 1.4) | 0.8 |
| 12H vs 72H | 0 | -- | 1.2 (0.5 to 3.1) | 1.2 (0.5 to 3.1) | 1.2 |
| 12H vs 3HR | 1 | 0.7 (0.2 to 2.4) | 1.5 (0.9 to 2.6) | 1.4 (0.8 to 2.2) | 1.4 |
| 12H vs 3HP | 0 | -- | 1.2 (0.6 to 2.7) | 1.2 (0.6 to 2.7) | 1.2 |
| 12H vs 1HP | 0 | -- | 2.3 (0.4 to 13.5) | 2.3 (0.4 to 13.5) | 2.5 |
| 24H vs 36H | 0 | -- | 1.2 (0.6 to 2.4) | 1.2 (0.6 to 2.4) | 1.2 |
| 24H vs 72H | 0 | -- | 1.9 (0.7 to 5.1) | 1.9 (0.7 to 5.1) | 2.0 |
| 24H vs 3HR | 0 | -- | 2.1 (1.1 to 3.9) | 2.1 (1.1 to 3.9) | 2.3 |
| 24H vs 3HP | 0 | -- | 1.9 (0.8 to 4.5) | 1.9 (0.8 to 4.5) | 2 |
| 24H vs 1HP | 0 | -- | 3.6 (0.6 to 21.6) | 3.6 (0.6 to 21.6) | 4.1 |
| 36H vs 72H | 0 | -- | 1.6 (0.6 to 4) | 1.6 (0.6 to 4) | 1.5 |
| 36H vs 3HR | 0 | -- | 1.8 (1 to 3) | 1.8 (1 to 3) | 1.8 |
| 36H vs 3HP | 0 | -- | 1.6 (0.7 to 3.5) | 1.6 (0.7 to 3.5) | 1.5 |
| 36H vs 1HP | 0 | -- | 3 (0.5 to 17.5) | 3 (0.5 to 17.5) | 3.1 |
| 72H vs 3HR | 0 | -- | 1.1 (0.5 to 2.6) | 1.1 (0.5 to 2.6) | 1.1 |
| 72H vs 3HP | 1 | 1.0 (0.4 to 2.4) | -- | 1.0 (0.4 to 2.4) | 1.0 |
| 72H vs 1HP | 0 | -- | 1.9 (0.3 to 11.4) | 1.9 (0.3 to 11.4) | 2.0 |
| 3HR vs 3HP | 0 | -- | 0.9 (0.4 to 1.9) | 0.9 (0.4 to 1.9) | 0.9 |
| 3HR vs 1HP | 0 | -- | 1.7 (0.3 to 9.7) | 1.7 (0.3 to 9.7) | 1.8 |
| 3HP vs 1HP | 0 | -- | 1.9 (0.4 to 9.1) | 1.9 (0.4 to 9.1) | 2.0 |

* Adjusted for proportion of subjects receiving ART in each study, using meta-regression for Network meta-analysis using with the methods suggested by Lumley, et al [15].

### Table Y. Network meta-analysis of microbiologically confirmed TB by individual TPT regimens, adjusted for ever use of anti-retroviral therapy.

| Comparison | Number of study arms with direct comparison | Direct estimates IRR (95%CI) | Indirect estimates IRR (95%CI) | Network meta-analysis  IRR (95%CI) | Adjusted for ART use*  IRR |
| --- | --- | --- | --- | --- | --- |
| 6H vs placebo | 3 | 0.8 (0.5 to 1.4) | 1.5 (0.3 to 8) | 0.9 (0.5 to 1.4) | 0.9 |
| 9H vs placebo | 0 | -- | 3.1 (0.5 to 18.8) | 3.1 (0.5 to 18.8) | 3.3 |
| 12H vs placebo | 1 | 0.5 (0.3 to 1.1) | 0.4 (0.1 to 1.8) | 0.5 (0.3 to 1.0) | 0.5 |
| 24H vs placebo | 2 | 0.7 (0.2 to 2.2) | -- | 0.7 (0.2 to 2.2) | 0.7 |
| 36H vs placebo | 0 | -- | 0.6 (0.3 to 1.2) | 0.6 (0.3 to 1.2) | 0.6 |
| 72H vs placebo | 0 | -- | 0.5 (0.2 to 1.5) | 0.5 (0.2 to 1.5) | 0.5 |
| 3HR vs placebo | 1 | 1.0 (0.2 to 4.4) | 0.6 (0.3 to 1.3) | 0.7 (0.4 to 1.3) | 0.7 |
| 3HP vs placebo | 0 | -- | 1.0 (0.4 to 2.2) | 1.0 (0.4 to 2.2) | 1 |
| 1HP vs placebo | 0 | -- | 4 (0.6 to 27.5) | 4.0 (0.6 to 27.5) | 4.5 |
| 6H vs 9H | 0 | -- | 0.3 (0 to 1.5) | 0.3 (0 to 1.5) | 0.3 |
| 6H vs 12H | 0 | -- | 1.7 (0.8 to 3.8) | 1.7 (0.8 to 3.8) | 1.9 |
| 6H vs 24H | 0 | -- | 1.2 (0.4 to 3.9) | 1.2 (0.4 to 3.9) | 1.3 |
| 6H vs 36H | 1 | 1.5 (0.9 to 2.6) | -- | 1.5 (0.9 to 2.6) | 1.5 |
| 6H vs 72H | 1 | 1.8 (0.7 to 4.8) | -- | 1.8 (0.7 to 4.8) | 1.8 |
| 6H vs 3HR | 3 | 1.3 (0.8 to 2.2) | 0.5 (0 to 5.9) | 1.3 (0.8 to 2.1) | 1.2 |
| 6H vs 3HP | 1 | 0.9 (0.5 to 1.7) | -- | 0.9 (0.5 to 1.7) | 0.9 |
| 6H vs 1HP | 0 | -- | 0.2 (0 to 1.4) | 0.2 (0 to 1.4) | 0.2 |
| 9H vs 12H | 0 | -- | 6.3 (0.9 to 41.4) | 6.3 (0.9 to 41.4) | 7.1 |
| 9H vs 24H | 0 | -- | 4.3 (0.5 to 34.7) | 4.3 (0.5 to 34.7) | 4.8 |
| 9H vs 36H | 0 | -- | 5.5 (0.9 to 33.4) | 5.5 (0.9 to 33.4) | 5.5 |
| 9H vs 72H | 0 | -- | 6.4 (1.0 to 41.7) | 6.4 (1.0 to 41.7) | 6.6 |
| 9H vs 3HR | 0 | -- | 4.6 (0.8 to 27.6) | 4.6 (0.8 to 27.6) | 4.6 |
| 9H vs 3HP | 1 | 3.2 (0.7 to 16) | -- | 3.2 (0.7 to 16) | 3.3 |
| 9H vs 1HP | 1 | 0.8 (0.4 to 1.6) | -- | 0.8 (0.4 to 1.6) | 0.7 |
| 12H vs 24H | 0 | -- | 0.7 (0.2 to 2.4) | 0.7 (0.2 to 2.4) | 0.7 |
| 12H vs 36H | 0 | -- | 0.9 (0.3 to 2.2) | 0.9 (0.3 to 2.2) | 0.8 |
| 12H vs 72H | 0 | -- | 1 (0.3 to 3.6) | 1.0 (0.3 to 3.6) | 0.9 |
| 12H vs 3HR | 0 | -- | 0.7 (0.3 to 1.8) | 0.7 (0.3 to 1.8) | 0.7 |
| 12H vs 3HP | 0 | -- | 0.5 (0.2 to 1.4) | 0.5 (0.2 to 1.4) | 0.5 |
| 12H vs 1HP | 0 | -- | 0.1 (0 to 0.9) | 0.1 (0 to 0.9) | 0.1 |
| 24H vs 36H | 0 | -- | 1.3 (0.4 to 4.8) | 1.3 (0.4 to 4.8) | 1.2 |
| 24H vs 72H | 0 | -- | 1.5 (0.3 to 7.2) | 1.5 (0.3 to 7.2) | 1.4 |
| 24H vs 3HR | 0 | -- | 1.1 (0.3 to 3.9) | 1.1 (0.3 to 3.9) | 1 |
| 24H vs 3HP | 0 | -- | 0.8 (0.2 to 3) | 0.8 (0.2 to 3) | 0.7 |
| 24H vs 1HP | 0 | -- | 0.2 (0 to 1.7) | 0.2 (0 to 1.7) | 0.2 |
| 36H vs 72H | 0 | -- | 1.2 (0.4 to 3.5) | 1.2 (0.4 to 3.5) | 1.2 |
| 36H vs 3HR | 0 | -- | 0.8 (0.4 to 1.7) | 0.8 (0.4 to 1.7) | 0.8 |
| 36H vs 3HP | 0 | -- | 0.6 (0.3 to 1.3) | 0.6 (0.3 to 1.3) | 0.6 |
| 36H vs 1HP | 0 | -- | 0.1 (0 to 1.0) | 0.1 (0 to 1.0) | 0.1 |
| 72H vs 3HR | 0 | -- | 0.7 (0.2 to 2.2) | 0.7 (0.2 to 2.2) | 0.7 |
| 72H vs 3HP | 1 | 0.5 (0.2 to 1.3) | -- | 0.5 (0.2 to 1.3) | 0.5 |
| 72H vs 1HP | 0 | -- | 0.1 (0 to 0.9) | 0.1 (0 to 0.9) | 0.1 |
| 3HR vs 3HP | 0 | -- | 0.7 (0.3 to 1.6) | 0.7(0.3 to 1.6) | 0.7 |
| 3HR vs 1HP | 0 | -- | 0.2 (0 to 1.2) | 0.2 (0 to 1.2) | 0.2 |
| 3HP vs 1HP | 0 | -- | 0.2 (0 to 1.4) | 0.2 (0 to 1.4) | 0.2 |

* Adjusted for proportion of subjects receiving ART in each study, using meta-regression for Network meta-analysis using the methods suggested by Lumley, et al [15].

References

1. Danel C, Moh R, Gabillard D, Badje A, Le Carrou J, Ouassa T, et al. A Trial of Early Antiretrovirals and Isoniazid Preventive Therapy in Africa. N Engl J Med. 2015;373(9):808-22. Epub 2015/07/21. doi: 10.1056/NEJMoa1507198. PubMed PMID: 26193126.
2. Swindells S, Ramchandani R, Gupta A, Benson CA, Leon-Cruz J, Mwelase N, et al. One Month of Rifapentine plus Isoniazid to Prevent HIV-Related Tuberculosis. N Engl J Med. 2019;380(11):1001-11. doi: 10.1056/NEJMoa1806808. PubMed PMID: 30865794.
3. Madhi SA, Nachman S, Violari A, Kim S, Cotton MF, Bobat R, et al. Primary isoniazid prophylaxis against tuberculosis in HIV-exposed children. N Engl J Med. 2011;365(1):21-31. Epub 2011/07/08. doi: 10.1056/NEJMoa1011214. PubMed PMID: 21732834; PubMed Central PMCID: PMCPMC3164539.
4. Martinson NA, Barnes GL, Moulton LH, Msandiwa R, Hausler H, Ram M, et al. New regimens to prevent tuberculosis in adults with HIV infection. N Engl J Med. 2011;365(1):11-20. Epub 2011/07/08. doi: 10.1056/NEJMoa1005136. PubMed PMID: 21732833; PubMed Central PMCID: PMCPMC3407678.
5. Zar HJ, Cotton MF, Strauss S, Karpakis J, Hussey G, Schaaf HS, et al. Effect of isoniazid prophylaxis on mortality and incidence of tuberculosis in children with HIV: randomised controlled trial. BMJ. 2007;334(7585):136. Epub 2006/11/07. doi: 10.1136/bmj.39000.486400.55. PubMed PMID: 17085459; PubMed Central PMCID: PMCPMC1779846.
6. Martínez Alfaro EM, Cuadra F, Solera J, Maciá MA, Geijo P, Sánchez Martínez PA, et al. [Evaluation of 2 tuberculosis chemoprophylaxis regimens in patients infected with human immunodeficiency virus. The GECMEI Group]. Medicina clinica. 2000;115(5):161-5. Epub 2000/09/21. doi: 10.1016/s0025-7753(00)71496-5. PubMed PMID: 10996870.
7. Hawken MP, Meme HK, Elliott LC, Chakaya JM, Morris JS, Githui WA, et al. Isoniazid preventive therapy for tuberculosis in HIV-1-infected adults: results of a randomized controlled trial. AIDS. 1997;11(7):875-82. Epub 1997/06/01. doi: 10.1097/00002030-199707000-00006. PubMed PMID: 9189212.
8. Samandari T, Agizew TB, Nyirenda S, Tedla Z, Sibanda T, Shang N, et al. 6-month versus 36-month isoniazid preventive treatment for tuberculosis in adults with HIV infection in Botswana: a randomised, double-blind, placebo-controlled trial. Lancet. 2011;377(9777):1588-98. Epub 2011/04/16. doi: 10.1016/s0140-6736(11)60204-3. PubMed PMID: 21492926.
9. Rangaka MX, Wilkinson RJ, Boulle A, Glynn JR, Fielding K, van Cutsem G, et al. Isoniazid plus antiretroviral therapy to prevent tuberculosis: a randomised double-blind, placebo-controlled trial. Lancet. 2014;384(9944):682-90. Epub 2014/05/20. doi: 10.1016/s0140-6736(14)60162-8. PubMed PMID: 24835842; PubMed Central PMCID: PMCPMC4233253.
10. Whalen CC, Johnson JL, Okwera A, Hom DL, Huebner R, Mugyenyi P, et al. A trial of three regimens to prevent tuberculosis in Ugandan adults infected with the human immunodeficiency virus. Uganda-Case Western Reserve University Research Collaboration. N Engl J Med. 1997;337(12):801-8. Epub 1997/09/19. doi: 10.1056/nejm199709183371201. PubMed PMID: 9295239.
11. Gust DA, Mosimaneotsile B, Mathebula U, Chingapane B, Gaul Z, et al. (2011) Risk Factors for Non-Adherence and Loss to Follow-Up in a Three-Year Clinical Trial in Botswana. PLOS ONE 6(4): e18435. https://doi.org/10.1371/journal.pone.0018435
12. Rivero A, López-Cortés L, Castillo R, Verdejo J, García MA, Martínez-Marcos FJ, et al. [Randomized clinical trial investigating three chemoprophylaxis regimens for latent tuberculosis infection in HIV-infected patients]. Enferm Infecc Microbiol Clin. 2007;25(5):305-10. Epub 2007/05/17. doi: 10.1157/13102265. PubMed PMID: 17504683.
13. Rivero A, López-Cortés L, Castillo R, Lozano F, García MA, Díez F, et al. [Randomized trial of three regimens to prevent tuberculosis in HIV-infected patients with anergy]. Enferm Infecc Microbiol Clin. 2003;21(6):287-92. Epub 2003/06/18. doi: 10.1016/s0213-005x(03)72942-5. PubMed PMID: 12809582.
14. Johnson JL, Okwera A, Hom DL, Mayanja H, Mutuluuza Kityo C, Nsubuga P, et al. Duration of efficacy of treatment of latent tuberculosis infection in HIV-infected adults. AIDS. 2001;15(16):2137-47. Epub 2001/10/31. doi: 10.1097/00002030-200111090-00009. PubMed PMID: 11684933.
15. Lumley T. Network meta-analysis for indirect treatment comparisons. Stat Med. 2002; 21(16):2313-24. doi: 10.1002/sim.1201. PMID: 12210616.
