# Supplementary material for: Tuberculosis preventive therapy for people living with HIV: A systematic review and network meta-analysis
Source: PLoS Med. 2021 Sep 14;18(9):e1003738. doi: 10.1371/journal.pmed.1003738 (PMC8439495; doi:10.1371/journal.pmed.1003738)
Supplement: Attachment — Submitted filename: yaneslane.pdf [file pmed.1003738.s003.pdf]

This paper describes a systematic review with meta–analysis of studies of preventive therapy for TB in people living with HIV.

I was asked for a statistical report and I interpret that to include all aspects of the design and conduct of the study.

I see that this is one of three linked papers and I have previously reviewed another and a third is living in my pending tray. I have reviewed them separately so some of the comments may appear for more than one of them.

Points of detail

Page 3 I think this is the first occurrence of H as an abbreviation. I did manage to work out what it means and it is explained on page 11. You TB folk certainly have a way with abbreviations, H = isoniazid?

Page 4 I think excluding observational studies hardly needs justifying if there are enough trials.

Page 4 Various empirical studies have indicated that the main bibliographic databases have varied and incomplete coverage of material not in English (Pilkington et al., 2005), and have limited coverage of material from low and middle–income country journals (Kieling et al., 2009; Syed Sheriff et al., 2008). I am not a TB or HIV person but I understand they both have a major burden in LMIC so extending the search into one of other of the WHO sponsored databases might be helpful. The current search has brought enough studies to light but not so many that a few more would not be welcome.

Page 4 Why exclude studies with fewer than 25 per arm? I agree they will be less precise but the analysis should take care of that.

Page 5 Perhaps for completeness say how the articles in languages other than English were dealt with. There does seem to have been at least one (reference 27).

Page 7 The only problem with not providing a ranking of treatments is that the reader is going to impose their own ranking on them anyway so it might be better to present something and stress the uncertainty to protect the reader from over–exuberant extrapolation.

Page 8 Providing the software versions is seldom done but helps reproducibility.

Page 9 The full list of excluded studies is a valuable addition.

Page 13, Table 2 I do not grasp what the sentence ‘For the study by Martinson et al, 3HP and 3HR arms were grouped together, for all other studies treatment arms remained the same.’ means. Specifically what does it mean for an arm to remain the same?

Page 15, Table 3 I think I have missed something here as the 40% figure in the caption does not seem to be reflective of anything in the body of the table.

References The authors’ bibliographic management software seems to have had problems with references 24 and 25 and maybe some others, I did not do an exhaustive check.

Figure 1 The caption seems to duplicate information which is in the box for exclusions. A new abbreviation for isoniazid is introduced here too.

Supplementary heatmaps Two of these did not come through properly although that often seems to happen to me with figures in Word documents perhaps because I use LibreOffice. I assume they will be OK in the final production version. Figure S5 did come through although was not that informative.

I think there are some advantages in using what `netmeta` calls network league tables for Tables 2, 3, 4 and equivalent in the supplement. A publication quality example can be found in Barth et al. (2013, Table 3). The advantage is that all comparisons involving a particular condition are adjacent. In table 2 for instance the 6 to 12 H comparisons are not adjacent nor are the 24 to 72 H.

Point of more substance

There are a number of possible diagnostics which can be run after the main analysis to check for things like inconsistency. If the authors feel, for some reason, that these are inappropriate then an explanation of why would be in order.

Summary

Mostly comments and suggestions for more detail and analysis.

Michael Dewey

References

J Barth, T Munder, H Gerger, E Nüesch, S Trelle, H Znoj, P Jüni, and P Cuijpers. Comparative efficacy of seven psychotherapeutic interventions for patients with depression: a network meta–analysis. *PLOS Medicine*, 10(5), 2013.

C Kieling, H Herrman, V Patel, and J J Mari. Indexation of psychiatric journals from low– and middle–income countries: a survey and a case study. *World Psychiatry*, 8:40–44, 2009.

K Pilkington, A Boshnakova, M Clarke, and J Richardson. 'No language restrictions' in database searches: what does this really mean? *The Journal of Alternative and Complementary Medicine*, 11:205–207, 2005.

R J Syed Sheriff, C E Adams, P Tharyan, M Jayaram, L Duley, and the PRACTIHC Mental Health Group. Randomised trials relevant to mental health conducted in low and middle–income countries: a survey. *BMC Psychiatry*, 8(69), 2008.
