# Supplementary material for: Tuberculosis preventive therapy for people living with HIV: A systematic review and network meta-analysis
Source: PLoS Med. 2021 Sep 14;18(9):e1003738. doi: 10.1371/journal.pmed.1003738 (PMC8439495; doi:10.1371/journal.pmed.1003738)
Supplement: Attachment — Submitted filename: NMAofTPTinPLHIV ResponseToReviewers R1.docx [file pmed.1003738.s004.docx]

Montreal, 31 March 2021

*REF: “"Tuberculosis Preventive Therapy for People Living with HIV: A Systematic Review and Network Meta-analysis" (PMEDICINE-D-20-06010R1)”*

Dr Raffaella Bosurgi,

Executive Editor

Medicine

PLOS Medicine

plosmedicine.org

Dear Dr. Bosurgi,

*Many thanks for your letter of February 17^th^, 2020, regarding the above titled manuscript.*

*We have revised our manuscript extensively, based on your instructions, and the reviewers’ comments, and have also prepared a point-by-point response – indicating where we have agreed, and occasionally, disagreed (reviewers’ comments in blue and responses in black below). Note that Line numbers refer to the clean version!*

*We hope the revised manuscript is now acceptable for publication in PLOS Medicine, and look forward to hearing from you.*

*Yours very truly,*

*Dick Menzies and Mercedes Yanes-Lane,*

*On behalf of all authors*

Response to the reviewers:

**Reviewer #1:**

Thank you for the opportunity to review this paper. I note that I am not a statistician and I recommend that a statistical reviewer also looks at this piece. This manuscript presents a further network meta-analysis (NMA) of difference treatments for latent tuberculosis infection (LTBI), this time focused on people living with HIV (PLHIV). The authors' emphasis on the value of rifamycin-containing regimens is overly strongly expressed at various points in the piece, considering the weight of the evidence presented and the fact that much of it is from rifampin-containing regimens, which are contraindicated by drug-drug interactions in PLHIV. Sadly, rifapentine-containing regimens, which the authors drift towards in the discussion, are out of reach in terms of cost in high-HIV and TB burden settings. As the World Health Organization's (WHO)'s recommendation is that 6 months of isoniazid is the standard of care for such individuals, the author's current grouping of 6-12 month isoniazid regimens together limits the use of their work for revising these guidelines. Indeed, WHO recommends that this six month regimen is used for both PLHIV and those who are HIV negative; in this context the author's emphasis on the use of rifamycin-containing regimens specifically among people for whom drug-drug interactions are of concern reads oddly without further discussion.

MAJOR

ABSTRACT

**1)**	**Consistency of baseline- it would aid the reader if all comparisons were made to one baseline regimen (see comment on baselines later), such that a better feel for the results can be obtained. At the moment, it is very hard to see how the results feed into the sentence 'Rifamycin-containing regimens appear safer and at least as effective as isoniazid regimens in preventing TB and death and should be considered part of routine care in PLHIV' due to this inconsistency.**

We have modified the abstract to compare all others to the 6 to 12 H regimens. “According to the network meta-analysis, 6 to 12 months of isoniazid were no more efficacious in preventing microbiologically confirmed TB than rifamycin-containing regimens (incidence rate ratio [IRR] 1.1, 95% CI 0.8 to 1.45), however, 6 to 12 months of isoniazid were associated with a statistically significant higher incidence of all-cause mortality (IRR 1.6, 95% CI 1.2 to 2.0), and with a higher risk of grade three or higher hepatotoxicity (risk difference 8.8 95% CI 2.8 to 14.9).” Lines 18-24, page 2.

**INTRODUCTION**

**1)**	**Line 40- you state that WHO 'considers six months of isoniazid as the preferred TPT regimen for PLHIV'; it is worth noting here that WHO does not differentiate their recommendation by HIV status. (Additionally, please record a) WHO's recommendation of 36 months of isoniazid preventative therapy (IPT) among PLHIV in high incidence and transmission settings for TB (which ties into your sentence on line 42) and b) WHO's statement that 'Rifampin- and rifapentine-containing regimens should be prescribed with caution to people living with HIV who are on ART because of potential drug-drug interactions'.)**

Thank you for this suggestion. We updated the entire introduction according to the 2020 WHO recommendations (World Health Organization. WHO consolidated guidelines on tuberculosis. Module 1: prevention – tuberculosis preventive treatment. Geneva: 2020. Available from https://www.who.int/publications/i/item/who-consolidated-guidelines-on-tuberculosis-module-1-prevention- tuberculosis-preventive-treatment).

**2)**	**One of the selling points that the authors state in the cover letter for this piece is that 'the network meta-analysis is also novel, as prior reviews have conducted more traditional meta-analyses'. In fact, several NMAs have been conducted of treatment regimens for LTBI (they are a very established technique) e.g. Pease BMC ID 2017, Zenner Ann Intern Med 2017, Stagg Ann Intern Med 2014. The authors should cite these papers in the introduction of their paper, as they provide methodological precedence for their work as they narrow down the population of interest to people living with HIV.**

Thank you for this suggestion. We cited the above mentioned previous network meta-analysis. Introduction, line 56, page 4.

**3)**	**Given the two points above, the others also need to clearly define the need for a new NMA specifically among HIV positive individuals.**

After citing the findings of previous network meta-analysis, in which TPT is compared to placebo or no treatment, and in light of recent studies highlighting the benefits of rifamycin based regimens (and the inclusion of said regimens as acceptable treatments in PLHIV), we outline the need for a NMA in PLHIV. “In light of this new evidence, the WHO updated their guidelines in 2020 to include the use of three months of daily isoniazid plus rifampicin (3HR) and three months of weekly isoniazid plus rifapentine (3HP) as the preferred TPT regimens along with 6-9H, regardless of HIV status [...] However, the number of head-to-head comparisons between isoniazid based and rifamycin-containing regimens among PLHIV is very low. Hence, we aimed to assess the effectiveness and safety of different TPT regimens among PLHIV through a systematic review and network meta-analysis including clinical trials comparing a TPT regimen with either placebo, no treatment, or other treatment regimens.” Introduction lines 45-61, pages 4-5.

**METHODS**

**4)**	**Line 72- Please clearly define your population of interest- were studies in children included? How did you set criteria around whether participants had definite LTBI?**

We further specified our target population in the methods section. Data Sources and Searches Section in Methods, lines 71-81, page 6.

**5)**	**Line 78- 'Studies were excluded if there was no direct comparison of interest or if the only comparison included regimens using pyrazinamide and/or ethambutol as they are no longer recommended for TPT.' Regimens that are no longer of interest can provide important information in sparse data networks within NMAs, even if you would not consider the regimen itself as a treatment. Please justify the exclusion of these studies in this light.**

We agree with this suggestion, since NMAs are partly based on indirect comparisons (e.g., in a NMA including of two studies comparing A vs B and A vs C, the contrast of interventions B and C through the common node A would be possible through indirect comparisons) (*Reference: https://training.cochrane.org/handbook/current/chapter-11*). Therefore, we reviewed all the studies we had previously excluded because of no comparison of interest. For this revision we excluded studies only if they had no comparison of two or more regimens of interest, ***and also if*** indirect comparisons of two or more regimens of interest would not be possible. In other words, included studies had at least two regimens of interest OR at least one regimen of interest plus a comparator that would allow us to make indirect comparisons with at least one other study. For example, a study comparing daily isoniazid given for six months (a regimen of interest) vs. 2 months of daily rifampin plus pyrazinamide (a regimen not of interest), would have been included ONLY if there were another study comparing 2 months of daily rifampin plus pyrazinamide against another TPT regimen of interest (e.g., 36H). In this case we could include these two studies as this combination of regimens would allow us to make indirect comparisons. We updated our methods section accordingly. Study selection section in Methods, pages 6-7.

Because of this change, we now included two reports from one study (Gordin, 2004 and Gordin, 2000) comparing 12 months of isoniazid to 2 months of daily rifampin plus pyrazinamide. Furthermore, we added a detailed description of the exclusions because no regimens of interest into our results section (Results section) and we described more fully the reasons for exclusion of excluded studies in the Supplementary Table 3.

**6)**	**Line 86- you state that you adapted the Rob2 tool- please say how and why.**

We modified the ROB 2 tool for simplicity with the aim of increasing its inter-reader agreement. To do this, we chose the most relevant signaling questions from each RoB domain (based on expertise knowledge). Note that we used these quality assessments only for descriptive purposes and did not stratify our NMA according to the RoB. The selected questions for each domain are depicted in the supplementary table 2. We updated our methods section accordingly. Data Extraction and Quality Assessment in the Methods section, lines 104-114, pages 7-8.

**7)**	**Line 93- 'A study was classified as low risk of bias when there was low risk in the randomization process and low risk in at least two of the four remaining domains. If these conditions were not met, a study was considered at high risk of bias.' Please state if this is the normal approach for Rob2 and, if not, why you chose this approach.**

The original RoB2 tool includes predefined algorithms that help reviewers to perform a RoB judgement. However, since we simplified the tool, we also modified our RoB algorithm with the aim of reflecting the most important sources of bias to our research question. It is important to note that the RoB2 guidance states that RoB judgements can be modified by review authors if it is justified ([RoB 2: A revised Cochrane risk-of-bias tool for randomized trials | Cochrane Bias](https://methods.cochrane.org/bias/resources/rob-2-revised-cochrane-risk-bias-tool-randomized-trials)). We included a statement into our manuscript explaining that this was a pre-specified criterion. Data Extraction and Quality Assessment in the Methods section, lines 100-101, page 7.

**8)**	**Line 118- was the decision to run a random effects analysis made a priori? What was the decision-making behind this?**

Based on a prior NMA by Zenner et al we anticipated clinical (e.g., level of immunosuppression, type of ART) and methodological heterogeneity (e.g., outcomes assessment methods) among the included studies. Therefore, we made an a priori decision to perform a random-effects methods NMA to account for heterogeneity between studies. Data Synthesis and Statistical Analysis in the Methods section, lines 139-144, page 9.

**9)**	**Line 118- as this is a medical journal, please explain the graph-theoretical approach to NMAs (and justify this choice of a frequentist approach, as opposed to a Bayesian one) and net heat plot method.**

We have included the following explanation “Briefly, the graph theoretical approach uses a frequentist method to calculate NMA effect estimators. It is based on electrical theory in which variance corresponds to resistance, treatment effects to voltage, and weighted treatment effects to current flows. When applied to a pairwise meta-analysis, it can produce both random effects and fixed effects models. This method has been found to be equivalent to Bayesian mixed treatment comparisons.” We also included a brief description of net heat plots “This method assesses the contribution of each study design to inconsistency in the network by temporarily removing each design and calculating the differences in inconsistency. It uses colors within a matrix in order to graphically display the level of inconsistency from each design.” See Data Synthesis and Statistical Analysis in the Methods section (Lines 145-150 and 154-159, pages 9-10). Hopefully these descriptions help clarify what we did for the readers of this medical journal.

**RESULTS**

**10)**	**5 full texts not available is quite substantial given that you only included 15 trials. What size were these studies and what regimens did they examine (are they in table S3?)**

We contacted a librarian who helped us to locate the missing papers; four were located and all were excluded. In the following lines please find the reasons for exclusion (we updated Figure 1, the main text, and our supplementary table of reasons for exclusions accordingly):

1. Mukwiza-Kaponda, M. (2015); Southern African Journal of Epidemiology and Infection: Excluded as this was a Conference paper.
2. Sudre, P.	(1997); Revue d Epidemiologie et de Sante Publique: Excluded as was not a randomized trial, but was a prospective cohort study.
3. No authors listed	(1999);	Journal of Respiratory Diseases: Excluded as this was a Commentary and no original data provided.
4. Johnson Jr, W. D. (1994); Annals of internal medicine: Excluded as this was a Commentary (editorial) regarding a clinical trial published in the same issue.
5. “No authors listed (1996);J Int Assoc Physicians AIDS Care”. We have not been able to find this publication up to today, despite the help of the Librarian. However, this paper was not included in previous systematic reviews, including the prior NMA of Zenner et al. so we view it as unlikely that this was a randomized trial, that would have met our inclusion criteria.

**11)	Figure 2- this figure was too fuzzy to read within the submission.**

We incorporated a higher quality image into the manuscript.

**12)**	**Table 1: 5 out of your 15 studies had either no-one confirmed as LTBI positive or less than 10%. A further 5 had less than 50%. This is a large concern when it comes to your findings.**

As stated in the 2018 LTBI WHO guidelines, all adults and adolescents living with HIV with an unknown or a positive TST and unlikely to have active TB should receive TB preventive therapy, meaning that an LTBI test is not required prior to starting TPT. Hence, these studies followed current guidelines, and by including them, this enhances the applicability of our finding in settings following those guidelines. While we agree that the benefit for PLHIV with a negative LTBI test is lower, this difference is considered less important in high TB incidence settings, which contributed the majority of the included study populations. The effect of this finding in our analysis is that it might reduce power for efficacy as active TB events may be less likely but should not have impacted other outcomes. We did perform sensitivity analysis (Supplementary table 18), and showed greater benefit if LTBI test positive among studies of populations who had received either TST or IGRA. We have added mention of these results “In the stratified analysis by TST/IGRA status, the incidence of microbiologically confirmed or clinically diagnosed TB (Supplementary Table 18), was statistically significantly lower among TST/IGRA positive PLHIV with 24 or 36H and with rifamycin containing regimens when compared to placebo. However, TST/IGRA negative or anergic PLHIV, there was no statistically significant difference in incidence with these TPT regimens compared to placebo.” (Results Lines 360-364) and in the discussion “In our analysis, incidence of all forms of TB was significantly lower among PLHIV with a positive TST/IGRA test who received prolonged H regimens or rifamycin containing regimens when compared to placebo. This benefit was not seen among TST/IGRA negative or anergic individuals. Although inferences are limited as this analysis included only six trials, this finding is in accordance with a previous meta-analysis of treatment of latent TB infection among PLHIV[3], and reflects a higher risk of reactivation of TB among PLHIV with evidence of latent TB infection.[59] This finding reinforces the message that TST or IGRA are potentially useful to identify PLHIV at greater risk of disease[59], and who will derive greater benefit from TPT while also identifying those with negative tests who have lower likelihood of benefit, yet run the same risk of adverse events from TPT.[50, 60] Current guidelines encourage testing for latent TB infection, while also stipulating that this should not become a barrier, hence is not an absolute requirement for initiating TPT among PLHIV” (Lines 439-449).

**13)**	**S2 Fig- how does this map to the groupings under my query about line 145?**

This graph shows all the individual regimens that were included in our study. The lines represent the direct comparisons between regimens. We considered it a more detailed representation of all the connections in the network.

**14)**	**Given that the WHO recommends 6 months of isoniazid at the standard of care treatment and the sizeable number of trials using this regimen, I would strongly recommend making this the baseline throughout your analyses, rather than a mixed grouping.**

We think grouping 6 to 12 months of INH is appropriate since these are standard recommended regimens, and commonly used in clinical practice (although 12H less so now). As stated by the LTBI WHO guidelines, previous analyses have found no significant differences in efficacy among mono-therapy with INH given from 6 or 12 months. But we also made all comparisons for individual regimes (e.g., 6H and 9H separately), in the Supplementary Table 13. We have referred to these results “Sensitivity analyses by individual TPT regimens (Supplementary Tables 13 to 16), show similar results to the primary analyses. Thirty-six months of isoniazid was statistically significantly better at preventing microbiologically confirmed active TB when compared to 1HP. Whereas 3HR was statistically significantly better at preventing all-cause mortality when compared to placebo or no treatment, 6H, and 36 H. For the outcome of all-cause mortality among rifamycin-containing regimens, when studies with high rates of ART were excluded (Swindells et al), the protective effect of rifamycin-containing regimens remained unchanged.” (Results, lines 352-358)

**15)**	**Table 2- Your estimate for the efficacy of 6 to 12 H at preventing active TB vs Placebo is in itself quite uncertain- 0.8 (0.5 to 1.1). How do you explain this?**

Thank you for bringing up this observation. We hypothesize that the reason why there is a high degree of uncertainty around this estimate is the long duration of follow-up post treatment and the high incidence of TB within the study settings. Other studies (Churchyard et al 2014; Mwinga et al 1998; Gordin et al 1997; Johnson et al 2001; Samandari et al 2011) have found that the protective effect of H in high burden settings, may only be maintained during treatment, after which re-infection could occur. This is likely one driver of uncertainty around the efficacy of 6 to 12H in preventing TB when compared to placebo. It is also important to note that when stratifying our analysis according to the TST status, we found evidence of benefit when using 6 to 12 H regimens relative to placebo (incidence rate difference comparing of 0.7, 95%CI 0.4 to 1). Therefore, TST status could also have contributed to the uncertainty in our results.

**16)**	**Table 3- can you hypothesize why none of the isoniazid regimens are effective against all-cause mortality and the implications of this for interpreting your isoniazid result? How good is rifamycin at preventing death in comparison to 6 month isoniazid regimens in studies where ART use was high (i.e. the reverse analysis of S17 table- see comment regarding line 357)? In terms of the rifamycin vs. 6-12m isoniazid result, what does this relative rate translate to in terms of absolute rates?**

Comparing mortality with rifamycin regimens vs 6 to 12 INH, the rate difference was -0.25 (95%CI –1.28 to 0.79). This estimate has a wide range of uncertainty, so inferences must be cautious. We have puzzled over, and discussed among ourselves the findings related to mortality, but have not found an epidemiological or clinical confounding factor (ART use, the degree of immunosuppression of the included subjects, year of publication, etc.) that would explain this. In particular, we examined the use of ART in studies grouped by whether they included a rifamycin arm and not - and found no difference in proportion with any ART use, nor in the median use of ART in the two groups of studies. In the study by Swindells et al, when comparing 1HP to 9INH among participants on ART, there were 6 deaths in the 1-month group and 10 in the 9-month group, with an incidence rate difference of –0.08. This was the only study comparing INH to rifamycin within a population with a high prevalence of ART use, and when this study was excluded the NMA estimates of mortality were the same. We do not disregard the possibility that these results could reflect selection bias due to differential loss to follow up (i.e., it could be the case that patients with shorter regimes had higher rates of loss to follow up relative to those with longer regimes and, therefore, censored more frequently). But if anything one would expect that the longer time post treatment associated with the shorter rifamycin regimens would bias towards finding higher mortality. In the end, we are not sure, so the findings are presented in the hope of stimulating further study of a potentially important benefit, while expressing caution about this finding. We have included the following text in the discussion “There is not an obvious reason as to why rifamycin containing regimens reduce all-cause mortality but not TB incidence when compared to isoniazid. However, rifamycin is a sterilizing drug, which may have a bigger effect on preventing TB deaths. Unfortunately, most studies reported all-cause mortality, thus we were not able to stratify by TB related or unrelated deaths.” Discussion section, lines 403-407, page 34.

**17)**	**You chose not to calculate ranks within your analyses, but as a matter of interest how did rifamycin-containing regimens rank throughout?**

In response to this, and Reviewer 2, we have now included a new Table 7, with the ranking of treatments for each outcome in the NMA. Extended H regimens topped the ranking for preventing microbiologically confirmed TB, while rifamycin containing regimens where ranked top for preventing death and had lowest risk of grade three or higher hepatotoxicity. Page 30.

**18)**	**Line 332- 'higher completion rates'- this is not substantiated by the data. Shorter regimens have higher completion rates, but not specifically rifamycin-containing ones.**

We agree, and modified this statement to explain that rifamycin containing regimes are the shortest regimens available to date, and have been found to have higher completion rates relative to longer regimens. Results section, line 314-315, page 27.

**19)**	**Line 357- pleas state Swindells' result regarding the risk of death for isoniazid and rifampin-containing regimens.**

Thanks for this suggestion. We have added “In the study by Swindells et al, there were 6 deaths in the 1HP group and 10 in the 9-month INH group, with an incidence rate difference of –0.08., This was not statistically significant (log-rank p-value: 0.31).” Discussion section, lines 395-397, page 34.

**20)**	**Line 404- greater depth as to the fact that WHO does not recommend rifamycin-containing regimens in people living in HIV is required here. Please start by describing this and state the context of these concerns for rifampin, noting that most of your data is from rifampin-containing regimens. Given the frequency of use of these affected ART regimens globally, the lack of ability in many settings where TB-HIV is particularly common to alter the dose of ART, and the cost of rifapentine vs. rifampin please caveat the real-world applicability of your findings.**

Thank you for this comment. Although previously WHO guidelines had favoured INH regimens, the 2020 WHO guidelines were very different, and recommended two rifamycin containing regimens to be considered ‘equal’ to INH regimens, including for PLHIV. Therefore, we added the following “Currently, WHO considers two rifamycin-containing regimens (3HR, 3HP) as equally preferred as 6H or 9H, regardless of patients’ HIV status^12^. According to our results, rifamycin-containing regimens are as efficacious as 6 to-12H in preventing active TB, may be more effective in preventing death, and have a lower risk of liver toxicity. However, an important potential limitation of rifamycin containing regimens among PLHIV is the risk of drug-drug interactions^12^, particularly with ART. For example, a recent study in healthy volunteers using a combination of dolutegravir and rifapentine was stopped because of high rates of adverse drug reactions.^61^ However, another recent study among PLHIV found that 3HP could be safely administered along with dolutegravir .^62^ The combination of a rifamycin and efavirenz does not require dose adjustments.^63^ Since the pharmacokinetics effects of anti-TB therapy on ART (and vice-versa) are highly variable (often requiring dose adjustments), further consultation of updated resources is advised when prescribing rifamycin-containing regimens in PLHIV.^63, 64^ Drug costs for rifamycin-containing regimens may be greater than for isoniazid, but one study found that total health system costs (including personnel, lab costs, etc) were lower with 4R than 6H or 9H, in low-, middle- and high-income settings.” Discussion section, lines 450-465, pages 36-37.

**21)	Line 416- 'Given this, rifamycin-containing regimens should be considered the regimens of choice for TPT in PLHIV.' This statement is very strong in light of how much of your data comes from rifampin trials, the uncertainty around the impact of rifapentine containing regimens on mortality from table S5, and the concerns about rifampin and ARTs. Please alter.**

We have modified to the following statement “The findings of this review support the recommendations by the WHO, and the CDC that rifamycin-containing regimens are acceptable for TPT in PLHIV.” Conclusion section, lines 482-484, page 37.

**SUPPLEMENTS**

**22)**	**Supplementary Material A- why did you search for 'LTBI therapy' etc. but not spell out latent tuberculosis (latent tuberculosis infection, latent TB, latent TB infection)? This could have been a source of missing studies. It would been easier to have used rows 10 to 18 as treatment terms only without need to specify LTBI in here (given that you did so as an independent item in rows 2 and 4).**

Thank you for the suggestion. We ran the search with the MESH term Latent Tuberculosis spelled out and found no difference in the results. Moreover, since LTBI therapy commonly refers to many regimes, we decided to include variations of this term in lines 10 to 18.

**23)**	**Supplementary Material A- given that all trials of LTBI therapy will have examined at least one of your outcomes of interest, why did you include rows 20-25 of your search? As these terms are not always explicit in titles, abstracts, and key words, this could have led to missing studies.**

As stated in the methods section, we included these outcomes of interest as additional rows in our search strategy. However, we repeated our search, but deleted lines 20 to 26. We retrieved a total of 8898 unique records. To assess the yield of the new search strategy we randomly selected a sample of 1000 titles and abstracts from this new search. We screened these in duplicate (ie two authors) for studies matching our inclusion criteria, and no additional studies were found. Therefore, we think that it's very unlikely that our search strategy missed any relevant clinical trial.

**MINOR**

**ABSTRACT**

**24)**	**The authors repeatedly use the word 'significantly' throughout the text. Use of this word should be made cautiously given potential confusion between the colloquial and the statistical. In the latter case, use of p-value thresholds alone to mark out important results for causal analyses has largely been debunked; relative effect sizes, confidence intervals and sample size should all be interpreted together.**

We removed the word significantly from the abstract and, when necessary, referred to the CI.

**METHODS**

**25)**	**Cochrane Database of Systematic Reviews- why did you choose to search a database of reviews, as opposed to a database of trials?**

We apologize for this. We actually searched the Cochrane library, which includes information about trials. We corrected this in our methods section accordingly.

**26)**	**The authors should check that their manuscript lines up against the PRISMA guidelines for NMAs, rather than the standard PRISMA guidelines.**

Thank you for pointing this out. We used the PRISMA guidelines for NMAs. We added the PRISMA checklist.

**27)**	**Line 145- 'mono-H (6 to 72 months of H), 6 to 12 months of H, 24 to 72 months of H, and rifamycin-containing regimens (3HR, 3HP, and 1HP).' This sentence is unclear as the first grouping overlaps with the subsequent two.**

We modified this sentence as follows: “In order to compare all mono-isoniazid regimens against rifamycin containing regimens, we created a node with all with all mono-isoniazid regimens (i.e., from 6 to 72 months of isoniazid), and another with and all rifamycin- containing regimens (i.e., 3HR, 3HP, and 1HP). After that, we divided mono-isoniazid regimens where then divided into two different nodes based on treatment durations (i.e., 6 to 12 months of isoniazid and, 24 to 72 months of isoniazid)”. Data synthesis and analysis section, methods, lines 175-180, page 10.

**28)**	**Line 161- stRAtified.**

We have corrected accordingly.

**RESULTS**

**29)**	**Line 318- were, not where.**

We have corrected accordingly.

**SUPPLEMENTS**

**30)**	**Supplementary Material A- please number the rows of your search so the AND and OR rows make sense.**

We have corrected accordingly.

**31)**	**S2 Table- please define N (I assume this is no) and PN.**

We have defined N (no) and PN (probably no) in S2.

**Reviewer #2:**

Points of detail

**Page 3 I think this is the first occurrence of H as an abbreviation. I did manage to work out what it means and it is explained on page 11. You TB folk certainly have a way with abbreviations, H = isoniazid?**

We apologize for this. All abbreviations used for drug regimens have now been described in the introduction. We also limited the use of letters to refer to TPT regimes.

**Page 4 I think excluding observational studies hardly needs justifying if there are enough trials.**

We appreciate the suggestion. Given that in our protocol, published in Prospero, we included observational studies, we consider it important to explain why we changed the approach when conducting the NMA. We consider it might be also useful to readers who may not be familiar with NMAs.

**Page 4 Various empirical studies have indicated that the main bibliographic databases have varied and incomplete coverage of material not in English (Pilkington et al., 2005), and have limited coverage of material from low and middle–income country journals (Kieling et al., 2009; Syed Sheriff et al., 2008). I am not a TB or HIV person but I understand they both have a major burden in LMIC so extending the search into one of other of the WHO sponsored databases might be helpful. The current search has brought enough studies to light but not so many that a few more would not be welcome.**

We followed your advice and conducted a new search in the Latin American and Caribbean Center on Health Sciences Information database (LILACS). We found 246 studies that were not identified in our previous search. After a duplicated and independent review of titles and abstracts, no additional eligible clinical trials were found.

**Page 4 Why exclude studies with fewer than 25 per arm? I agree they will be less precise but the analysis should take care of that.**

Thank you very much for this commentary. We decided this when writing our protocol under the assumption that the coefficients for the effect measures of interest would probably not be accurate enough. However, in fact, no study was excluded for this reason. Two studies had less than 25 participants per arm (PORTILLA, J., 2003 and MATTELI, 1999). However, both studies were excluded because they did not have two or more regimens of interest nor could we use them to make any indirect comparisons. We added these to the flow chart (now 157 studies reviewed for full text instead of 155) and we updated our supplementary table of reasons for exclusion to add these manuscripts, and why.

**Page 5 Perhaps for completeness say how the articles in languages other than English were dealt with. There does seem to have been at least one (reference 27).**

We have included the following statement “For studies in languages other than English, members of the team fluent in the language were asked to assess the eligibility of the study.” Study selection in methods, lines 97-98, page 7.

**Page 7 The only problem with not providing a ranking of treatments is that the reader is going to impose their own ranking on them anyway so it might be better to present something and stress the uncertainty to protect the reader from over–exuberant extrapolation.**

Thank you for this suggestion, also suggested by Reviewer 1. Please see the response to comment #17 from Reviewer 1.

**Page 8 Providing the software versions is seldom done but helps reproducibility.**

Thank you, we have ensured the version of both the packages and the software are included.

**Page 9 The full list of excluded studies is a valuable addition.**

Thanks.

**Page 13 Table 2 I do not grasp what the sentence ‘For the study by Martinson et al, 3HP and 3HR arms were grouped together, for all other studies treatment arms remained the same.’ means. Specifically what does it mean for an arm to remain the same?**

We apologize for the confusion. We have now excluded the 3HR arm of Martinson et al given that it was an intermittent scheme. The sentence the reviewer refers to has been removed.

**Page 15, Table 3 I think I have missed something here as the 40% figure in the caption does not seem to be reflective of anything in the body of the table.**

This was a mistake in our writing. The sentence was : “the incidence of all-cause mortality with rifamycin containing regimens was 40% that of the incidence with placebo”, and is now: “…was 40% less than that with placebo”. Results section, line 300-301, page 24.

**References The authors’ bibliographic management software seems to have had problems with references 24 and 25 and maybe some others, I did not do an exhaustive check.**

Thank you for this correction. We reviewed and modified the references accordingly.

**Figure 1 The caption seems to duplicate information which is in the box for exclusions. A new abbreviation for isoniazid is introduced here too.**

Thank you for this correction. We simplified figure one and added details on the reasons for excluding studies classified as “with no comparison of interest” is presented in the supplementary material.

**Supplementary heatmaps. Two of these did not come through properly although that often seems to happen to me with figures in Word documents perhaps because I use LibreOffice. I assume they will be OK in the final production version. Figure S5 did come through although was not that informative.**

We have increased the resolution of figures to adhere to journal guidelines.

**I think there are some advantages in using what netmeta calls network league tables for Tables 2, 3, 4 and equivalent in the supplement. A publication quality example can be found in Barth et al. (2013, Table 3). The advantage is that all comparisons involving a particular condition are adjacent. In table 2 for instance the 6 to 12 H comparisons are not adjacent nor are the 24 to 72 H.**

To address this suggestion, we created a new table using “netmeta network league tables” (following the example that you provided), and for comparison, we included our original and the new table below. We respectfully consider that our original version (showing direct, indirect and network meta-analysis estimates in columns) might be easier to understand for the average reader, so we have retained the original table style in the R1 version.

***Table for Reviewer****. Incidence Rate Ratios of microbiologically confirmed and clinically diagnosed active TB throughout study duration, by aggregated TPT regimens. Conventional meta-analysis (upper triangle) and network meta-analysis (lower triangle).*

|  | **24 to 72H IRR (95%CI)** | **Placebo IRR (95%CI)** | **Rifamycin containing IRR (95%CI)** | **6 to 12H IRR (95%CI)** |
| --- | --- | --- | --- | --- |
| **24 to 72H IRR (95%CI)** | -- | 0.77 (0.42 to1.43) | 0.55 (0.19 to 1.56) | 0.57 (0.31 to 1.04) |
| **Placebo IRR (95%CI)** | 0.54 (0.34 to 0.85) | -- | 2.01 (0.93 to 4.32) | 1.54 (1.12 to 2.12) |
| **Rifamycin containing IRR (95%CI)** | 0.83 (0.48 to 1.44) | 1.54 (1.00 to 2.39) | -- | 0.93 (0.63 to 1.37) |
| **6 to 12H IRR (95%CI)** | 0.77 (0.49 to 1.21) | 1.43 (1.07 to 1.92) | 0.93 (0.64 to 1.35) | -- |

***Original Table 3.*** *Network meta-analysis of incidence of microbiologically confirmed and clinically diagnosed active TB throughout study duration, by aggregated TPT regimens.*

| **Comparison** | **Number of trials with direct comparison** | **Direct estimates IRR (95%CI)** | **Indirect estimates IRR (95%CI)** | **Network meta-analysis IRR (95%CI)** |
| --- | --- | --- | --- | --- |
| **Total number of comparisons (studies)** | 21 (15) |  |  |  |
| 6 to 12H vs Placebo | 7 | 0.6 (0.5 to 0.9) | 1 (0.5 to 2.2) | 0.7 (0.5 to 0.9) |
| 24 to 72H vs placebo | 2 | 0.8 (0.4 to 1.4) | 0.4 (0.2 to 0.7) | 0.5 (0.3 to 0.8) |
| 6 to 12H vs 24 to 72H | 2 | 1.8 (1 to 3.2) | 0.9 (0.4 to 1.7) | 1.3 (0.8 to 2) |
| 6 to 12H vs Rifamycin containing | 7 | 1.1 (0.7 to 1.6) | 1.1 (0.3 to 3.9) | 1.1 (0.7 to 1.6) |
| 24 to 72H vs Rifamycin containing | 1 | 0.6 (0.2 to 1.6) | 1 (0.5 to 1.8) | 0.8 (0.5 to 1.4) |
| Rifamycin containing vs Placebo | 2 | 0.5 (0.2 to 1.1) | 0.7 (0.4 to 1.3) | 0.6 (0.4 to 1) |

**Point of more substance: There are a number of possible diagnostics which can be run after the main analysis to check for things like inconsistency. If the authors feel, for some reason, that these are inappropriate then an explanation of why would be in order.**

Thank you for your suggestion. We made netheat plots in order to test for inconsistency. This is the approach most recommended by the developers of the model (Rücker et al 2012). These figures are included in the supplementary material (Supplementary Figures 3 and 4 and detailed in lines 153 to 157). We also measured the Q statistic, although these results were not included in the manuscript as we considered the netheat plots to be more informative.

Michael Dewey

**Reviewer #3:**

Thank you for the opportunity to review this important piece of work.

**a)**	**Line 51. Are you comparing phase III clinical trial efficacy or effectiveness (real conditions)? What degree of "pragmatism" do they have? In line 58 you say you exclude observational studies.**

Thank you for your question. Our systematic review and network meta-analysis was based on the intention to treat or modified intention to treat analysis results. Therefore, we were interested in treatment effectiveness (specified in lines 116 to 117). Although these are randomized controlled trials, the included studies have varying degrees of pragmatism, depending on the measures used to prevent loss to follow up and enhance treatment compliance. We excluded observational studies given that one of the assumptions of the NMA is transitivity, which requires that comparison groups must be similar in all relevant characteristics besides the interventions (Reference: <https://training.cochrane.org/handbook/current/chapter-11>).

**b)**	**Line 63. Why was clinical TB initially excluded? Should it be specified upfront in the protocol as primary and secondary endpoints (lab confirmed and treatment initiation, respectively). Could you still do that? Could you include table S18 within the main manuscript?**

Thank you for your suggestion. Our protocol specified three different TB outcomes:

1)Microbiologically confirmed TB incidence, included the number of patients confirmed using an acid-fast bacilli smear, culture, and/or molecular tests (Gene-Xpert®);

2) Incidence for all TB cases used total number of patients with clinical diagnosis (using X-ray abnormalities or suggestive histology (positive for necrotizing granulomas)) and microbiologically confirmed TB; and

3) Incidence of TB diagnosed by unspecified methods, used the number of TB patients with an undefined method of diagnosis.

Most studies reported their outcomes as microbiologically confirmed or a composite of microbiologically and clinically confirmed. Since we considered the first outcome to be more specific, we decided to present it as our main result. Nevertheless, we followed your advice and included a new table using as outcome the combination of microbiologically confirmed or clinically diagnosed TB. Please refer to Table 3 in the results section (Page 22).

**c)**	**Line 105. Do you think all-cause mortality an important outcome? Do you think that differences in mortality, when found, could be attributed to the regimen used? Could you expand the discussion a little bit on line 353 (I wonder why there is no difference in efficacy when using ryfamicin-based regimens, but there is on mortality).**

Thank you for this very relevant question. In fact, Reviewer 1 also raised this important point. Our answer to both reviewers is the following:

Comparing mortality with rifamycin regimens vs 6 to 12 INH, the rate difference was -0.25 (95%CI –1.28 to 0.79). This estimate has a wide range of uncertainty, so inferences must be cautious. We have puzzled over, and discussed among ourselves the findings related to mortality, but have not found an epidemiological or clinical confounding factor (ART use, the degree of immunosuppression of the included subjects, year of publication, etc.) that would explain this. In particular, we examined the use of ART in studies grouped by whether they included a rifamycin arm and not - and found no difference in proportion with any ART use, nor in the median use of ART in the two groups of studies. In the study by Swindells et al, when comparing 1HP to 9INH among participants on ART, there were 6 deaths in the 1-month group and 10 in the 9-month group, with an incidence rate difference of –0.08. This was the only study comparing INH to rifamycin within a population with a high prevalence of ART use, and when this study was excluded the NMA estimates were the same. We do not disregard the possibility that these results could reflect selection bias due to differential loss to follow up (i.e., it could be the case that patients with shorter regimes had higher rates of loss to follow up relative to those with longer regimes and, therefore, censored more frequently). In the end, we are not sure, so the findings are presented in the hope of stimulating further study of a potentially important benefit, while expressing caution that the finding may reflect bias or confounding. We have included the following text in the discussion “There is not an obvious reason as to why rifamycin containing regimens reduce all-cause mortality but not TB incidence when compared to isoniazid. However, rifamycin is a sterilizing drug, which may have a bigger effect on preventing TB deaths. Unfortunately, most studies reported all-cause mortality, thus we were not able to stratify by TB related or unrelated deaths.” Discussion section, lines 403-407, page 34.

**d)**	**Line 111. It would be nice to have a table with all the different measures of adherence used in the studies (supp material is fine).**

Thank you for this suggestion. We have included a new supplementary table with that includes for each study the completion rates per treatment arm, their definitions for completion, and the methodology used for adherence assessment. (Supplementary Table 19, page 57).

**e)**	**How would the frequency of visits, or screening for TB to all participants might have changed the efficacy of the different regimens? How did you account for different PT duration follow up?**

We did not find enough information on support measures and intensity of screening for TB, to include in the analysis. However, we did stratify results according to post-treatment follow-up time as this was described in all studies (Please refer to the Supplementary Tables 9,10,13, and 15). However, this stratified analysis was limited by the low number of studies in each strata, resulting in wide and overlapping confidence intervals. This makes it difficult to make meaningful conclusions. However, we agree that studies that followed patients for longer periods of time after treatment are more likely to capture higher numbers of incident TB cases as time progresses especially in high incidence settings (Churchyard et al 2014; Mwinga et al 1998; Gordin et al 1997; Johnson et al 2001; Samandari et al 2011), and that is why we emphasized on the differences of follow-up between different studies (lines 263 to 267).

**f)**	**Line 161, Typo "stratified".**

Thank you for this correction. We modified it accordingly.

**g)**	**Table 1. Could you include the definition of the primary efficacy endpoint of each trial?**

We have included a new column (the last one) in table 1 (Page 15) with the information required.

**h)**	**Table 2. Could you include the references of studies included for each comparison (all tables)? For studies including composite endpoints, like Swindells' 1HP, did you exclude the clinically diagnosed participants?**

We had a secondary outcome that was all TB forms (i.e., microbiologically confirmed and clinically diagnosed) for studies that used a composite endpoint. We have also included a table with the analysis for this outcome, Table 3, results section. The references for studies used for each outcome have also been included throughout the text (lines 269 to 270; 20 to 281; 293 to 294; 304 to 305 and 320).

**i)**	**Table 3. Here I understand you are you including all studies independently of the ART status/CD4 count status. Related comment: Line 357. I understand that % or ART among arms is similar if only RCT were included. Your explanation on its role as confounder should contradict an even distribution of participants on ART. Am I interpreting correctly?**

You are correct that we did not use ART status or CD4 cell counts as inclusion or exclusion criteria. Although within each study the distribution of ART or CD4 by arm was similar, across studies the proportion of patients on ART varies widely (which may complicate the transitivity assumption for indirect comparisons). We included the following sentence in the results section “The proportion of studies that reported any use of ART was the same for studies with an isoniazid arm (9/13) or a rifamycin-containing arm (4/6) (excluding pyrazinamide containing regimens). The median proportion of ART use reported in studies with an isoniazid arm was 36.3% (range, 0 to 100%), and in studies with a rifamycin-containing regime was 25% (range, 0 to 96.3%).” (Results section, lines 258 and 261). Although this analysis is limited, we considered that ART was not likely to have been a major confounder for mortality.

**j)**	**No comments in the discussion about the efficacy of PTs when outcomes include clinical TB diagnosis (stemming from table S18)**

We have included the following comment on the efficacy of TPT for the outcome of microbiologically confirmed and clinically diagnosed TB, as shown in Table 3 “In this analysis, there appears to be no difference between different grouped regimens in preventing microbiologically confirmed active TB or microbiologically and clinically diagnosed TB”. Discussion section (lines 408 to 410).

**k)**	**Fig 2's quality is too low to be interpreted.**

We have increased the resolution of Figure 2.
