# Supplementary material for: Tuberculosis preventive therapy for people living with HIV: A systematic review and network meta-analysis
Source: PLoS Med. 2021 Sep 14;18(9):e1003738. doi: 10.1371/journal.pmed.1003738 (PMC8439495; doi:10.1371/journal.pmed.1003738)
Supplement: Attachment — Submitted filename: Response to reviewers_R2.docx [file pmed.1003738.s005.docx]

Montreal, 16 June 2021

REF: “"Tuberculosis Preventive Therapy for People Living with HIV: A Systematic Review and Network Meta-analysis" (PMEDICINE-D-20-06010R1)”

Dr Raffaella Bosurgi,

Executive Editor

Medicine

PLOS Medicine

plosmedicine.org

Dear Dr. Bosurgi,

Many thanks for your letter of June 9th, 2021, regarding the above titled manuscript.

We have revised our manuscript, based on your instructions, and have also prepared a point-by-point response – indicating where we have made changes (editors’ comments in blue and responses in black below).

We hope the revised manuscript is now acceptable for publication in PLOS Medicine, and look forward to hearing from you.

Yours very truly,

Dick Menzies and Mercedes Yanes-Lane,

On behalf of all authors

Response to editorial comments

1. Please submit your revision as a research article.

We have submitted as a research article.

2. Please ensure that you comply with PLOS' data policy (<https://journals.plos.org/plosmedicine/s/data-availability>) in your revision.

We have submitted an excel file with the from the included papers, that we used in our analyses. We hope this is what you are looking for - since this is a meta-analysis, and not an original study - but these are the data necessary for analysis.

3. Please remove the information on funding from the title page. In the event of publication this information will appear in the article metadata, via entries in the submission form.

We have removed all information regarding funding from the manuscript.

4. In the abstract and throughout the text, please quote p values alongside 95% CI, where available.

We have added the p values alongside 95%CI throughput the manuscript

5. Please add a new final sentence to the "Methods and findings" subsection of your abstract, beginning "Study limitations include ..." or similar and quoting 2-3 of the study's main limitations.

We have added the following sentence at the end of the methods section in the abstract “Study limitations include potential confounding due to differences in post-treatment follow-up time and TB incidence in the study setting on the estimates of incidence of TB or all-cause mortality, as well as an under representation of pregnant women and children.”

6. After the abstract, please add a new and accessible "author summary" in non-identical prose. You may find it helpful to consult one or two recent research papers published in PLOS Medicine to get a sense of the preferred style.

We have added the following author summary:

Why was this study done?

- People living with HIV (PLHIV) have a high risk of developing and dying of tuberculosis (TB) disease.
- There are several TB preventive treatment regimens (TPT) available for PLHIV (i.e., 6, 12 and 36 months of isoniazid (6H, 12H, 36H), 3 months of isoniazid plus rifampin (3HR), and 1 or 3 months of isoniazid plus rifapentine (1HP, 3HP) ).To date there are no direct comparisons between these preventive treatments.

What did the researchers do and find?

- We performed a systematic review and network meta-analysis (NMA) of studies comparing 2 or more of the currently recommended TPT regimens among PLHIV.
- The NMA approach allowed us to use data from published randomized controlled trials to indirectly compare different TPT strategies.
- Shorter, rifamycin-containing regimens (3HR, 3HP, 1HP) had better completion rates than isoniazid-based regimens (6H, 12H, 36H).
- When compared to rifamycin-containing regimens, isoniazid based regimens were associated with a higher mortality risk and a higher frequency of severe adverse events.
- Rifamycin-containing regimens were as good as Isoniazid-based regimens at preventing TB disease, and there was no evidence that they contributed to development of drug resistance.

What do these findings mean?

- Rifamycin-containing regimens are as safe and effective as isoniazid-based regimens in PLHIV and should be considered part of routine care.
- Further research is needed to define which rifamycin-containing regimen is most beneficial in PLHIV.

7. Please remove the "Role of the funding source" section, noting the comment above.

We have removed all mention of funding from the manuscript

8. Please use the style "... 12 clinical trials" throughout the text, although numbers should be spelt out at the start of sentences.

We have made the changes accordingly.

9. Throughout the text, please restyle the reference call-outs as follows: "... darunavir) [12,13]." (i.e., preceding punctuation and with no spaces within the square brackets).

We have made the changes accordingly.

10. Noting reference 3 and others, please ensure that competing interest information is removed from all citations.

We have removed the competing interest information from all references.

11. Is reference 4 lacking a report number?

We have added the report number.

12. Are references 19 and 50 lacking journal names? Reference 53 may also need attention.

We have added the missing journal names.

13. Please reformat the author lists for references 40 and 42.

We have reformatted the author list.

14. Reference 63 may need additional access information.

We have provided the missing information.

15. Please abbreviate journal names consistently in the reference list, and use the abbreviation "PLoS ONE".

We have made the changes accordingly.

16. Please rename the PRISMA attachment "S1_PRISMA_Checklist" or similar, and refer to it by this label in the Methods section.

We have updated accordingly, although NNA appears at start to help keep the 3 PRISMA checklists together with the appropriate study.

17. In the checklist, please refer to individual items by section (e.g., "Methods") and paragraph numbers, not line or page numbers as these generally change in the event of publication.

We have updated accordingly.
