# Supplementary material for: Tuberculosis preventive therapy for people living with HIV: A systematic review and network meta-analysis
Source: PLoS Med. 2021 Sep 14;18(9):e1003738. doi: 10.1371/journal.pmed.1003738 (PMC8439495; doi:10.1371/journal.pmed.1003738)
Supplement: Attachment — Submitted filename: NMA REsponseToReviewers R4.docx [file pmed.1003738.s006.docx]

Dr Raffaella Bosurgi,

Executive Editor

Medicine

PLOS Medicine

plosmedicine.org

June 24, 2021.

Dear Dr. Bosurgi:

Thank you very much for your commentaries on our original article entitled "Tuberculosis Preventive Therapy for People Living with HIV: A Systematic Review and Network Meta-analysis" (PMEDICINE-D-20-06010R3) Yanes-Lanes, et al. We addressed all of them before submitting this revision. Please find below this letter the answer to all of the requests.

Sincerely,

Dr. Dick Menzies, for all authors,

Respiratory Epidemiology and Clinical Research Unit,

McGill International TB Centre, McGill University

**Requests from Editors:**

1. Please add a sentence, say, to the abstract to summarize study characteristics, e.g., median sample sizes and regions where the studies were done.

**Answer**: We included the following information in the abstract “*The median sample size was 616 (interquartile range , 317 to 1,892). Eight were conducted in Africa, 3 in Europe, 3 in the Americas, and 2 included sites in multiple continents.*” (Lines 18-21)

2. Throughout the text, please remove spaces from the reference call-outs (e.g., "... PLHIV [63,64].").

**Answer**: We removed the spaces throughout the text accordingly.

3. We suggest using the spelling "graphically" (Methods).

**Answer: W**e changed this word accordingly (Line 198).

4. At line 132, should that be "... did not compare 2 or more ..."?

**Answer:** Thank you very much for this correction. We modified this sentence accordingly: “Studies were excluded if they did not compare 2 or more TPT regimens of interest” (Line 135).

5. Please remove trademarks, e.g., at line 163.

**Answer:** We removed the trademark accordingly (Line 166).

6. Please spell out the author names for reference 38, and any other relevant references.

**Answer**: Authors were spelled-out (i.e., *Gordin FM, Cohn DL, Matts JP, Chaisson RE, Brien RJ, Terry Beirn Community Programs for Clinical Research on AIDS, the Adult AIDS Clinical Trials Group, and the Centers for Disease Control and Prevention*), (Line 717).

7. Please use the journal name abbreviation "PLoS ONE".

**Answer**: We modified reference 46 accordingly (Line 747).

**Comments from Academic editor:**

**1.** It has come to our attention that a published systematic review and meta-analysis focused on IPT for PLHIV on ART (Ross et al, Lancet ID https://www.thelancet.com/journals/lanhiv/article/PIIS2352-3018(20)30299-X/fulltext). We did not see this cited in your article

**Answer:** Thank you for pointing out this paper, which was published in January of this year. We have now cited this paper in our discussion: “An individual patient meta-analysis of data from three studies, evaluating daily INH with ART to ART alone, found that the combined treatments did not have a statistically significant impact in reducing all-cause mortality when compared to ART alone [57].” (Lines 442-444).

2. Your NMA of different regimens is welcome and a contribution to the literature, but we wonder whether there is effect modification due to ART status and suspect many of our readers will wonder the same given that the included data features cohorts not receiving ART from the pre-ART era, which is no longer the clinical norm. We therefore ask that you investigate the prospect of effect modification due to ART formally, presenting those findings in the MS, and if appropriate presenting separate effect estimates based on ART status.

To respond to your second point - this took a bit longer! We investigated the possibility of effect modification using two strategies:

First, we used meta-regression to assess the effect of the different TPT regimens on all-cause mortality and microbiologically confirmed tuberculosis, **adjusted** for the proportion of ART use (defined as ever use). We used the method described by Lumley for meta-regression in a network meta-analysis (*Lumley T. Network meta-regression for indirect treatment comparisons. Stat Med 2002;21: 2313F24*) The results from this analysis are reported in Response Tables 1-4, which are below - in this response. As you can see, the point estimates assessing the effect of the aggregated TPT regimens on all-cause mortality and microbiologically confirmed TB were almost identical to the unadjusted NMA results (Table 1 and Table 2, respectively). The results were also not substantially in the analysis of individual treatment regimens when comparing the adjusted, to the original unadjusted results (Tables 3 and 4).

Second, we looked at potential effect modification of ART by conducting a stratified NMA of the aggregated TPT regimens on all-cause mortality and microbiologically confirmed TB. We stratified by ART use, using a cut-off of more or less than 50% of participants who had received ART. This cut-off was used given that the proportion of ART use ranged from 0 to 100% between the trials (Table 1 of the manuscript). As seen in Tables 5 and 6, there was no meaningful difference in the estimated IRR for the aggregated regimens in the two strata. Confidence intervals were wider, reflecting the smaller number of studies in each strata, particularly the strata with >50% ART use, but the point estimates were very similar.

In summary, both methods to examine the potential effect or effect modification of ART use on regimen efficacy revealed no evidence of any such effect. We have added a statement about the stratified analyses in methods, and results, plus added Tables 5 and 6 to the Supplement. We have added mention of the adjusted meta-regression using the Lumley method - to results plus added the citation for this method. But we have not added Tables 1-4 (shown below) anywhere, so simply stated ‘data not shown’ in the text.

**See Methods**: Lines 234-237 we added: *“...., a stratified analysis of studies in which more, or less than 50% of participants had received ART, and meta-regression, adjusting for proportion of study participants who had ever received ART using the method described by Lumley (23) .”*

**In Results**: Lines 394-401 we added: “*When studies were stratified on the basis of whether more, or less than 50% of participants had ever received ART, point estimates of the effect of aggregated regimens on mortality, and microbiologically TB were very similar in the two strata, although confidence intervals were wider, due to the smaller number of studies within each strata (Supplemental Tables 20 and 21). When adjusted for proportion of study participants who had received ART, using meta-regression for network meta-analysis, the adjusted results for aggregated and individual regimens were very similar to the unadjusted results (data not shown in tabular form).”*

**Response Tables for Reviewers and Editors:**

**Table 1.** Network meta-analysis of incidence of **all-cause mortality** throughout study duration, by **aggregated TPT regimens**.

| Comparison | Number of trials with direct comparisons | Direct estimates  IRR (95%CI) | Indirect estimates  IRR (95%CI) | Network meta-analysis  IRR (95%CI) | Adjusted for ART use*  IRR |
| --- | --- | --- | --- | --- | --- |
| Total number of comparisons (studies)* | 27 (16) |  |  |  |  |
| 6 to 12 H vs Placebo | 7 | 1.0 (0.8 to 1.1) | 1.0 (0.6 to 1.6) | 1.0 (0.8 to 1.1) | 1.0 |
| 24 to 72 H vs Placebo | 2 | 1.2 (0.7 to 2.0) | 0.9 (0.6 to 1.3) | 1.0 (0.7 to 1.4) | 1.0 |
| 6 to 12 H vs 24 to 72 H | 2 | 1.0 (0.7 to 1.5) | 0.8 (0.5 to 1.3) | 1.0 (0.7 to 1.3) | 1.0 |
| 6 to 12 H vs Rifamycin containing | 7 | 1.6 (1.3 to 2.1) | 1.2 (0.5 to 2.7) | 1.6 (1.2 to 2.0) | 1.6 |
| 24 to 72 H vs Rifamycin containing | 1 | 1.0 (0.4 to 2.3) | 1.8 (1.2 to 2.7) | 1.6 (1.2 to 2.4) | 1.6 |
| Rifamycin containing vs Placebo | 2 | 0.6 (0.4 to 0.8) | 0.7 (0.5 to 1.1) | 0.6 (0.5 to 0.8) | 0.6 |

Adjusted for proportion receiving ART in each study, using meta-regression for Network meta-analysis using methods suggested by Lumley (see references)

**Table 2.** Network meta-analysis of incidence of **microbiologically confirmed active TB** throughout study duration, by **aggregated TPT regimens**.

| Comparison | Number of trials with direct comparison | Direct estimates  IRR (95%CI) | Indirect estimates  IRR (95%CI) | Network meta-analysis  IRR (95%CI) | Adjusted for ART use*  IRR |
| --- | --- | --- | --- | --- | --- |
| Total number of comparisons (studies)* | 22 (13) |  |  |  |  |
| 6 to 12 H vs Placebo | 4 | 0.7 (0.5 to 1.1) | 1.1 (0.4 to 3.1) | 0.7 (0.5 to 1.1) | 0.8 |
| 24 to 72 H vs Placebo | 2 | 0.7 (0.2 to 2.2) | 0.4 (0.2 to 0.8) | 0.5 (0.3 to 0.8) | 0.5 |
| 6 to 12 H vs 24 to 72 H | 2 | 1.6 (1 to 2.5) | 1.2 (0.4 to 3.5) | 1.5 (1.0 to 2.3) | 1.6 |
| 6 to 12 H vs Rifamycin containing | 6 | 1.1 (0.8 to 1.5) | 0.5 (0.1 to 2.0) | 1.0 (0.8 to 1.4) | 1.1 |
| 24 to 72 H vs Rifamycin containing | 1 | 0.5 (0.2 to 1.3) | 0.8 (0.4 to 1.4) | 0.7 (0.4 to 1.2) | 0.7 |
| Rifamycin containing vs Placebo | 1 | 1 (0.2 to 4.4) | 0.7 (0.4 to 1.2) | 0.7 (0.4 to 1.2) | 0.8 |

* Adjusted for proportion receiving ART in each study, using meta-regression for Network meta-analysis using methods suggested by Lumley (see references)

**Table 3**. Network meta-analysis of **all-cause mortality** by **individual TPT regimens** adjusted for ever use of ART.

| Comparison | Number of study arms with direct comparison | Direct estimates IRR (95%CI) | Indirect estimates IRR (95%CI) | Network meta-analysis IRR (95%CI) | Adjusted for ART use*  IRR |
| --- | --- | --- | --- | --- | --- |
| 6H vs placebo | 5 | 1.0 (0.8 to 1.1) | 2.7 (0.5 to 14.8) | 1.0 (0.8 to 1.1) | 1.00 |
| 9H vs placebo | 0 | -- | 0.6 (0.1 to 2.2) | 0.6 (0.1 to 2.2) | 0.6 |
| 12H vs placebo | 2 | 0.9 (0.5 to 1.5) | 0.6 (0.3 to 1.2) | 0.8 (0.5 to 1.2) | 0.8 |
| 24H vs placebo | 2 | 1.2 (0.7 to 2.1) | -- | 1.2 (0.7 to 2.1) | 1.3 |
| 36H vs placebo | 0 | -- | 1.0 (0.6 to 1.6) | 1.0 (0.6 to 1.6) | 1.0 |
| 72H vs placebo | 0 | -- | 0.6 (0.3 to 1.5) | 0.6 (0.3 to 1.5) | 0.7 |
| 3HR vs placebo | 2 | 0.6 (0.4 to 0.8) | 0.6 (0.3 to 1.4) | 0.6 (0.4 to 0.8) | 0.6 |
| 3HP vs placebo | 0 | -- | 0.6 (0.3 to 1.2) | 0.6 (0.3 to 1.2) | 0.7 |
| 1HP vs placebo | 0 | -- | 0.3 (0.1 to 1.9) | 0.3 (0.1 to 1.9) | 0.3 |
| 6H vs 9H | 0 | -- | 1.7 (0.4 to 6.6) | 1.7 (0.4 to 6.6) | 1.7 |
| 6H vs 12H | 0 | -- | 1.2 (0.8 to 1.9) | 1.2 (0.8 to 1.9) | 1.2 |
| 6H vs 24H | 0 | -- | 0.8 (0.5 to 1.4) | 0.8 (0.5 to 1.4) | 0.8 |
| 6H vs 36H | 1 | 0.9 (0.6 to 1.4) | -- | 0.9 (0.6 to 1.4) | 1.0 |
| 6H vs 72H | 1 | 1.5 (0.7 to 3.4) | -- | 1.5 (0.7 to 3.4) | 1.5 |
| 6H vs 3HR | 3 | 1.8 (1.3 to 2.5) | 1.2 (0.5 to 2.7) | 1.7 (1.2 to 2.3) | 1.7 |
| 6H vs 3HP | 1 | 1.5 (0.8 to 2.9) | -- | 1.5 (0.8 to 2.9) | 1.5 |
| 6H vs 1HP | 0 | -- | 92. (0.5 to 15.7) | 2.9 (0.5 to 15.7) | 3.1 |
| 9H vs 12H | 0 | -- | 0.7 (0.2 to 3) | 0.7 (0.2 to 3) | 0.7 |
| 9H vs 24H | 0 | -- | 0.5 (0.1 to 2) | 0.5 (0.1 to 2) | 0.5 |
| 9H vs 36H | 0 | -- | 0.6 (0.1 to 2.3) | 0.6 (0.1 to 2.3) | 0.6 |
| 9H vs 72H | 0 | -- | 0.9 (0.2 to 3.9) | 0.9 (0.2 to 3.9) | 0.9 |
| 9H vs 3HR | 0 | -- | 1.0 (0.2 to 4) | 1.0 (0.2 to 4) | 1.0 |
| 9H vs 3HP | 1 | 0.9 (0.3 to 3) | -- | 0.9 (0.3 to 3) | 0.9 |
| 9H vs 1HP | 1 | 1.7 (0.6 to 4.7) | -- | 1.7 (0.6 to 4.7) | 1.9 |
| 12H vs 24H | 0 | -- | 0.6 (0.3 to 1.3) | 0.6 (0.3 to 1.3) | 0.6 |
| 12H vs 36H | 0 | -- | 0.8 (0.4 to 1.4) | 0.8 (0.4 to 1.4) | 0.8 |
| 12H vs 72H | 0 | -- | 1.2 (0.5 to 3.1) | 1.2 (0.5 to 3.1) | 1.2 |
| 12H vs 3HR | 1 | 0.7 (0.2 to 2.4) | 1.5 (0.9 to 2.6) | 1.4 (0.8 to 2.2) | 1.4 |
| 12H vs 3HP | 0 | -- | 1.2 (0.6 to 2.7) | 1.2 (0.6 to 2.7) | 1.2 |
| 12H vs 1HP | 0 | -- | 2.3 (0.4 to 13.5) | 2.3 (0.4 to 13.5) | 2.5 |
| 24H vs 36H | 0 | -- | 1.2 (0.6 to 2.4) | 1.2 (0.6 to 2.4) | 1.2 |
| 24H vs 72H | 0 | -- | 1.9 (0.7 to 5.1) | 1.9 (0.7 to 5.1) | 2.0 |
| 24H vs 3HR | 0 | -- | 2.1 (1.1 to 3.9) | 2.1 (1.1 to 3.9) | 2.3 |
| 24H vs 3HP | 0 | -- | 1.9 (0.8 to 4.5) | 1.9 (0.8 to 4.5) | 2 |
| 24H vs 1HP | 0 | -- | 3.6 (0.6 to 21.6) | 3.6 (0.6 to 21.6) | 4.1 |
| 36H vs 72H | 0 | -- | 1.6 (0.6 to 4) | 1.6 (0.6 to 4) | 1.5 |
| 36H vs 3HR | 0 | -- | 1.8 (1 to 3) | 1.8 (1 to 3) | 1.8 |
| 36H vs 3HP | 0 | -- | 1.6 (0.7 to 3.5) | 1.6 (0.7 to 3.5) | 1.5 |
| 36H vs 1HP | 0 | -- | 3 (0.5 to 17.5) | 3 (0.5 to 17.5) | 3.1 |
| 72H vs 3HR | 0 | -- | 1.1 (0.5 to 2.6) | 1.1 (0.5 to 2.6) | 1.1 |
| 72H vs 3HP | 1 | 1.0 (0.4 to 2.4) | -- | 1.0 (0.4 to 2.4) | 1.0 |
| 72H vs 1HP | 0 | -- | 1.9 (0.3 to 11.4) | 1.9 (0.3 to 11.4) | 2.0 |
| 3HR vs 3HP | 0 | -- | 0.9 (0.4 to 1.9) | 0.9 (0.4 to 1.9) | 0.9 |
| 3HR vs 1HP | 0 | -- | 1.7 (0.3 to 9.7) | 1.7 (0.3 to 9.7) | 1.8 |
| 3HP vs 1HP | 0 | -- | 1.9 (0.4 to 9.1) | 1.9 (0.4 to 9.1) | 2.0 |

* Adjusted for proportion receiving ART in each study, using meta-regression for Network meta-analysis using methods suggested by Lumley (see references)

**Table 4**. Network meta-analysis of **microbiologically confirmed TB** by **individual TPT regimens**, adjusted for ever use of ART.

| Comparison | Number of study arms with direct comparison | Direct estimates IRR (95%CI) | Indirect estimates IRR (95%CI) | Network meta-analysis  IRR (95%CI) | Adjusted for ART use*  IRR |
| --- | --- | --- | --- | --- | --- |
| 6H vs placebo | 3 | 0.8 (0.5 to 1.4) | 1.5 (0.3 to 8) | 0.9 (0.5 to 1.4) | 0.9 |
| 9H vs placebo | 0 | -- | 3.1 (0.5 to 18.8) | 3.1 (0.5 to 18.8) | 3.3 |
| 12H vs placebo | 1 | 0.5 (0.3 to 1.1) | 0.4 (0.1 to 1.8) | 0.5 (0.3 to 1.0) | 0.5 |
| 24H vs placebo | 2 | 0.7 (0.2 to 2.2) | -- | 0.7 (0.2 to 2.2) | 0.7 |
| 36H vs placebo | 0 | -- | 0.6 (0.3 to 1.2) | 0.6 (0.3 to 1.2) | 0.6 |
| 72H vs placebo | 0 | -- | 0.5 (0.2 to 1.5) | 0.5 (0.2 to 1.5) | 0.5 |
| 3HR vs placebo | 1 | 1.0 (0.2 to 4.4) | 0.6 (0.3 to 1.3) | 0.7 (0.4 to 1.3) | 0.7 |
| 3HP vs placebo | 0 | -- | 1.0 (0.4 to 2.2) | 1.0 (0.4 to 2.2) | 1 |
| 1HP vs placebo | 0 | -- | 4 (0.6 to 27.5) | 4.0 (0.6 to 27.5) | 4.5 |
| 6H vs 9H | 0 | -- | 0.3 (0 to 1.5) | 0.3 (0 to 1.5) | 0.3 |
| 6H vs 12H | 0 | -- | 1.7 (0.8 to 3.8) | 1.7 (0.8 to 3.8) | 1.9 |
| 6H vs 24H | 0 | -- | 1.2 (0.4 to 3.9) | 1.2 (0.4 to 3.9) | 1.3 |
| 6H vs 36H | 1 | 1.5 (0.9 to 2.6) | -- | 1.5 (0.9 to 2.6) | 1.5 |
| 6H vs 72H | 1 | 1.8 (0.7 to 4.8) | -- | 1.8 (0.7 to 4.8) | 1.8 |
| 6H vs 3HR | 3 | 1.3 (0.8 to 2.2) | 0.5 (0 to 5.9) | 1.3 (0.8 to 2.1) | 1.2 |
| 6H vs 3HP | 1 | 0.9 (0.5 to 1.7) | -- | 0.9 (0.5 to 1.7) | 0.9 |
| 6H vs 1HP | 0 | -- | 0.2 (0 to 1.4) | 0.2 (0 to 1.4) | 0.2 |
| 9H vs 12H | 0 | -- | 6.3 (0.9 to 41.4) | 6.3 (0.9 to 41.4) | 7.1 |
| 9H vs 24H | 0 | -- | 4.3 (0.5 to 34.7) | 4.3 (0.5 to 34.7) | 4.8 |
| 9H vs 36H | 0 | -- | 5.5 (0.9 to 33.4) | 5.5 (0.9 to 33.4) | 5.5 |
| 9H vs 72H | 0 | -- | 6.4 (1.0 to 41.7) | 6.4 (1.0 to 41.7) | 6.6 |
| 9H vs 3HR | 0 | -- | 4.6 (0.8 to 27.6) | 4.6 (0.8 to 27.6) | 4.6 |
| 9H vs 3HP | 1 | 3.2 (0.7 to 16) | -- | 3.2 (0.7 to 16) | 3.3 |
| 9H vs 1HP | 1 | 0.8 (0.4 to 1.6) | -- | 0.8 (0.4 to 1.6) | 0.7 |
| 12H vs 24H | 0 | -- | 0.7 (0.2 to 2.4) | 0.7 (0.2 to 2.4) | 0.7 |
| 12H vs 36H | 0 | -- | 0.9 (0.3 to 2.2) | 0.9 (0.3 to 2.2) | 0.8 |
| 12H vs 72H | 0 | -- | 1 (0.3 to 3.6) | 1.0 (0.3 to 3.6) | 0.9 |
| 12H vs 3HR | 0 | -- | 0.7 (0.3 to 1.8) | 0.7 (0.3 to 1.8) | 0.7 |
| 12H vs 3HP | 0 | -- | 0.5 (0.2 to 1.4) | 0.5 (0.2 to 1.4) | 0.5 |
| 12H vs 1HP | 0 | -- | 0.1 (0 to 0.9) | 0.1 (0 to 0.9) | 0.1 |
| 24H vs 36H | 0 | -- | 1.3 (0.4 to 4.8) | 1.3 (0.4 to 4.8) | 1.2 |
| 24H vs 72H | 0 | -- | 1.5 (0.3 to 7.2) | 1.5 (0.3 to 7.2) | 1.4 |
| 24H vs 3HR | 0 | -- | 1.1 (0.3 to 3.9) | 1.1 (0.3 to 3.9) | 1 |
| 24H vs 3HP | 0 | -- | 0.8 (0.2 to 3) | 0.8 (0.2 to 3) | 0.7 |
| 24H vs 1HP | 0 | -- | 0.2 (0 to 1.7) | 0.2 (0 to 1.7) | 0.2 |
| 36H vs 72H | 0 | -- | 1.2 (0.4 to 3.5) | 1.2 (0.4 to 3.5) | 1.2 |
| 36H vs 3HR | 0 | -- | 0.8 (0.4 to 1.7) | 0.8 (0.4 to 1.7) | 0.8 |
| 36H vs 3HP | 0 | -- | 0.6 (0.3 to 1.3) | 0.6 (0.3 to 1.3) | 0.6 |
| 36H vs 1HP | 0 | -- | 0.1 (0 to 1.0) | 0.1 (0 to 1.0) | 0.1 |
| 72H vs 3HR | 0 | -- | 0.7 (0.2 to 2.2) | 0.7 (0.2 to 2.2) | 0.7 |
| 72H vs 3HP | 1 | 0.5 (0.2 to 1.3) | -- | 0.5 (0.2 to 1.3) | 0.5 |
| 72H vs 1HP | 0 | -- | 0.1 (0 to 0.9) | 0.1 (0 to 0.9) | 0.1 |
| 3HR vs 3HP | 0 | -- | 0.7 (0.3 to 1.6) | 0.7(0.3 to 1.6) | 0.7 |
| 3HR vs 1HP | 0 | -- | 0.2 (0 to 1.2) | 0.2 (0 to 1.2) | 0.2 |
| 3HP vs 1HP | 0 | -- | 0.2 (0 to 1.4) | 0.2 (0 to 1.4) | 0.2 |

* Adjusted for proportion receiving ART in each study, using meta-regression for Network meta-analysis using methods suggested by Lumley (see references)

**Table 5 (Supplement Table 20)**. Effect of **aggregated TPT regimens** on **incidence of all-cause mortality**, stratified by the proportion of study participants receiving ART (above, or below 50%).

| Comparison | Number of study arms with direct comparisons | Direct estimates  IRR (95%CI) | Indirect estimates  IRR (95%CI) | Network meta-analysis IRR (95%CI) |
| --- | --- | --- | --- | --- |
| More than 50 % of participants received ART (ever) | | | | |
| Total number of comparisons (studies) | 5 (5) |  |  |  |
| 6 to 12 H vs Placebo | 3 | 0.8 (0.6 to 1.1) | -- | 0.8 (0.6 to 1.1) |
| 24 to 72 H vs placebo | 1 | 1.8 (0.9 to 3.6) | -- | 1.8 (0.9 to 3.6) |
| 6 to 12 H vs 24 to 72 H | 0 | -- | 0.5 (0.2 to 1) | 0.5 (0.2 to 1) |
| 6 to 12 H vs Rifamycin containing | 1 | 1.7 (0.6 to 4.9) | -- | 1.7 (0.6 to 4.9) |
| 24 to 72 H vs Rifamycin containing | 0 | -- | 3.7 (1 to 13.6) | 3.7 (1 to 13.6) |
| Rifamycin containing vs Placebo | 0 | -- | 0.5 (0.2 to 1.5) | 0.5 (0.2 to 1.5) |
| Less than 50 % of participants received ART (ever)* | | | | |
| Total number of comparisons (studies) | 22 (11) |  |  |  |
| 6 to 12 H vs Placebo | 4 | 1 (0.9 to 1.2) | 0.5 (0.3 to 1) | 1 (0.8 to 1.2) |
| 24 to 72 H vs placebo | 1 | 0.5 (0.2 to 1.3) | 1 (0.7 to 1.4) | 0.9 (0.6 to 1.2) |
| 6 to 12 H vs 24 to 72 H | 2 | 1 (0.7 to 1.5) | 1.9 (0.8 to 4.7) | 1.1 (0.8 to 1.5) |
| 6 to 12 H vs Rifamycin containing | 6 | 1.6 (1.2 to 2.1) | 1.3 (0.4 to 3.8) | 1.6 (1.2 to 2) |
| 24 to 72 H vs Rifamycin containing | 1 | 1 (0.4 to 2.3) | 1.6 (1 to 2.4) | 1.4 (1 to 2.1) |
| Rifamycin containing vs Placebo | 2 | 0.6 (0.4 to 0.8) | 0.8 (0.5 to 1.5) | 0.6 (0.5 to 0.8) |

* For studies that did not report ART use we assumed that participants did not receive ART if recruitment finished before the year 2000. Four studies from 3 trials fulfilled this definition: Rivero et al. 2003 and 2007 (Spain, finished recruitment in 1998), Johnson and Whalen et al. (Uganda, finished recruitment in 1995), and Hawken et al. (Kenya, finished recruitment in 1994).

**Table 6 (Supplement Table 21)**. Effect of **aggregated TPT regimens** on incidence of **microbiologically confirmed TB**, stratified by the proportion of study participants receiving ART (above, or below 50%).

| Comparison | Number of study arms with direct comparison | Direct estimates IRR (95%CI) | Indirect estimates IRR (95%CI) | Network meta-analysis IRR (95%CI) |
| --- | --- | --- | --- | --- |
| More than 50 % of study participants received ART (ever) | | | | |
| Total number of comparisons (studies)* | 4 (4) |  |  |  |
| 6 to 12 H vs Placebo | 2 | 0.5 (0.3 to 1) | -- | 0.5 (0.3 to 1) |
| 24 to 72 H vs placebo | 1 | 1 (0.3 to 3.1) | -- | 1 (0.3 to 3.1) |
| 6 to 12 H vs 24 to 72 H | 0 | -- | 0.5 (0.1 to 2.1) | 0.5 (0.1 to 2.1) |
| 6 to 12 H vs Rifamycin containing | 1 | 0.8 (0.4 to 1.6) | -- | 0.8 (0.4 to 1.6) |
| 24 to 72 H vs Rifamycin containing | 0 | -- | 1.4 (0.3 to 6.6) | 1.4 (0.3 to 6.6) |
| Rifamycin containing vs Placebo | 0 | -- | 0.7 (0.3 to 1.7) | 0.7 (0.3 to 1.7) |
| Less than 50 % of study participants received ART (ever)* | | | | |
| Total number of comparisons (studies) | 18 (9) |  |  |  |
| 6 to 12 H vs Placebo | 2 | 0.9 (0.5 to 1.6) | 0.6 (0.1 to 3.4) | 0.9 (0.5 to 1.5) |
| 24 to 72 H vs placebo | 1 | 0.2 (0 to 3) | 0.6 (0.3 to 1.1) | 1 (0.3 to 3.1) |
| 6 to 12 H vs 24 to 72 H | 2 | 1.6 (1 to 2.5) | 7.7 (0.8 to 73.8) | 1.7 (1.1 to 2.6) |
| 6 to 12 H vs Rifamycin containing | 5 | 1.2 (0.8 to 1.8) | 0.6 (0.1 to 2.4) | 0.8 (0.4 to 1.6) |
| 24 to 72 H vs Rifamycin containing | 1 | 0.5 (0.2 to 1.3) | 0.8 (0.4 to 1.5) | 1.4 (0.3 to 6.6) |
| Rifamycin containing vs Placebo | 2 | 1 (0.2 to 4.4) | 0.7 (0.4 to 1.4) | 0.7 (0.3 to 1.7) |

* For studies that did not report ART use we assumed that participants did not receive ART if recruitment finished before the year 2000. Four studies from 3 trials fulfilled this definition: Rivero et al. 2003 and 2007 (Spain, finished recruitment in 1998), Johnson and Whalen et al. (Uganda, finished recruitment in 1995), and Hawken et al. (Kenya, finished recruitment in 1994).
