# Supplementary material for: Tuberculosis preventive therapy for people living with HIV: A systematic review and network meta-analysis
Source: PLoS Med. 2021 Sep 14;18(9):e1003738. doi: 10.1371/journal.pmed.1003738 (PMC8439495; doi:10.1371/journal.pmed.1003738)
Supplement: Attachment — Submitted filename: NMA REsponseToReviewers R5.docx [file pmed.1003738.s007.docx]

Dr Raffaella Bosurgi,

Executive Editor

Medicine

PLOS Medicine

plosmedicine.org

July 13, 2021.

Dear Dr. Bosurgi:

Thank you very much for your commentaries on our original article entitled "Tuberculosis Preventive Therapy for People Living with HIV: A Systematic Review and Network Meta-analysis" (PMEDICINE-D-20-06010R3) Yanes-Lanes, et al. We addressed all of them before submitting this revision. Please find below a point by point response to comments, with line numbers corresponding to the clean version of the manuscript.

Sincerely,

Dr. Dick Menzies, for all authors,

Respiratory Epidemiology and Clinical Research Unit,

McGill International TB Centre, McGill University

**Requests from Editors:**

1. Our academic editor requested that you include an additional supplemental table for the results from the following statement: "When adjusted for proportion of study participants who had received ART, using meta-regression for network meta-analysis, the adjusted results for aggregated and individual regimens were very similar to the unadjusted results (supplemental table X)." (noting that PLOS journals do not allow "data not shown" statements).

**Answer**: Thank you for your commentary, we added supplementary tables 22 to 25 which include the analysis adjusted by meta-regression.

2. Please check the risk difference quoted as "8.8" in the abstract, line 26, which may be quoted as "8.9" in table 5.

**Answer**: Thank you for this correction. We replaced 8.8 with 8.9 in the Abstract (Line 26).

3. At line 333, for example, please adapt p values to "p<0.001" unless there is a specific statistical justification for quoting smaller exact values.

**Answer:** We modified p-values accordingly (Lines 333, 344).

4. Please avoid "p<0.0000", e.g. at line 344.

**Answer:** We substituted p<0.000 by p<0.001 (Line 344).

5. At line 409, should that be "However, for TST/IGRA negative ..."?

**Answer**: Thank you for this suggestion, we corrected this sentence as follows: “However, for TST/IGRA negative or TST anergic PLHIV…” (Line 409).

6. At line 416, please make that "statistically significantly more effective ...".

**Answer**: Thank you, we modified this accordingly (Line 416).

7. In reference 6, should that be "N Engl J Med"?

**Answer**: We modified reference 6 accordingly (Line 616).

8. Please revisit reference 64, where it appears that the author list needs trimming to 6 names, followed by "et al". Are you able to add full access details?

**Answer**: We modified reference 64 accordingly as follows Dooley K., Churchyard G., Savic R., Gupte A., Marzinke M., Zhang N., et al. Safety & PK of weekly rifapentine/isoniazid (3HP) in adults with HIV on dolutegravir. Conference on Retroviruses and Opportunistic Infections; Washington 2019. Available at: https://www.croiconference.org/abstract/safety-pk-weekly-rifapentineisoniazid-3hp-adults-hiv-dolutegravir/. Accessed June 2021. (Line 808).

**Comments from Reviewer 2:**

1. In response to the analysis requested by the academic editor I have no further suggestions to make. Since this is an essentially ecological analysis I am not too surprised that it did not reveal anything startling but it is still worth having if it forestalls negative comments.

**Answer:** Thank you for your commentaries since they helped to improve our manuscript.
